# Frequency modulation of a bacterial quorum sensing response

**DOI:** 10.1038/s41467-022-30307-6

**Published:** 2022-05-19

**Authors:** Vera Bettenworth, Simon van Vliet, Bartosz Turkowyd, Annika Bamberger, Heiko Wendt, Matthew McIntosh, Wieland Steinchen, Ulrike Endesfelder, Anke Becker

**Affiliations:** 1 Center for Synthetic Microbiology (SYNMIKRO), Philipps-Universität Marburg, Marburg, Germany; 2 Department of Biology, Philipps-Universität Marburg, Marburg, Germany; 3 Biozentrum, University of Basel, Basel, Switzerland; 4 Max Planck Institute for Terrestrial Microbiology, Marburg, Germany; 5 Department of Physics, Carnegie Mellon University, Pittsburgh, PA USA; 6 Department of Chemistry, Philipps-Universität Marburg, Marburg, Germany; 7 Present Address: Institut für Mikrobiologie und Biotechnologie, Rheinische Friedrich-Wilhelms-Universität Bonn, Bonn, Germany; 8 Present Address: Institut für Mikrobiologie und Molekularbiologie, Justus-Liebig-Universität Gießen, Gießen, Germany

**Keywords:** Bacteriology, Bacterial genetics, Single-cell imaging, Population dynamics

## Abstract

In quorum sensing, bacteria secrete or release small molecules into the environment that, once they reach a certain threshold, trigger a behavioural change in the population. As the concentration of these so-called autoinducers is supposed to reflect population density, they were originally assumed to be continuously produced by all cells in a population. However, here we show that in the α-proteobacterium *Sinorhizobium meliloti* expression of the autoinducer synthase gene is realized in asynchronous stochastic pulses that result from scarcity and, presumably, low binding affinity of the key activator. Physiological cues modulate pulse frequency, and pulse frequency in turn modulates the velocity with which autoinducer levels in the environment reach the threshold to trigger the quorum sensing response. We therefore propose that frequency-modulated pulsing in *S. meliloti* represents the molecular mechanism for a collective decision-making process in which each cell’s physiological state and need for behavioural adaptation is encoded in the pulse frequency with which it expresses the autoinducer synthase gene; the pulse frequencies of all members of the population are then integrated in the common pool of autoinducers, and only once this vote crosses the threshold, the response behaviour is initiated.

## Abstract (web summary)

Quorum-sensing bacteria produce and secrete autoinducers that trigger a behavioral change in the population when reaching a certain threshold. Here, Bettenworth et al. show that autoinducer synthase gene expression in *Sinorhizobium meliloti* occurs in asynchronous stochastic pulses, and that physiological cues modulate pulse frequency and, consequently, response behavior dynamics. Frequency-modulated pulsing in autoinducer synthase gene expression thus represents a time-based mechanism for information integration and collective decision-making.

## Introduction

Far-reaching behavioural changes in bacterial populations are often initiated as a reaction to small molecules that the cells themselves produce and release into their environment. These molecules accumulate while the population grows and, once they reach a certain threshold, trigger changes in gene expression leading to, e.g., bioluminescence, virulence or biofilm formation. As the respective molecules are self-produced, they were termed autoinducers, and the phenomenon was initially referred to as autoinduction^1^; as the triggered behaviours were assessed to be effective only when performed by a large enough group and the autoinducer concentration to indicate when this sufficient population size—the quorum—is reached, the far more popular term for the process now is ‘quorum sensing’^2^.

Based on their ascribed role as indicators of population density, autoinducers were originally assumed to be continuously produced by all cells in a population^3,4^. However, over the past decade several cases of cell-to-cell heterogeneity in autoinducer synthase or precursor gene expression have been reported: e.g., in expression of the *Listeria monocytogenes agr* operon encoding the autoinducer precursor AgrD^5^, and in expression of the autoinducer synthase genes *ahlI* in *Pseudomonas syringae*^6^, *traI* and *ngrI* in *Sinorhizobium fredii*^7^, and *sinI* in *Sinorhizobium meliloti*^8^. Furthermore, there is indication of heterogeneity in AHL synthase gene expression in *Pseudomonas putida*^9^. Both the precise nature of these heterogeneities—whether they represented stable subpopulations with distinct expression levels, or rather variations over time—and their molecular origins remained unclear, but their observation nevertheless indicated that the model of constitutive autoinducer production is not universally valid^10,11^.

Moreover, both biotic factors like nutrient availability or stress and abiotic factors like diffusion or flow have long been known to affect autoinducer-mediated regulation^3,12–15^: For instance, luciferase production and bioluminescence in *Aliivibrio fischeri* is delayed via catabolite repression of the autoinducer receptor gene in presence of glucose^16–20^. Similarly, autoinducer production and target gene expression in *Erwinia carotovora* are altered by the type of carbon source provided^21^, and activation of the *Pseudomonas aeruginosa las* and *rhl* quorum-sensing systems likewise varies depending on growth conditions^22^. It has therefore been repeatedly acknowledged that the term ‘quorum sensing’ represents an oversimplification^3,12–14^ and should be used with full appreciation of the many environmental factors influencing it^15,23^. Even functions alternative or complementary to cell-density sensing were proposed, ranging from simple sensing of diffusion rates^24^ to the integration of different cues like cell density, clustering and diffusion^25^, or nutritional status and stress^26^. Here we show how in the α-proteobacterium *S. meliloti* phenotypic heterogeneity in autoinducer synthase gene expression and physiological influences on quorum sensing are linked in a collective decision-making process in which the first represents the key for integration of the latter.

## Results

### Stochastic pulsing in a canonical LuxR-LuxI-type quorum sensing system

**Fig. 1:**
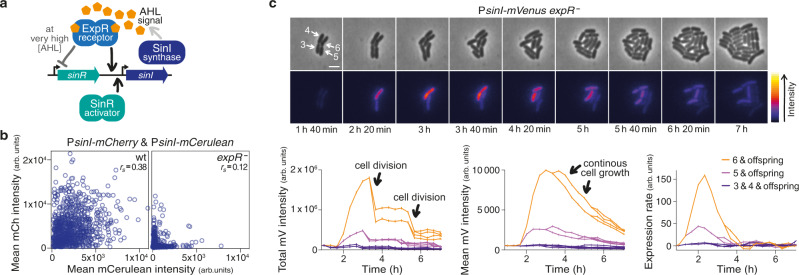
*sinI* expression is realized in stochastic pulses. **a** Simplified sketch of the regulatory network controlling AHL synthase gene expression in *S. meliloti*. **b** Fluorescence intensities from two *sinI* promoter-reporter gene fusions within individual cells determined by microscopy. Pooled data from 10 (wt) and 12 (*expR*^−^) colonies, respectively, imaged on 3 different days. Total number of cells analysed: *N* = 1190 (wt), 1287 (*expR*^−^). *r*_*s*_, Spearman’s correlation coefficient; *P* (two-tailed) <0.0001 for both data sets. See Supplementary Fig. 2a for raw images and details on the construct, Supplementary Fig. 2b for confirmation from an alternative construct. **c** (Top) Phase contrast and fluorescence images from a microscopy time lapse of an *expR*^−^ microcolony carrying a P*sinI*-*mVenus* fusion, and the ‘red fire’ lookup table applied to the fluorescence images. Data representative of 9 colonies imaged on 3 different days; scale bar, 2 µm. (Bottom) For cells #5 and #6, both total and mean fluorescence intensities first increase. Total fluorescence then drops with cell divisions and stays almost constant in between, while mean fluorescence constantly decreases with cell growth. Gene expression rate is calculated as the change in mean fluorescence intensity over time; peaks in gene expression rate are broadened by the regression involved in the calculation. See ‘Methods’ and Supplementary Fig. 3a for details and further examples.

*S. meliloti* is a widely-studied model organism for symbiosis with leguminous plants, but like other rhizobia it can also be found free-living in the soil. It has a canonical Gram-negative quorum sensing system homologous to the *A. fischeri* LuxR-LuxI system where LuxI is the synthase producing *N*-acyl homoserine lactones (AHLs) as autoinducers and LuxR is the cognate receptor, triggering the response upon AHL binding^2,4^. In the Sin system^27^ (Fig. 1a), the LuxI-type synthase SinI produces long-chain AHLs that are sensed by the LuxR-type regulator ExpR. However, the Sin system has an additional player: SinR, a LuxR-type regulator that, according to our analysis, has a degenerated AHL binding motif (Supplementary Fig. 1) and whose activity is not affected by AHLs^28^. Transcription of *sinI* strictly depends on SinR and is enhanced by binding of ExpR-AHL to the *sinI* promoter, giving rise to a positive feedback loop; at very high AHL concentrations, ExpR-AHL represses *sinR* transcription^29^.

As indicated above, expression of *sinI* in wild-type *S. meliloti* has been found to show strong cell-to-cell variation in fluorescence levels from a *sinI* promoter-fluorophore gene fusion^8^. To examine whether this variation reflects heterogeneity already present upstream in the regulatory network or rather stochastic processes inherent to *sinI* expression, we first generated strains carrying two identical copies of the *sinI* promoter fused to two different fluorophore genes. In these strains, upstream heterogeneity should affect both reporters to a similar degree within individual cells, whereas stochastic events during *sinI* expression should affect the two fusions independently and thus lead to uncorrelated variations^30,31^. Analysis of wild-type and *expR*^−^ strains carrying such constructs by microscopy snapshots (Fig. 1b and Supplementary Fig. 2a) and flow cytometry (Supplementary Fig. 2b) showed a considerable fraction of non-fluorescing cells in both backgrounds. Furthermore, in some wild-type cells variation in fluorescence affected both reporters to a similar extent, whereas other wild-type cells displayed highly diverging intensities from the two reporters. In *expR*^*−*^ cells, activation of the two promoter-fluorophore gene fusions was almost entirely uncorrelated, with most of the fluorescing cells showing fluorescence from either one or the other reporter. The overall low degree of correlation indicates that heterogeneity mainly stems from stochasticity inherent to *sinI* expression.

To further explore this stochasticity, we next followed *expR*^−^ microcolonies carrying a single *sinI* promoter-*mVenus* fusion via time-lapse microscopy (Fig. 1c, Supplementary Movie 1). Here, cells were usually dark, and when fluorescence appeared, it did so not in a coordinated fashion comprising the whole colony, but only in individual cells, and only temporarily. However, fluorescent reporters are stable proteins, and their levels thus reflect both current and past expression; to more accurately examine changes in *sinI* expression over time, we therefore calculated its expression rate adapted from Locke et al.^32^ (Fig. 1c, Supplementary Fig. 3a). This computational approach revealed unsynchronized sporadic pulses of *sinI* expression with a mean frequency of about 0.028 events per hour and cell (Supplementary Fig. 3b).

### A regulatory system based on very low odds

**Fig. 2:**
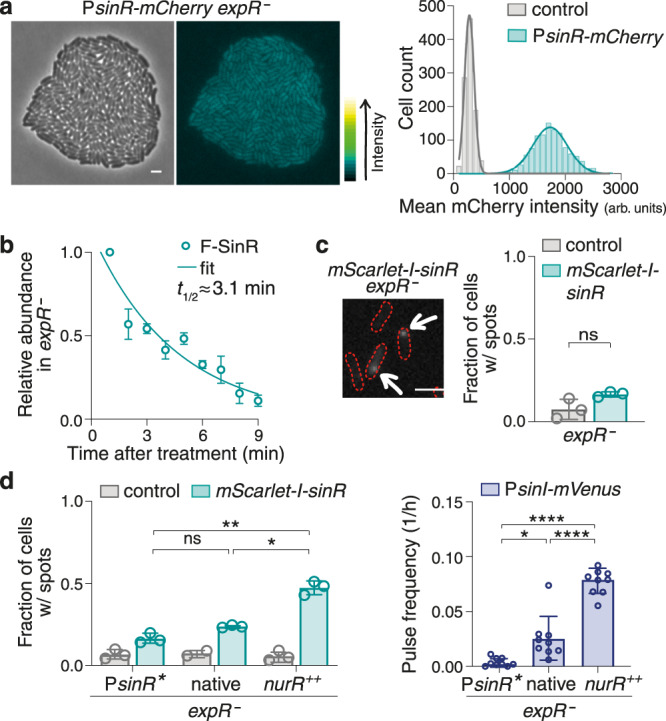
SinR scarcity is a key factor in *sinI* expression pulsing. **a** (Left) Raw phase contrast and fluorescence microscopy images of a strain carrying a *sinR* promoter-*mCherry* fusion, and the ‘green fire’ lookup table applied to the fluorescence image. Scale bar, 2 µm. (Right) Frequency distributions of mean mCherry intensities per cell and corresponding Gaussian fits suggest homogeneous *sinR* expression in the *expR*^−^ strain. Pooled data from snapshots of 7 and 6 colonies, respectively, imaged on 2 different days. *N* = 1077 (P*sinR-mCherry)*, 1004 (promoterless control). **b** Relative abundance of Flag-tagged SinR (F-SinR) after chloramphenicol treatment in 1-min intervals determined by Western blot analysis and a one-phase exponential decay fit to the data. Data, means ± standard deviations of 3 biological replicates. **c** (Left) Cut-out from a single-molecule microscopy snapshot of an *expR*^−^ strain expressing an *mScarlet-I-sinR* fusion from the chromosomal *sinR* promoter. Arrows mark fluorescing spots. (Right) Bar plots indicating the fraction of cells with fluorescing spots in this strain and the corresponding control strain lacking the fluorophore gene in 3 biological replicates; bars represent means ± standard deviations, open circles represent individual data. Statistical test, two-tailed unpaired *t*-test with Welch’s correction. ns, not significant; *P* = 0.1149. Total number of cells analysed: *N* = 2293 (*mScarlet-I-sinR*), 1670 (control). **d** Manipulation of (*mScarlet-I-)sinR* transcription levels yields corresponding patterns of (left) the fraction of cells displaying mScarlet-I-SinR spots in single-molecule microscopy and (right) P*sinI-mVenus* expression pulse frequencies in time-lapse fluorescence microscopy. PsinR*, promoter mutation resulting in reduced transcription; native, native promoter; *nurR*^++^, native promoter while overproducing its transcription activator NurR. Bar plots indicate means ± standard deviations and individual data from (turquois) single-molecule microscopy (SMM) performed in 3 biological replicates and (blue) time-lapse fluorescence microscopy of 9 colonies imaged on 3 different days. Statistical tests, Welch’s ANOVA tests with post hoc Dunnett’s T3 multiple comparisons test. ns, not significant; *, *P* < 0.05; **, *P* < 0.01; ****, *P* < 0.0001. Multiplicity-adjusted *P* values: PsinR* vs. native 0.1119, PsinR* vs. *nurR*^*++*^ 0.0012, native vs *nurR*^*++*^ 0.0217 for SMM data, 0.0290, <0.0001, <0.0001 for pulse data, respectively. Total number of cells analysed: *N* = 1260 (PsinR*), 1142 (native), 1158 (*nurR*^*++*^) for SMM data, 3411, 2900, 2440 for pulse data, respectively.

As intrinsic stochasticity was manifest both in the wild type and the *expR*^−^ strain, and was even more pronounced in the latter, we next investigated its most likely source—the essential transcription activator SinR—in the *expR*^*−*^ background. Expression of *sinR*, when assayed with a *sinR* promoter-*mCherry* fusion via microscopy, appeared rather weak and homogeneous (Fig. 2a), consistent with the above-drawn conclusion that heterogeneity in *sinI* expression does not originate upstream in the regulatory network. However, in vivo protein stability assays yielded a half-life of only about 3 min for a Flag-tagged SinR fusion protein when produced from the chromosomal *sinR* promoter (Fig. 2b, Supplementary Fig. 4a, b); and single-molecule microscopy of fixed cells carrying an *mScarlet-I-sinR* translational fusion at the chromosomal locus indicated that—after background subtraction—only about 10% of cells in a population, at a given time, have mScarlet-I-SinR spots (Fig. 2c; Supplementary Fig. 4c). Furthermore, when examining the effects of Flag-tagged SinR and mScarlet-I-SinR on the P*sinI-mVenus* reporter construct, the fusion proteins produced much higher fractions of fluorescing cells in flow cytometry measurements than native SinR (Supplementary Fig. 4d), suggesting that the latter is even less stable and/or abundant than its tagged versions. Such low protein abundance might seem unusual; however, a half-life of only 2 min has been reported for the *Agrobacterium tumefaciens* LuxR-type regulator TraR in absence of autoinducer^33^, and when Taniguchi et al.^34^ quantified the *Escherichia coli* proteome with a fusion protein library, an average copy number of less than 1 per cell was determined for about 40 of the 1018 proteins.

To test whether SinR scarcity is a determinant in *sinI* expression pulsing, we then generated two strains with slightly reduced and slightly increased *sinR* expression levels, respectively; the former by introducing a mutation into the *sinR* promoter interfering with binding of its transcription activator NurR^35^, the latter by overexpressing *nurR* from a plasmid. Single-molecule microscopy confirmed that the fraction of cells displaying mScarlet-I-SinR spots in the two strains was altered by the manipulations as intended, and time-lapse microscopy indeed yielded about 7-fold reduced and 3-fold increased *sinI* expression pulse frequencies, respectively (Fig. 2d and Supplementary Fig. 5a). When we repeated the analysis with different thresholds for what is considered a pulse, absolute pulse frequencies of course changed, but the relative differences between the strains remained (Supplementary Fig. 6). Furthermore, the fraction of fluorescent cells in flow cytometry measurements—a proxy for pulse frequency, as, e.g., a higher frequency over time in individual cells should produce a higher fraction of fluorescent cells in a population at a given time—was altered in a corresponding fashion (Supplementary Fig. 5b); *nurR* overexpression in a *sinR*^-^ or *sinR* promoter mutation background in turn did not affect this fraction (Supplementary Fig. 5c). In contrast, direct overproduction of (mScarlet-I-)SinR from a plasmid not only abolished heterogeneity in fluorescence both from the mScarlet-I-SinR and the P*sinI-mVenus* fusion, but also greatly increased fluorescence intensities (Supplementary Fig. 5d), disrupting the otherwise stochastic regulatory system. Thus, scarcity of SinR is indeed a determining factor for *sinI* expression in a pulsatile rather than a continuous fashion.

### Pulse frequency fine-tuned by physiological factors

**Fig. 3:**
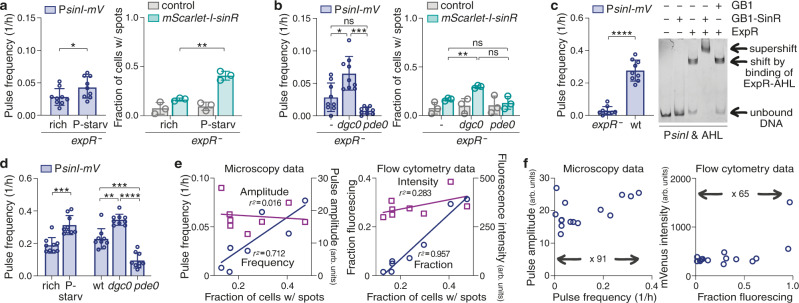
Physiological factors modulate pulse frequency by changing SinR abundance and DNA binding affinity. **a**, **b** Phosphate starvation (**a**) and cyclic-di-GMP levels (**b**) modulate both (left, respectively) the frequency of *sinI* expression pulses and (right, respectively) the fraction of cells displaying mScarlet-I-SinR spots in corresponding patterns. **c** (Left) The wild type with AHL-induced positive feedback likewise displays elevated *sinI* expression pulse frequency compared to the *expR*^−^ strain. (Right) EMSAs indicate that the AHL receptor increases SinR binding affinity to the *sinI* promoter: His-GB1-SinR does not induce a shift of *sinI* promoter DNA, but causes a supershift in the presence of His-ExpR-AHL. His-GB1-SinR, 70 µM; His-GB1, 70 µM; His-ExpR, 1 µM; AHL, 10 µM. EMSA representative of 3 independent experiments. **d** Modulation of *sinI* expression pulse frequency by (left) phosphate starvation and (right) c-di-GMP levels in the wild type. **a**–**d** Bar plots show means ± standard deviations and individual data from (turquois) single-molecule microscopy performed in 3 biological replicates and (blue) time-lapse fluorescence microscopy of 9 colonies imaged on 3 different days. Statistical tests, two-tailed unpaired *t*-tests with Welch’s correction for (**a**, **c**, **d** (left)); Welch’s ANOVA tests with post hoc Dunnett’s T3 multiple comparisons test for (**b**, **d** (right)). ns, not significant; *, *P* < 0.05; **, *P* < 0.01; ***, *P* < 0.001; ****, *P* < 0.0001. *P* values (multiplicity-adjusted, if appropriate): rich vs. P-starv in *expR*^−^, 0.0324 (pulse data), 0.0066 (SMM data) (**a**); *expR*^−^ vs *expR*^−^ *dgc0* 0.0146 (pulse data), 0.0012 (SMM data), *expR*^−^ vs. *expR*^−^ *pde0* 0.0661 (pulse data), 0.7482 (SMM data), *expR*^−^ *dgc0* vs. *expR*^−^ *pde0* 0.0003 (pulse data), 0.1039 (SMM data) (**b**); *expR*^−^ vs. wt, <0.0001 (pulse data) (**c**); rich vs^.^ P-starv in wt, 0.0002 (pulse data) (**d** (left)); wt vs. *dgc0*, 0.0010, wt vs. *pde0*, 0.0004, *dgc0* vs. *pde0* < 0.0001 (all pulse data) (**d** (right)). Total number of cells analysed: *N* = 2639 (rich in *expR*^−^), 2355 (P-starv in *expR*^−^) for pulse data, 2293, 2321 for SMM data, respectively (**a**); 2^,^517 (*expR*^−^), 2518 (*expR*^*−*^ *dgc0*), 2031 (*expR*^−^ *pde0*) for pulse data, 2293, 2251, 2761 for SMM data, respectively (**b**); 2331 (*expR*^−^), 1506 (wt) for pulse data (**c**); 1690 (rich in wt), 1240 (P-starv in wt), 1787 (wt), 2065 (*dgc0*), 1657 (*pde0*) for pulse data (**d**). **e** Summary of pulse data determined by (left) microscopy and (right) flow cytometry indicating a linear correlation between the fraction of cells with mScarlet-I-SinR spots and pulse frequency. Circles represent (left) pulse frequency or (right) fraction of cells fluorescing, squares represent (left) median pulse amplitude or (right) median fluorescence intensity, both plotted against the respective fraction of cells with mScarlet-I-SinR spots; lines represent corresponding linear fits to the data. *r*^2^ calculated from Pearson’s correlation coefficient; *P* (two-tailed) = 0.0085 for pulse frequencies, <0.0001 for fractions fluorescing, 0.7625 for pulse amplitude, 0.1746 for fluorescence intensity data. **f** (Left) Median pulse amplitude plotted over mean pulse frequency, and (right) median fluorescence intensity plotted over mean fluorescing fraction, in both *expR*^−^ and wild-type backgrounds. The increase in flow cytometry intensities corresponding with very large fluorescing fractions are not reflected in pulse data and possibly result from consecutive pulses that are still separated by time-lapse analysis, but add up in terms of total fluorescence intensities. See Supplementary Fig. 11 for raw data on pulse amplitudes and fluorescence intensities.

As we were able to modify P*sinI-mVenus* pulse frequencies artificially, we next explored whether pulse modulation also occurs physiologically. As mentioned above, effects of various biotic and abiotic cues on quorum sensing are well-established^3,12–14,26^, and pulse modulation might well represent a mechanism for integrating physiological information on the dynamic scale. Population-level studies in *S. meliloti* had shown *sinI* expression to be enhanced by phosphate starvation^29^, to be decreased by elevated levels of the mobile-to-sessile lifestyle-switch second messenger cyclic di-GMP (c-di-GMP)^36^, and, as mentioned above, to be enhanced by ExpR-AHL-mediated positive feedback in the wild type^29^. When we examined the effects of the respective growth conditions and genetic backgrounds at the single-cell level, phosphate starvation indeed increased *sinI* expression pulse frequency, the fraction of P*sinI-mVenus* fluorescing cells, and the fraction of cells with mScarlet-I-SinR spots in the *expR*^−^ background compared to rich growth conditions (Fig. 3a, Supplementary Fig. 7a, b); and an *expR*^*−*^ strain incapable of producing detectable amounts of c-di-GMP (*dgc0*)^36^ likewise showed increased *sinI* pulse frequency, fraction of fluorescing cells, and cells with mScarlet-I-SinR spots, while an *expR*^*−*^ strain producing elevated levels of c-di-GMP (*pde0*) (Supplementary Fig. 8) showed the reverse phenotype, namely reduced pulse frequency, a smaller fraction of fluorescing cells, and fewer cells with mScarlet-I-SinR spots (Fig. 3b, Supplementary Fig. 7c, d).

In the wild type capable of ExpR-AHL-mediated positive feedback, pulse frequency and flow cytometry fraction were even raised ~10-fold compared to the *expR*^−^ strain, to about 0.28 pulses per hour and cell (Fig. 3c, Supplementary Fig. 7e, f, Supplementary Movie 2). However, presence of *expR* did not increase the fraction of cells with mScarlet-I-SinR spots correspondingly (Supplementary Fig. 7g). Instead, we could detect a His-GB1-SinR-dependent supershift of the *sinI* promoter in electrophoretic mobility shift assays (EMSAs) in the presence of His-ExpR-AHL, but no shift by purified His-GB1-SinR alone even at high concentrations (Fig. 3c). First, this observation suggests a very low binding affinity for SinR alone—too low to be detectable by our assay, and a feature that very likely adds to the stochasticity of the system (see Supplementary Fig. 7h for in vivo functionality of His-GB1-SinR in *expR*^−^). Second, ExpR-AHL seems to achieve its positive feedback by facilitating binding of SinR to the *sinI* promoter, while both phosphate starvation and c-di-GMP levels modulate SinR abundance (see Supplementary Fig. 7i for confirmation of relative differences by western blot analysis), thus all fine-tuning the probability for a *sinI* expression pulse. Consequently, the frequency modulation by phosphate starvation and c-di-GMP levels observed in *expR*^−^ strains also occurred in the wild-type background, only at elevated levels (Fig. 3d).

Since large cell aggregates like biofilms are known to exhibit concentration gradients of oxygen, nutrients, and autoinducers^37,38^, we furthermore scrutinized two of our data sets (Figs. 2d and 3c) for a potential effect of a cell’s position within the colony, and for potential changes over the course of colony development, i.e., over time. The data set with different *sinR* expression levels did show a significant increase in *sinI* expression from colony edge to colony centre; however, these differences were small compared to the differences between the respective strains (Supplementary Fig. 9a). Moreover, no such effect could be detected for the data set comparing the *expR*^*−*^ strain with the wild type (Supplementary Fig. 9b). When the same data sets were subdivided into three observation periods, no change in pulse frequency was observed between the temporal subsets (Supplementary Fig. 9c, d). Thus, while in view of our other data both temporal and positional effects on *sinI* expression pulse frequency would be expected in larger cell aggregates, the colonies we analysed at the single-cell level are very likely too small to already comprise the necessary chemical gradients.

### A linear correlation between key activator abundance and pulse frequency

Based on our hitherto cumulated data, we furthermore sought to analyse the relationship between mScarlet-I-SinR abundance and *sinI* expression more deeply. Both the high degree of stochasticity observed in *sinI* expression (Fig. 1b) and the homogeneity observed in *sinR* expression (Fig. 2a) had already indicated that heterogeneity in *sinI* promoter activity does not originate upstream in the regulatory network, i.e., from cell-to-cell differences in *sinR* expression. Closer examination of the single-molecule microscopy data from all *mScarlet-I-sinR* strains (with exception of the overexpression strain) supports this conclusion, as it suggests that mScarlet-I-SinR spots do not contain higher-order multimers, but only one or two functional mScarlet-I molecules, and thus very likely only one or two SinR molecules (Supplementary Fig. 10). Moreover, all data gathered in the *expR*^−^ background indicates a linear correlation between the fraction of cells displaying mScarlet-I-SinR spots and frequency of *sinI* expression pulses (Fig. 3e; Supplementary Table 1). In contrast, pulse amplitude and fluorescence intensity as its corresponding property in flow cytometry data do not appear to correlate with SinR abundance. When plotting pulse amplitude against frequency for all *expR*^−^ and wild-type data, frequency increases about 91-fold over the whole data set, while amplitude varies only about 2-fold; flow cytometry data shows a similar trend, with a 65-fold change in the fraction of fluorescing cells, and a 6-fold change in intensity (Fig. 3f; Supplementary Table 1). Hence, regulation of *sinI* expression primarily happens through frequency modulation, and amplitude modulation plays a minor role at best.

### Response dynamics determined by pulse frequency

**Fig. 4:**
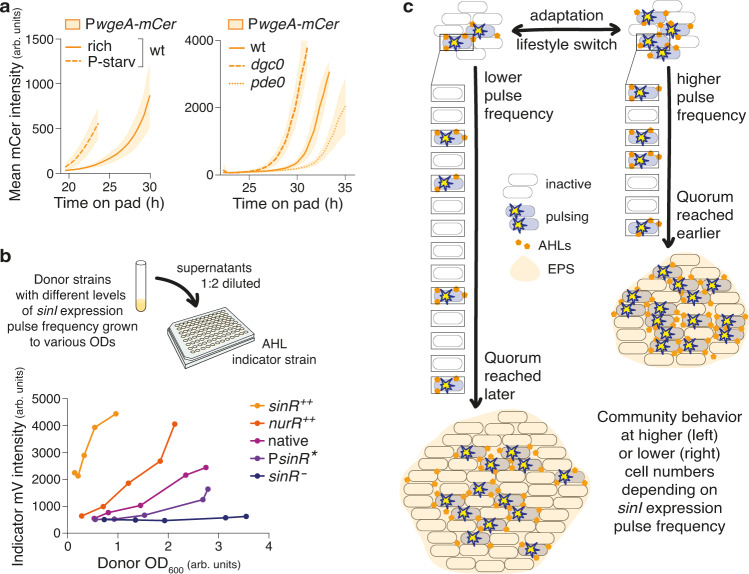
*sinI* expression pulse frequency determines quorum sensing response dynamics. **a** Different *sinI* expression pulse frequencies correlate with different quorum-sensing response dynamics, i.e., earlier or later activation of the EPS promoter P*wgeA*. Data, means ± standard deviations of (left) the same 9 colonies imaged on 3 different days as in Fig. 3d followed for approx. 10 more hours, and of (right) 6 colonies from 2 biological replicates in Fig. 3d followed for approx. 12 more hours; the 3 colonies from the third biological replicate show similar relative differences, but different absolute timing (Supplementary Fig. 12f) that could be due to, e.g., differences in AHL stability on the pad. **b** (Top) Experimental setup applied to test the effect of different *sinR* expression levels—and corresponding different *sinI* expression pulse frequencies—on quorum sensing response dynamics of an *S. meliloti* AHL indicator strain. (Bottom) The different donor strains need to grow to different optical densities (OD_600_) till their culture supernatants induce the positive feedback on the P*sinI*-*mVenus* fusion in the indicator strain, confirming differences in the amount of secreted AHLs as the origin of distinct quorum sensing response dynamics. Data are representative of 2 biological replicates. We could not observe an effect on P*wgeA*, probably due to substantial loss of AHLs during harvest and sterile filtration of the supernatants. **c** Model illustrating frequency modulation of the *S. meliloti* quorum sensing response.

The differences in *sinI* expression pulse frequency in single cells should in turn impact behaviour on the level of the group, as they will affect the overall AHL production rate of the population, and, consequently, the velocity with which autoinducer concentrations in the environment reach the threshold to trigger the quorum-sensing response. To test this rationale, we followed the wild-type colonies that had shown frequency-modulated pulsing in *sinI* expression due to growth conditions or c-di-GMP levels (Fig. 3d) for 10 or more hours after they became three-dimensional. At this stage we assessed fluorescence no longer at the single-cell level, but as mean fluorescence intensities of the whole colony from a *wgeA* promoter-*mCerulean* fusion—the *wgeA* promoter regulates expression of a gene cluster involved in production of galactoglucan, an exopolysaccharide (EPS) that plays an important role in *S. meliloti* colony expansion and sliding motility^39–41^ and that is a central part of the organism’s quorum sensing response^28,42^. As expected, response onset in strains displaying higher *sinI* expression pulse frequencies could be observed several hours earlier and at smaller colony sizes (Fig. 4a, Supplementary Fig. 12a–f) than in colonies with lower pulse frequencies.

However, as phosphate starvation and c-di-GMP levels might impact EPS production not only via quorum sensing (e.g., Supplementary Fig. 12c), we furthermore sought to isolate AHL production-response-dynamics from other regulatory networks in the organism. To this end, we made use of the constructs with artificially altered *sinR* expression levels and *sinI* expression pulse frequencies (Fig. 2d, Supplementary Fig. 5), harvested their supernatants at various optical densities and added them to the growth medium of an *S. meliloti* AHL indicator strain, assuming that differences in the quorum-sensing response of the latter should then solely result from differences in the amount of AHLs in the respective supernatants (Supplementary Fig. 12g, h). Even in this decoupled system, response curves staggered according to pulse frequencies of the donor strains (Fig. 4b). Thus, when adapting to changes in environment or lifestyle, *S. meliloti* cells adjust AHL synthase gene expression pulse frequency, resulting in response onset at larger or smaller cell numbers (Fig. 4c).

## Discussion

The stochastic pulsing in *sinI* expression reported here resembles the pulsatile activity of several stress-responsive transcription factors in *Saccharomyces cerevisiae*^43–45^, σ^B^ and other alternative sigma factors in *Bacillus subtilis*^32,46^, and the short gene expression bursts from the uninduced *lac* promoter in *E. coli*^47,48^. Similar activity profiles have been furthermore described for higher eukaryotes including mammals, and the terms ‘transcription pulse’ and ‘transcriptional burst’ are sometimes used synonymously to describe such phenomena^49,50^. In contrast, Levine et al. define pulsing as a phenomenon “generated by genetic circuits that activate and deactivate key regulators and modulate pulse characteristics, such as frequencies and amplitudes”, whereas “transcriptional bursting […] results from the stochastic nature of gene expression”^51^. However, judging from our findings on *sinI* expression, the genetic circuit and the stochastic nature of gene expression are not always clearly distinguishable; rather, stochasticity is an integral part of the *S. meliloti* Sin system.

For instance, when comparing *sinI* expression pulsing and the pulsatile activity of the *B. subtilis* stress response sigma factor σ^B^ ^32^, both are asynchronous and share features like variability in amplitude and frequency modulation by physiological factors. On the other hand, they differ fundamentally with respect to the stochasticity involved, indicative of the disparate mechanisms underlying the two phenomena: Whereas *S. meliloti* cells carrying two different *sinI* promoter-fluorophore gene fusions displayed highly diverging intensities from the two reporters both from cell to cell and within individual cells (Fig. 1b, Supplementary Fig. 2), fluorescence intensities of analogous *sigB* promoter-fluorophore fusions in *B. subtilis* only varied from cell to cell, but were highly correlated within cells^32^. Moreover, activity of the *sigB* promoter was also highly correlated with activities of other σ^B^-regulated promoters in the respective cells^32^. Stochasticity in σ^B^ activity is thus restricted to whether or not, in a given cell at a given time, a pulse is initiated. This decision according to Locke et al. is triggered by a phosphoswitch, i.e., fluctuations in the ratio of phosphatases and kinases acting on the σ^B^ anti-anti-sigma factor that set off time-delayed positive and negative feedback loops, with the positive feedback first turning stochastic *sigB* promoter activation into a cell-wide pulse, and the negative feedback subsequently terminating it^32^.

In contrast, *sinI* expression pulses begin and end without feedback loops. Instead, they very simply stem from instability and scarcity of the key activator SinR (Figs. 2 and 3e), and very likely also from low binding affinity of SinR to the *sinI* promoter (Fig. 3c). Together, these biochemical properties of SinR yield a very low probability for a *sinI* transcription event, and a short duration of such an event if it does occur. With respect to the underlying mechanism, pulsing in *sinI* expression thus is a reversed image of stochastic gene expression from uninduced *lac* promoters in *E. coli*: For a reduced version of the promoter comprising only the *O*_*1*_ and *O*_*3*_ operators, Yu et al. reported short transcriptional bursts with a mean frequency of 1.2 events per cell cycle^47^, and Cai et al. reported similarly brief bursts with a mean frequency of 0.11 events per cell cycle for the wild-type *lac* promoter comprising all three operators *O*_*1*_, *O*_*2*_ and *O*_*3*_^48^. In both cases, the transcriptional bursts were attributed to stochastic and brief dissociation of the *lac* repressor LacI from the respective promoters. Furthermore, both cell-to-cell and within-cell heterogeneity was observed in a seminal study by Elowitz et al. from two promoter-fluorophore gene fusions in which the identical synthetic promoters contained the *O*_*1*_ operator^30^. However, stochasticity in this case was much less prominent than observed for the two analogous *sinI* promoter-fluorophore gene fusions (Fig. 1b, Supplementary Fig. 2), probably because the synthetic promoter^52^ does not enable the DNA loop formation crucial for enhanced repression by LacI^53^, thus making dissociation events more likely. Based on differences in repression of the *lac* promoter versions^53^, and on differences in burst frequencies^47,48^, one would therefore expect stochasticity to be more prominent for the *O*_*1*_ & *O*_*3*_ *lac* promoter version used by Yu et al., and even more so for the wild-type version comprising all three operators studied by Cai et al.

Due to the very low probability for SinR binding to the *sinI* promoter, it is impossible to predict whether or not a given cell at a given time will experience a *sinI* expression pulse. Nevertheless, *sinI* expression is by no means random or arbitrary in the sense of ‘happening without cause or reason’—over a large enough population, the fraction of cells with a SinR-*sinI* promoter complex and ensuing *sinI* expression is clearly defined by abundance of SinR, and by abundance of ExpR and AHLs affecting SinR binding affinity (Fig. 3). Similarly, the term ‘noise’, albeit widely used as a synonym for stochasticity^31,54,55^, does not seem appropriate in this context, since it has connotations of mere statistical fluctuations. In contrast, the Sin system is based on low probabilities, and without them, regulation of *S. meliloti* quorum sensing would be entirely different: A higher binding affinity of SinR to its promoter, for instance, with everything else unchanged, would considerably increase *sinI* expression rate, and the same is of course true for higher SinR abundance (Supplementary Fig. 5d); both would thus strongly increase AHL production in the population and accelerate quorum-sensing dynamics (Fig. 4b). If, on the contrary, the dynamics were to be preserved, a steady *sinI* transcription would have to be compensated for by, e.g., a reduced *sinI* translation rate, a reduced AHL production rate, and/or a reduced sensitivity of the AHL receptor to autoinducers. The Sin system thus represents a probabilistic switch operating at low odds, and the setup of this switch furthermore allows for the integration of physiological factors, as these either fine-tune abundance of SinR (Fig. 3a, b), or its binding affinity (Fig. 3c), and thereby modulate *sinI* expression pulse frequency.

The connection between environmental cues and quorum sensing dynamics per se is not novel: Population-level studies in *S. meliloti* had already shown *sinI* expression to be affected by the respective cues^29,36^, just as—for instance—population-level studies in *A. fischeri* had shown luciferase production and bioluminescence to be delayed via catabolite repression^16–20^. Indeed, Fuqua et al. emphasized the role of physiological factors when first proposing the term ‘quorum sensing’, stating that, in addition to the sufficiently high cell density for autoinducers to accumulate to a threshold concentration, “first, some external environmental signal other than an autoinducer must be perceived”^2^. Dunn and Stabb reasoned that “by embedding quorum signalling with […] regulatory systems [like catabolite repression], bacteria are able to modulate the production of autoinducers such that their concentration reflects not only cell density but also specific parameters of their environment”, and that target genes are thus regulated “not always with direct correlation to population numbers”^3^. And after examining the activity of *P. aeruginosa las* and *rhl* quorum sensing systems under 46 growth conditions, Duan and Surette even concluded that “no correlation could be established between cell densities and the activation of quorum sensing expression […], indicating the absence of a specific cell density as a prerequisite for quorum sensing activation”^22^.

Similarly, we found that the onset of the quorum-sensing response in *S. meliloti* populations is triggered at smaller or larger cell numbers depending on the physiological state of the individual cells (Fig. 4a, Supplementary Fig. 12), implying that the process of autoinducer production and sensing in *S. meliloti* is likewise not a simple matter of counting cell numbers as suggested by the analogy of the quorum. Since we furthermore found that the physiological state of the individual cells is encoded in their *sinI* expression pulse frequency (Fig. 3), the process seems more comparable to a voting in a local community, or to the collective decision-making described for social insects, e.g., during selection of a new nest site by a swarm of honey bees^56–60^: Whereas the vigour of a scout bee’s waggle dance is proportional to the quality of the potential nest site it has explored, convincing more bees to likewise visit that site and cast their votes, the pulse frequency with which an individual *S. meliloti* cell expresses the AHL synthase gene carries information about its physiological state and need for behavioural adaptation. Even a similar amplification process appears to be involved, as the AHLs produced by one bacterium facilitate *sinI* expression in its neighbours by increasing binding affinity of SinR to the *sinI* promoter, should the neighbours experience a similar need for action and, thus, a similar increase in SinR abundance. Due to the common pool of autoinducers—comparable to a ballot box—the pulse frequencies of all members of the population are then integrated into the total AHL concentration; only if this vote crosses the threshold, the response behaviour is initiated.

As mentioned above, phenotypic heterogeneity has been reported not only for AHL synthase gene expression in *S. meliloti*^8^, but also for expression of the homologous genes *ngrI* and *traI* in its close relative *S. fredii*^7^, the homologue *ahlI* of *P. syringae*^6^, and for expression of the *agr* operon encoding the quorum sensing system of *L. monocytogenes*^5^; in these studies, the respective quorum sensing-ON and -OFF fractions determined by microscopy snapshots or flow cytometry were also affected by environmental factors^5–7^. It would be curious to see whether these heterogeneities represent stable subpopulations, or likewise result from asynchronous stochastic pulsing, thus making frequency modulation as described here a recurring mode for collective decision-making in bacterial quorum sensing.

## Methods

### Media and growth conditions

Rich media were used for strain construction and maintenance: lysogeny broth (LB) medium (10 g/l tryptone, 5 g/l yeast extract, 5 g/l NaCl) for *Escherichia coli* strains, tryptone-yeast extract (TY) medium (5 g/l tryptone, 3 g/l yeast extract, 0.4 g/l CaCl_2_ x 2 H_2_O) for *Sinorhizobium meliloti* strains.

If required for selection during *E. coli* strain construction or for plasmid maintenance in *E. coli* strains, kanamycin was added at 50 mg/l, gentamicin at 8 mg/l and ampicillin at 150 mg/l to solid media. For selection during *S. meliloti* strain construction and for plasmid maintenance in *S. meliloti*, streptomycin was added at 600 mg/l to solid media, kanamycin at 200 mg/l, and gentamicin at 30 mg/l. For liquid cultures, antibiotic concentrations were generally reduced by half if not indicated otherwise. Selection for sucrose sensitivity of *S. meliloti* clones after double homologous recombination was carried out on LB agar containing 10% (w/v) sucrose^61^.

Starter cultures for (time-lapse) fluorescence microscopy were grown overnight in 3 ml TY medium to stationary phase; the rationale for beginning microscopy experiments with stationary phase cells was that—despite potentially different quorum-sensing response dynamics—this way all wild-type (*expR*^*+*^) strains should have reached the same stage of the quorum sensing process, i.e., the ExpR-AHL-induced negative feedback on *sinR* expression at very high AHL concentrations^28,29^.

Starter cultures for flow cytometry, microplate reader measurements, single-molecule microscopy and western blot analysis of Flag-tagged SinR were grown in 3 ml modified morpholinopropane sulfonate (MOPS)-buffered medium slightly adapted from ref. ^62^ to exponential phase; the exact composition was 1x MOPS solution (10 g/l MOPS, 10 g/l mannitol, 3.93 g/l sodium glutamate, 0.246 g/l MgSO_4_ x 7 H_2_O, pH 7.2, autoclaved), with CaCl_2_ (37 mg/ml, autoclaved), FeCl_3_ x 6 H_2_O (10 mg/ml, filter-sterilized and stored at 4 °C), oligo-elements (3 mg/ml H_3_BO_3_, 2.23 mg/ml MnSO_4_ x 4 H_2_O, 0.288 mg/ml ZnSO_4_ x 7 H_2_O, 0.125 mg/ml CuSO_4_ x 5 H_2_O, 0.065 mg/ml CoCl_2_ x 6 H_2_O, 0.12 mg/ml NaMoO_4_ x 2 H_2_O, filter-sterilized) and biotin (1 mg/ml, filter-sterilized and stored at 4 °C) all added in a 1:1000 dilution, and KH_2_PO_4_ (174 mg/ml, autoclaved) added in a 1:500 dilution. For experiments involving titration of *sinR* expression levels, all starter cultures were grown in presence of gentamicin for plasmid maintenance. For main cultures, if not otherwise indicated, 3 ml fresh modified MOPS-buffered medium without antibiotics was inoculated from starter cultures to yield an OD_600_ of about 0.1–0.3 at harvest the next morning; when involving titration of *sinR* expression levels, isopropyl-β-D-thiogalactopyranoside (IPTG) was added at 0.5 mM. For phosphate starvation, overnight cultures were harvested, washed three times in MOPS-buffered medium without phosphate, resuspended in MOPS-buffered medium without phosphate, and incubated for 5 more hours.

*E. coli* strains were grown at 37 °C, *S. meliloti* strains at 30 °C. Conjugations were incubated at 30 °C. Liquid cultures were grown in glass test tubes shaking at 200 rpm.

### Strain construction

Cloning was performed in *E. coli* DH5α, and final constructs were verified by DNA sequencing. Plasmid transfer into *S. meliloti* was carried out by *E. coli* S17-1-mediated conjugation, and if integration into the *S. meliloti* genome via single or double homologous recombination was involved, the resulting strains were again verified by sequencing. Strains, plasmids and primers used are listed in Supplementary Tables 2–4. Details on strain constructions are given in the Supplementary Methods 1.

### (Time-lapse) fluorescence microscopy

Starter cultures were prepared as described above. One millilitre of starter culture was harvested by centrifugation (4000 × *g*, 5 min, RT), and cells were either immediately resuspended in modified MOPS-buffered medium to an OD_600_ of 0.25, or first washed three times in 1 ml MOPS-buffered medium without phosphate (for phosphate starvation conditions, to remove residual phosphate) or in 1 ml MOPS-buffered medium containing 2 mM phosphate (for the corresponding rich growth condition; and for *expR*^*+*^ strains, to remove accumulated AHLs). Cell density was adjusted via serial dilutions to an OD_600_ of 0.000025.

Two to three hours before harvest, agarose pads made from modified MOPS-buffered medium containing 1.5% (w/v) molecular biology grade agarose (Eurogentec) were cast either in 17 × 28 mm or 9 × 9 mm Frame Seal in situ polymerase chain reaction and hybridization slide chambers (Biorad); the smaller frame size was chosen for side-by-side comparison of phosphate starvation *vs*. rich growth conditions as these required different pad composition, and for comparison of wt, *dgc0* and *pde0* strains to avoid alteration of strain-specific quorum sensing response dynamics by diffusing AHLs. Prior to adding cells, pads were allowed to dry for 8–12 min depending on temperature and air flow; then, 0.3 µl per cell suspension (OD_600_ of 0.000025) were spotted on the pads, yielding ~3–4 dozen single cells per spot. For the phosphate starvation condition and the corresponding control condition, three additional 0.3 µl spots of scavenger/indicator cells (OD_600_ of 0.01) were added at the far side of the pads to speed up consumption of residual phosphate and onset of discernible phosphate starvation. To detect the latter, scavenger/indicator cells carried the *pstS* promoter-*mVenus* fusion; to exclude direct effects on the read-out, they also carried a *sinI* deletion and were thus incapable of producing AHLs.

Microscopy was performed with an Eclipse Ti-E inverse research microscope (Nikon) with automated stage and shutters and a Plan Apo λ 100x/1.45 oil objective (Nikon) in an incubation chamber set to 30 °C. For snapshots, pads were incubated 15–17 h before imaging; for time-lapse fluorescence microscopy, pads were immediately searched for individual cells. Coordinates of the cells were recorded, and phase contrast and fluorescence images of the growing colonies were automatically taken every 20 min using the NIS Elements Advanced Research software version 4.13 (Nikon) and an iXon3 electron-multiplying charge-coupled device (EMCCD) camera (Andor, Oxford Instruments) over a period of at least 15 h. Subsequently, *expR*^+^ strains were followed for at least 10 more hours using the 2 × 2 Large Image function of the NIS Elements ND Acquisition module, as colonies then grew larger than the field of vision of the camera. In this case, stitching of the image stacks was performed immediately on phase-contrast images (15% overlap) using the NIS Elements software. Over the whole time-lapse experiment, focus was maintained using the Perfect Focus System (PFS). Furthermore, to facilitate focus maintenance, microscope and incubation chamber were preheated for at least 4–5 h, preferably even overnight.

Fluorophores were excited with lasers: mCerulean with a 445 nm CUBE Laser (Coherent Inc., USA) [excitation band pass (ex bp) 445/30, beamsplitter (bs) 458, emission band pass (em bp) 483/32], mVenus with a 514 nm OBIS Laser (Coherent Inc., USA) (ex bp 500/24 nm, bs 520 nm, em bp 542/27 nm) and mCherry with a 561 nm Sapphire Laser (Coherent Inc., USA) (ex bp 562/40 nm, bs 593 nm, em bp 624/40 nm). Laser intensities, exposure times and EM gains were applied as follows: 3%, 600 ms, 100 for P*sinI-mVenus*; 5%, 600 ms, 100 for P*trp-mCherry*; 8%, 1 s, 100 for P*sinI-mCerulean*; 5%, 1 s, 100 for P*sinI-mCherry*; 4%, 1 s, 100 for P*lac-mVenus*; 25%, 1 s, 100 for P*sinR-mCherry*. For the 2 × 2 images, settings were modified as follows: 0.5%, 2 × 2 binning, 1 s, 100 for P*sinI-mVenus*; 0.5% 2 × 2 binning, 600 ms, 150 for P*wgeA-mCerulean*. Conversion gain was always set to 1. Generally, excitation intensities and exposure times were chosen as low as possible to minimize phototoxicity.

### Processing, segmentation, tracking and single-cell analysis of early (2D) time-lapse data

The NIS Elements software was used to crop image stacks to the maximum spatial extent of the colony and to the time period during which cells were growing in a single layer. Further processing was done with a combination of Schnitzcells version 1.1^63^, Ilastik version 1.3.3post3^64^, and a custom-built Matlab (MathWorks, Natick, Massachusetts) programme^65^. The workflow closely follows the pipeline developed by van Vliet et al.^65^, with the exception that segmentation was performed using Ilastik instead of Schnitzcells.

Segmentation was done either on the RFP (for P*trp-mCherry*) or the YFP (for P*lac-mVenus*) channel using the Ilastik pixel classification workflow^64^. Before import into Ilastik, fluorescent images were deconvolved applying the Lucy-Richardson method (as implemented in the Matlab ‘deconvlucy’ function) using the experimentally determined Point Spread Function (PSF) of the microscope. Pixels were then classified into two classes (‘background’ and ‘cells’), and the resulting probability images were imported into Matlab for post-processing. The cell class probabilities were smoothed using a Gaussian kernel (with a size of 1 pixel) and thresholded using a fixed threshold value of 0.6 to obtain putative cell masks. Subsequently, a binary closure operation was performed to remove internal holes in the cell masks, and a morphological opening operation (erosion followed by dilation) to separate adjacent cells. The morphological opening was done in two passes: First, all cell masks were opened by 1 pixel; subsequently, any remaining objects that exceeded the expected cell width were automatically classified as potential cell clusters and a second opening by 2 pixels was applied to separate cells in these clusters. The resulting cell segmentation masks were then manually corrected using the Schnitzcells graphical user interface (GUI).

Cell tracking was performed with the automated tracking routine of Schnitzcells 1.1 (original version)^63^. Subsequently, all tracking results were manually checked and corrected using the Schnitzcells GUI.

Cell features (length, growth rate, and mean fluorescence intensity as a proxy for gene expression level etc.) were extracted using a custom-written Matlab programme which had been previously developed for *E. coli* microcolonies^65^ and which was here adapted for *S. meliloti*. We summarize the most important details below.

Cell lengths were estimated using the method developed by Kiviet et al.^66^: Here, a third-degree polynomial, *f*(*x*), is fitted to the cell mask. This polynomial is extrapolated by 10 pixels in both directions and the locations of the cell poles are determined automatically by calculating the silhouette proximity (sum of the squared distances to closest 25 pixels in cell mask) along the centerline. This measure increases sharply at the cell poles, and the location of the poles can thus be taken as the points where the silhouette proximity reaches 110% of the average value in the cell centre. Subsequently, the cell length is calculated as $L={\int }_{{x}_{0}}^{{x}_{1}}\scriptstyle\sqrt{1+{{f}^{{\prime} }(x)}^{2}}{dx}$, where *f* ′(*x*) is the derivative of *f*(*x*) and *x*_0_ and *x*_1_ are the positions of the cell pole (*x* is the coordinate along the cell-centerline). In addition, we estimated cell lengths using the length of the major axis of an ellipse fitted to the cell masks (calculated using the Matlab ‘regionprops’ function). Overall, the two methods agree well, however, the first (based on polynomial fitting) is more robust to curved cells and it was therefore used for all data shown in the figures.

Cell growth rates, *r*, were calculated by fitting an exponential curve to time-trajectories of the measured cell length over time: $L\left(t\right)=L(0)\cdot {e}^{r\cdot t}$. To estimate the growth rate directly before and after cell division, we first extended cell length measurement across divisions by summing up the cell lengths of the two daughter cells (extension after cell divides) and by taking a fraction of ${L}_{0}/({L}_{0}+{L}_{0,{{{{{{\rm{sister}}}}}}}})$ of the mother cell length, where *L*_0_ and *L*_0,sister_ are the lengths of a cell and its sister at their birth (extension before cell is born)^65,66^. We then performed a linear regression on the log-transformed cell lengths over a sliding window of 11 time points (200 min) to obtain an estimate of the growth rate.

To accurately estimate expression levels of genes of interest, the respective fluorescence images were corrected for imaging artefacts, following the procedure described in van Vliet et al.^65^: First, we performed a shading correction to correct for inhomogeneities in the light field by dividing each pixel in the fluorescence images by the corresponding pixel in the shading images (an image obtained from a homogenous fluorescent sample, normalized to an average intensity of 1). Second, we corrected for diffraction artefacts by performing a deconvolution using the Lucy-Richardson method (as implemented in the Matlab ‘deconvlucy’ function) applying the experimentally determined PSF of the microscope. Third, we performed a background correction by subtracting the median intensity over all background pixels (i.e., all pixels that are not part of any segmented cell). Finally, we corrected for segmentation inaccuracies by only estimating the mean fluorescence intensity within the centre of the cell mask. To do so we first eroded the cell mask with 5% of the cell width; subsequently, we calculated the mean fluorescence intensity, *M*, over all pixels within this eroded cell mask.

The change in gene expression level over time—which we call ‘expression rate’ in short—was calculated like the promoter activity *P* in Locke et al.^32^. There the authors define this rate per unit length $\widetilde{P}$ as: $\widetilde{P}=\frac{P}{L}={rM}+\gamma M+\frac{{dM}}{{dt}}$. In this equation, *L* is the length, *r* the elongation rate, and *M* the mean fluorescence intensity of the cell, all calculated as described above. $\frac{{dM}}{{dt}}$ is the change in mean fluorescence intensity over time and was estimated as the coefficient of a linear regression calculated over a sliding window of 11 time points (200 min). Before performing the regression, we extended cell measurements across division events by adding the mean values of the intensity in the two daughter cells for time points after cell division and by adding the intensity in the mother cell for time points before cell birth. The final constant in the equation above, *γ*, is the degradation rate of the fluorescent protein. We estimated its value by manually selecting 51 cells in which there was no discernable gene expression rate (i.e., $P=\widetilde{P}=0$). From the equation above it follows that when $\widetilde{P}=0$ we can estimate the degradation rate as: $\gamma =-r-\frac{d[{{\log }}M]}{{dt}}$, where the elongation rate, *r*, is measured as described above, and where $\frac{d[{{\log }}M]}{{dt}}$ is estimated as the coefficient of a linear regression of log *M vs*. time; the regression was again calculated over a sliding window of 11 time points, and in doing so log *M* values were again extended across cell division using the respective values from mother and daughter cells. We thus obtained an estimated value of *γ* = 0.0015 1/min.

We defined pulses as a transient increase in $\widetilde{P}$. Since a pulse can last longer than a cell life time, or begin in a mother cell and continue in one or both of its daughters, we needed a method that is not affected by cell division events to detect them. To this end we first traced all cell lineages backward in time; for each cell present in the last frame of the image stack we thereby obtained an extended linage that starts at frame 1 with a founder cell and ends at the last frame with the focal cell itself. It is important to note that these lineages are not statistically independent—cells that occur early in a colony are of course part of multiple lineages; however, we correct for this at a later stage by removing all multiple detections.

For each lineage we then used a peak finding algorithm (implemented in the Matlab function ‘peakfinder’) to find all candidate pulses. As this ‘peakfinder’ function considers symmetric prominence—i.e., both increase and decrease—, we subsequently calculated for each candidate pulse the prominence backward in time: This corresponds to an increase in the gene expression rate relative to the lowest value obtained since the last pulse, or since the beginning of the movie, whichever comes first. Only pulses with a prominence backward in time of more than 6 1/min were maintained; this threshold value had been determined based on visual inspection of a large number of trajectories of the strain with the lowest pulse frequency (the *sinR* promoter mutant), and the same threshold was used for all strains and conditions. Finally, we removed all duplicate detections and characterized each pulse by its prominence (backward in time, i.e., the increase), its absolute height, and the time since the last pulse.

The average pulsing frequency per unit time was calculated for each colony as: $f_{{{{{{{\rm{pulse}}}}}}}}=\frac{{N}_{{{{{{{\rm{pulse}}}}}}}}}{{dt}\,\cdot\, {\sum }_{i=1}^{T}{n}_{i}}$, where *N*_pulse_ is the total number of pulses that occurred in the colony, *dt* is the time interval between frames, *n*_*i*_ is the number of cells present at frame *i*, and the sum is over all *T* frames in the movie. The denominator measures the total observation time, taking into account that the number of cells increases over the duration of the movie.

### Processing, segmentation and per-colony analysis of late (3D) time-lapse data

Image stacks were cropped using the above-mentioned NIS Elements software version 4.13. Image analysis was performed as previously described^67^, using the General Analysis module of the NIS Elements Advanced Research software version 4.5: Binary layers were constructed along colony perimeters on phase-contrast images. Based on binary layers, colony area and mean fluorescence intensity per colony were determined, i.e., the ratio of total fluorescence intensity per colony area. From these mean fluorescence values, background fluorescence intensities were subtracted.

### Flow cytometry and flow cytometry data analysis

Starter cultures were prepared as described above. One millilitre of final cultures was harvested by centrifugation (4000 × *g*, 5 min, 4 °C), resuspended in an equal volume of ice-cold phosphate-buffered saline (PBS; 8 g/l NaCl, 0.2 g/l KCl, 1.44 g/l Na_2_HPO_4_, 0.24 g/l KH_2_PO_4_, pH 7.2), diluted to a final OD_600_ of 0.0125 in ice-cold PBS and kept on ice until analysis.

Fluorescence-activated cell analysis was carried out with a BD LSRFortessa SORP flow cytometer (BD Biosciences, Germany). mNeonGreen intensity was assessed employing a 488 nm laser [band pass filter (bp) 510/20 nm], mVenus intensity employing a 514 nm laser (bp 542/27 nm), and mScarlet-I intensity employing a 561 nm laser (bp 586/15 nm) lasers.

Flow cytometry data were collected with BD FACSDiva 8.0.1 (BD) in FCS 3.0 file format, and data analysis was carried out with FlowJo 10.6.0 software (BD). Gating (Supplementary Fig. 13) was first performed on forward and side scatters (FSC and SSC, respectively) to remove dead cells and debris (SSC-A over FSC-A) and to exclude doublets (SSC-W over SSC-H). Subsequently, using the FlowJo Exchange DownSample plugin, the number of events per sample was reduced to 15,000 to ensure equal sample size. Strains lacking the *sinI* promoter-fluorophore gene fusion(s) with otherwise identical genetic backgrounds served as negative controls. Cells in the read-out samples with higher fluorescence intensities than those of the respective control cells were assessed as ‘positive’. The fraction of cells per sample assessed as ‘positive’ and their corresponding median fluorescence values were likewise determined with FlowJo.

### Microplate reader fluorescence and optical density measurements

To assess the effect of different *sinI* expression pulse frequencies on quorum sensing response dynamics, for each of the five strains with different *sinR* expression levels (analogous to the strains used for Fig. 2d, but without the fluorophore gene fusion) five test tubes with modified MOPS-buffered medium containing 0.5 mM IPTG were inoculated to five different OD_600_ and grown overnight. The next morning, 2 ml of each culture were harvested and cells pelleted by centrifugation (4000 × *g*, 5 min, RT). Supernatants were transferred to fresh tubes, sterile-filtered, and 500 µl of sterile supernatants mixed with 500 µl of indicator strain culture adjusted to an OD_600_ of 0.375. Of each of the 25 supernatant-indicator strain suspensions, 3 × 100 µl were distributed in a 96-well microtiter plate as technical replicates. Further wells were filled with 3 × 100 µl of indicator strain mixed 1:2 with fresh medium, and with medium only as sterile/blank control. Plates were covered and incubated for 12 hours in an Infinite M Plex microplate reader (Tecan) set to 30 °C and shaking at 200 rpm. Every 30 min, mVenus intensity and OD_600_ were measured using i-control 1.8 SP1 (Lifescience Tecan).

To assess an effect of different *sinR* expression levels on growth, starter cultures of the respective strains were diluted to an OD_600_ of 0.15 in modified MOPS-buffered medium containing 0.5 mM IPTG, and 6 × 100 µl per strain were distributed in a 96-well microtiter plate as technical replicates; further wells were filled with medium only as sterile/blank control. Plates were covered and incubated for 20 h in the same Infinite M Plex microplate reader set to 30 °C and shaking at 200 rpm, and OD_600_ was measured every 30 min using i-control 1.8 SP1 (Lifescience Tecan).

### Single-molecule microscopy, image processing and analysis

Starter cultures were prepared as described above. Final cultures were harvested by centrifugation (4000 × *g*, 5 min, RT) and washed twice with 1 ml modified MOPS-buffered medium. Formaldehyde was then added to a final concentration of 3.7% (v/v), mixed gently by inversion and incubated for 15–20 min. After fixation, cells were washed twice with 1 ml EZ rich defined medium (EZRDM; Teknova, USA) and finally resuspended in 1 ml EZRDM.

For agarose pads, 1% (w/v) low melting agarose (Merck/Sigma-Aldrich, Germany) in EZRDM was incubated at 70 °C for 12 min to melt the agarose and then cooled down to 37 °C. The agarose solution at 37 °C was placed on indented microscope slides (Thermo Fisher, Germany), sealed with coverslips that had been cleaned overnight in 1 M KOH (Merck/Sigma-Aldrich), and allowed to set for 2 h.

Cells were then placed on the pads and imaged on a custom-built setup based on a Nikon Ti Eclipse microscope equipped with a set of dichroic mirrors and filters (ET dapi/Fitc/cy3 dichroic, ZT405/488/561rpc rejection filter, ET525/50 or ET610/75 band pass, all AHF Analysentechnik, Germany), and a CFI Apo TIRF 100x/1.49 oil objective (Nikon). A 561 nm OBIS laser (Coherent Inc., USA) was controlled via an acousto-optical tunable filter (AOTF; Gooch and Housego, USA). Laser intensity was set to 100 W/cm² and each field of view was imaged for 6 s with 60 ms frame time (100 frames) using Micro-manager 1.4 (https://micro-manager.org/Citing_Micro-Manager) to record a single-molecule movie. Furthermore, for each field of view a bright light snapshot was recorded to manually determine the number of cells.

For data analysis, each single-molecule movie was flattened to a single frame where each pixel was averaged over the entire movie. The resulting image was post-processed with the ThunderSTORM ImageJ plugin (https://github.com/zitmen/thunderstorm) to count fluorescent spots. Here, an intensity threshold of 20 photons (based on the negative control strain lacking *mScarlet-I*) was used to avoid false-positive spots, and results were furthermore filtered in order to discard events outside of cells.

### In vivo protein stability assay

Starter cultures were prepared as described above. Prior to the experiment, 9 × 15 ml of 150 ml overnight culture were distributed in 100 ml Erlenmeyer flasks equilibrated to 30 °C and further incubated for 15 min at 30 °C shaking at 200 rpm. Chloramphenicol was added to the flasks at 20 µg/ml^68^ in 1-min intervals; after addition of chloramphenicol to the last flask, all flasks were shaken for another minute to ensure homogeneous distribution and uptake of the antibiotic even in the last sample. At harvest, all flasks were put on ice, 10 ml per sample transferred to pre-cooled centrifuge tubes, and cells pelleted by centrifugation (10,000 × *g*, 5 min, 4 °C). Most of the supernatant was decanted, cells resuspended in residual medium, transferred to pre-cooled 2 ml tubes and again pelleted by centrifugation (10,000 × *g*, 5 min, 4 °C). After removal of all supernatant cells were resuspended in 2× Laemmli loading dye to a calculated OD_600_ of 20 and lysed by incubation at 95 °C for 20 min and repeated vortexing. Samples were stored at −20 °C for western blot analysis.

### Western blot analysis

For mere comparison of strain/growth condition effects via western blot analysis, cultures were prepared and harvested as above.

Five microlitres of samples were loaded on a 12.5% SDS-polyacrylamide gel, and after electrophoresis separated proteins were transferred to a polyvinylidene difluoride (PVDF) membrane (Thermo Scientific) equilibrated in transfer buffer (0.025 M Tris, 0.192 M glycine, 20% (v/v) methanol) using the semi-dry blotting procedure. The membrane was then incubated with 1× phosphate-buffered saline supplemented with Tween-20 (PBST) (8 g/l NaCl, 1.44 g/l Na_2_HPO_4_ x 2 H_2_O, 0.2 g/l KCl, 0.24 g/l KH_2_PO_4_, 1 ml/l Tween-20, pH 7.2) containing 2% (m/v) milk powder for 1 h at room temperature to block unspecific binding of antibodies.

Subsequently, the membrane was cut horizontally immediately above the 55 kDa band of the molecular weight standard, and the two parts treated separately. The upper part was incubated in 15 ml PBST with an anti-DnaK antibody raised in rabbit (Biorbyt Ltd, Cambridge) in a 1:20,000 dilution at 4 °C overnight; the lower part was incubated in 15 ml PBST containing 2% (m/v) milk powder with anti-FLAG M2-Peroxidase (horseradish peroxidase, HRP) antibody produced in mouse (Sigma-Aldrich) added in a 1:1000 dilution, likewise at 4 °C overnight. The next morning, the upper part was washed 3 times for 10–15 min with 20 ml PBST at room temperature and incubated for 1 h in 15 ml PBST with a mouse anti-rabbit IgG-HRP (Santa Cruz Biotechnology) in a 1:10,000 dilution at room temperature. Finally, both parts were washed 3 times for 10–15 min with 20 ml PBST at room temperature and developed with Pierce ECL Western Blotting Substrate (Thermo Scientific) according to manufacturer instructions. Images were taken with a ChemiDoc MP Imaging System (Biorad) and the ImageLab software version 5.2.1 set to Chemi hi sensitivity mode (4 × 4 binning and signal accumulation).

Blot images were analysed using the Gel Analysis method provided in the Fiji/ImageJ image processing software (http://imagej.nih.gov/ij). The one-phase exponential decay fits to the data were performed with Graphpad Prism software (San Diego, California).

### Protein production and purification

His_6_-ExpR was produced from pET28a-*expR*, His_6_-GB1-SinR from pEM-GB1-*sinR*, and His_6_-GB1 from the empty pEM-GB1 vector. *E. coli* BL21 (DE3) cells carrying the respective plasmid were grown at 37 °C under rigorous shaking in LB medium supplemented with 50 mg/l kanamycin (for pET28a-*expR*) or 100 mg/l ampicillin (for pEM-GB1-*sinR* or pEM-GB1). At an OD_600_ of ~0.6, the culture was shifted to 20 °C and protein production induced by addition of 1 mM IPTG. After further incubation for 20 h, cells were harvested by centrifugation (4000 × *g*, 20 min, 4 °C), resuspended in lysis buffer (20 mM of HEPES-Na pH 8.0, 20 mM KCl, 20 mM MgCl_2_, 250 mM NaCl and 40 mM imidazole) and lysed with an LM10 Microfluidizer (Microfluidics) at 12,000 psi pressure. Cell debris was removed by centrifugation (47,850 × *g*, 20 min, 4 °C), and purification was then continued at room temperature. The clear supernatant was loaded on a 1-ml HisTrap column (GE Healthcare) equilibrated with 10 column volumes (CV) lysis buffer. After washing with further 10 CV of lysis buffer, proteins were eluted with 5 CV elution buffer (lysis buffer containing 500 mM imidazole). Proteins were further purified by size-exclusion chromatography (SEC) on a HiLoad 26/600 Superdex 200 pg column (GE Healthcare) equilibrated with SEC buffer (20 mM of HEPES-Na pH 7.5, 20 mM KCl, 20 mM MgCl_2_, 200 mM NaCl). Fractions containing the desired protein were pooled, concentrated [Amicon Ultra-0.5 Centrifugal Filter Unit, 10 kDa MWCO (Millipore)], deep-frozen in liquid nitrogen and stored at −80 °C. Protein concentration was determined using a spectrophotometer (NanoDrop Lite, Thermo Scientific).

### Electrophoretic mobility shift assay (EMSA)

A 177 bp Cy3-labelled fragment of the *sinI* promoter including the ExpR and SinR binding sites was generated via PCR with primers [Cy3]264 f and 440r. DNA fragments were mixed at 2.75 nM with purified proteins in reaction buffer containing 20 mM of HEPES-Na pH 7.5, 200 mM NaCl, 50 mM KCl, 20 mM MgCl_2_, 1 µg/µl bovine serum albumin (Sigma), 0.0025 U/µl sonicated sperm DNA (GE Healthcare), 10 µM 3-oxo-C16:1-HSL (N-3-oxo-hexadec-11(Z)-enoyl-L-homoserine lactone, Cayman Chemical), and 0.1% (v/v) DMSO in a final volume of 10 µl. If included, His_6_-ExpR was added at 1 µM, His_6_-GB1-SinR and His_6_-GB1 at 70 µM. Reactions were shielded from light and incubated for 30 min at room temperature. Subsequently, 2.5 µl loading buffer [5 parts 5× Tris/Borate/EDTA (TBE) buffer (Tris 54 g/l, boric acid 27.5 g/l, EDTA 10 mM, pH 8.3) mixed with 3 parts 87% glycerol] were added, and reactions were loaded on 8% polyacrylamide gels casted with 1× TBE buffer. After electrophoresis (90 V, 2.5 h, covered from light), gels were scanned using a Typhoon imager (Typhoon Trio, Amersham Biosciences) and Typhoon Scanner Control v5.0 (GE Healthcare).

### Statistical analysis, correlations and regressions

All statistical analysis, except for determination of means and medians of fluorescence intensities measured by flow cytometry (which were calculated by FlowJo), was performed with Graphpad Prism software (San Diego, California). To assess statistical significance of single-molecule microscopy, time-lapse microscopy and flow cytometry data sets comparing two strains or growth conditions, two-tailed unpaired *t*-tests with Welch’s correction were performed, i.e., assuming that both groups of data were drawn from populations with a Gaussian distribution, but not assuming identical standard deviations for the two populations. To assess statistical significance when comparing three different strains, Welch’s ANOVA tests with a post hoc Dunnett’s T3 multiple comparisons test were performed, again assuming that all groups of data were drawn from Gaussian populations with individual variances. Statistical differences between positional subsets of data (Supplementary Fig. 9a) were assessed using a Kruskal–Wallis test, since the data even after log transformation did not follow a Gaussian distribution. Results of significance tests are always indicated as follows: ns, *P* ≥ 0.05; *, *P* < 0.05; **, *P* < 0.01; ***, *P* < 0.001; ****, *P* < 0.0001.

Correlation of P*sinI-mCerulean* & P*sinI-mCherry* data (Fig. 1b) was calculated as Spearman’s correlation (i.e., not assuming a Gaussian distribution of the respective values); *r*^2^ for pulse data vs. mScarlet-I-SinR spots (Fig. 3e) in turn was calculated from Pearson’s correlation coefficient assuming that *x* and *y* values (i.e., means of the fraction of cells with mScarlet-I-SinR spots per strain/growth condition, means of pulse frequencies, and medians of pulse amplitudes) were sampled from populations that at least approximately follow a Gaussian distribution.

### Reporting summary

Further information on research design is available in the Nature Research Reporting Summary linked to this article.

## Supplementary information

Supplementary Information

Description of Additional Supplementary Files

Supplementary Movie 1

Supplementary Movie 2

Supplementary Data_Custom code

Reporting Summary

## Data Availability

Pulse data are available in combination with the code, see below. Source data are provided with this paper.

## Source data

Source data

## Code availability

The custom code used for pulse analysis together with the respective data and instructions is available in the folder ‘Supplementary Data_Custom code’.
