# Supplementary material for: Frequency modulation of a bacterial quorum sensing response
Source: Nat Commun. 2022 May 19;13:2772. doi: 10.1038/s41467-022-30307-6 (PMC9120067; doi:10.1038/s41467-022-30307-6)
Supplement: Supplementary file 1 — Supplementary Information [file 41467_2022_30307_MOESM1_ESM.pdf]

Supplementary Information

Frequency modulation of a bacterial quorum sensing response

Vera Bettenworth, Simon van Vliet, Bartosz Turkowyd, Annika Bamberger, Heiko Wendt, Matthew McIntosh, Wieland Steinchen, Ulrike Endesfelder, Anke Becker

Contents

Supplementary Figure 1. SinR has a degenerated AHL binding site.

Supplementary Figure 2. Stochasticity in *sinI* expression.

Supplementary Figure 3. *sinI* expression rate.

Supplementary Figure 4. SinR is very unstable and scarce.

Supplementary Figure 5. SinR as a key factor.

Supplementary Figure 6. Quantification of pulse data is robust to changes in peak prominence threshold.

Supplementary Figure 7. Phosphate starvation, c-di-GMP and *expR* effects.

Supplementary Figure 8. The *expR*$^-$ *pde0* strain displays elevated c-di-GMP levels.

Supplementary Figure 9. Potential positional and temporal effects.

Supplementary Figure 10. mScarlet-I-SinR spots are homogeneous.

Supplementary Figure 11. Pulse amplitudes and fluorescence intensities.

Supplementary Figure 12. Effects on the quorum sensing response.

Supplementary Figure 13. Gating and quantification of flow cytometry data.

Supplementary Table 1. Data summary.

Supplementary Table 2. Strains.

Supplementary Table 3. Plasmids.

Supplementary Table 4. Oligonucleotides and synthetic genes.

Supplementary Methods 1. Details on strain constructions.

Supplementary Methods 2. Construction and characterization of the *expR*$^-$ *pde0* strain.

References

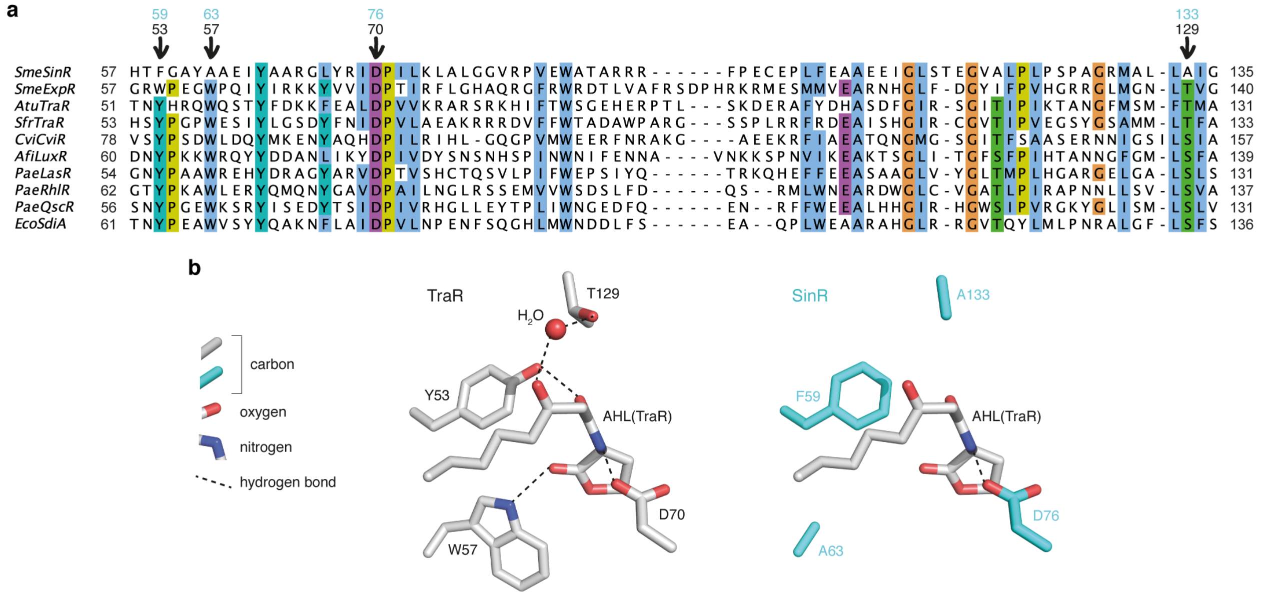

Supplementary Figure 1. SinR has a degenerated AHL binding site. a Amino acid sequence alignment of the AHL binding domains of 10 LuxR-type regulators illustrating changes of SinR in residues crucial for AHL coordination. *SmeSinR*, *Sinorhizobium meliloti* SinR (Uniprot accession number Q92PD1); *SmeExpR*, *S. meliloti* ExpR (Q2HY11); *AtuTraR*, *Agrobacterium tumefaciens* TraR (P33905); *SfrTraR*, *Sinorhizobium fredii* NGR234 TraR (P55407); *CviCviR*, *Chromobacterium violaceum* CviR (D3W065); *AfiLuxR*, *A. fischeri* LuxR (P12746); *PaeLasR*, *Pseudomonas aeruginosa* LasR (P25084); *PaeRhlR*, *P. aeruginosa* RhlR (P54292); *PaeQscR*, *P. aeruginosa* QscR (Q9RMS5); *EcoSdiA*, *Escherichia coli* SdiA (P07026). Arrows mark residues involved in coordination of the lactone ring and the 3-oxo moiety of the AHL in the crystal structure of *A. tumefaciens* TraR[1] (see **b**), numbers above indicate positions in TraR (black) and SinR (turquois) amino acid sequences. Numbers on the left and right denote the first and last residues shown in the alignment. Residues with 60% or higher conservation are indicated by boxes according to the Clustal X Colour Scheme[2]. **b** 3D structure of *A. tumefaciens* TraR (PDB entry 1L3L)[1] (left). The AHL is coordinated by hydrogen bond interactions provided by tyrosine (Y53), tryptophan (W57) and aspartate (D70). Threonine (T129) coordinates a water molecule which in turn establishes a hydrogen bond to the 3-oxo moiety of the AHL. The SinR AHL binding site (right) is modelled based on *A. tumefaciens* TraR (PDB entry 1L3L). Three of the four residues coordinating the AHL in TraR are exchanged in SinR for residues lacking the potential to establish hydrogen bonds with an AHL molecule, providing a structural basis for SinR being irresponsive to AHLs[3].

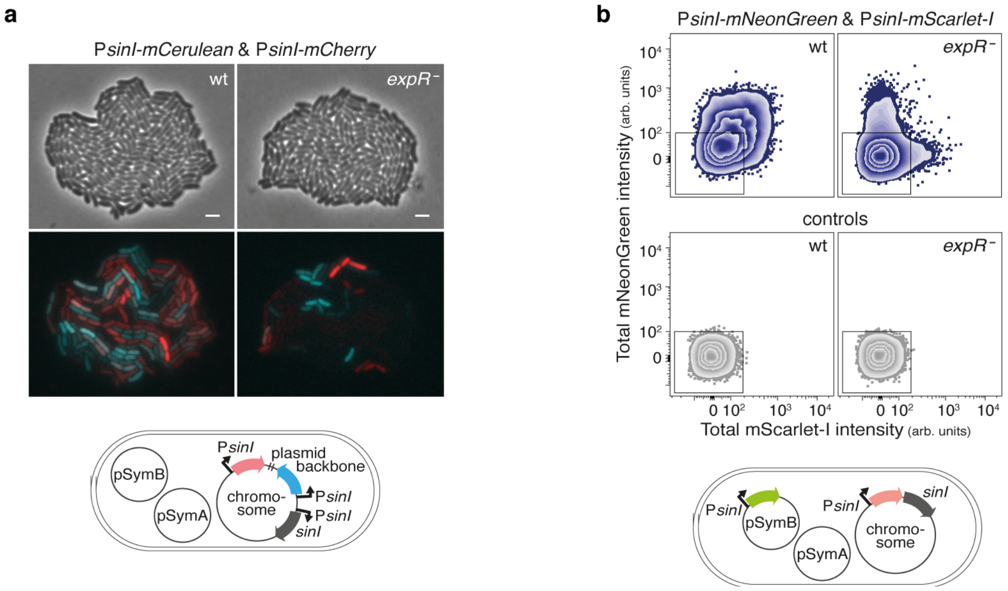

Supplementary Figure 2. Stochasticity in *sinI* expression. a Phase contrast and fluorescence microscopy snapshots (top) of wild-type (wt) and *expR*$^-$ colonies carrying two copies of the *sinI* promoter fused to *mCerulean* and *mCherry* fluorophore genes, respectively, representing exemplary raw data for Fig. 1b. In total, 10 (wt) and 12 (*expR*$^-$) colonies were imaged on 3 different days, all with similar results. Scale bars, 2 µm. Simplified sketch (bottom) of the respective promoter-fluorophore gene fusions. Both the *sinI* promoter-*mCerulean* and the *sinI* promoter-*mCherry* fusion are located on the same suicide plasmid integrated into the *S. meliloti* chromosome at the native *sinI* locus. The two promotor-fluorophore fusions are separated by approx. 4 kb of plasmid DNA. The plasmid also comprises a constitutive promoter-*mVenus* fusion for segmentation of microscopy images; the strains furthermore possess a third, dark copy of the *sinI* promoter regulating expression of *sinI*. **b** Flow cytometry analysis (top) of wt and *expR*$^-$ cells likewise carrying two *sinI* promoter-fluorophore gene fusions during exponential growth confirm strong stochasticity of *sinI* expression. Each plot indicates fluorescence intensities of 15,000 cells measured in one experiment. Blue, cells carrying the fluorophore gene fusions; grey, respective background controls. Rectangles indicate the 'negative' gate comprising at least 99.9% of background controls. Data is representative of 3 independent experiments. Simplified sketch (bottom) of the constructs employed for flow cytometry: A *sinI* promoter-*mScarlet-I* fusion is integrated into the chromosome in between the native *sinI* promoter and the *sinI* coding sequence (i.e., at the native *sinI* locus) by double homologous recombination, and a *sinI* promoter-*mNeonGreen* fusion is located on a suicide plasmid integrated into the single-copy megaplasmid pSymB in the intergenic region between the *exoP* and the *thiD* gene; pSymB is essential for *S. meliloti* viability unless crucial elements are first transferred onto the chromosome[4]. The control strains lack the fluorophore on the chromosome and carry a promoterless *mNeonGreen* on an otherwise identical suicide plasmid likewise integrated in the *exoP-thiD* intergenic region.

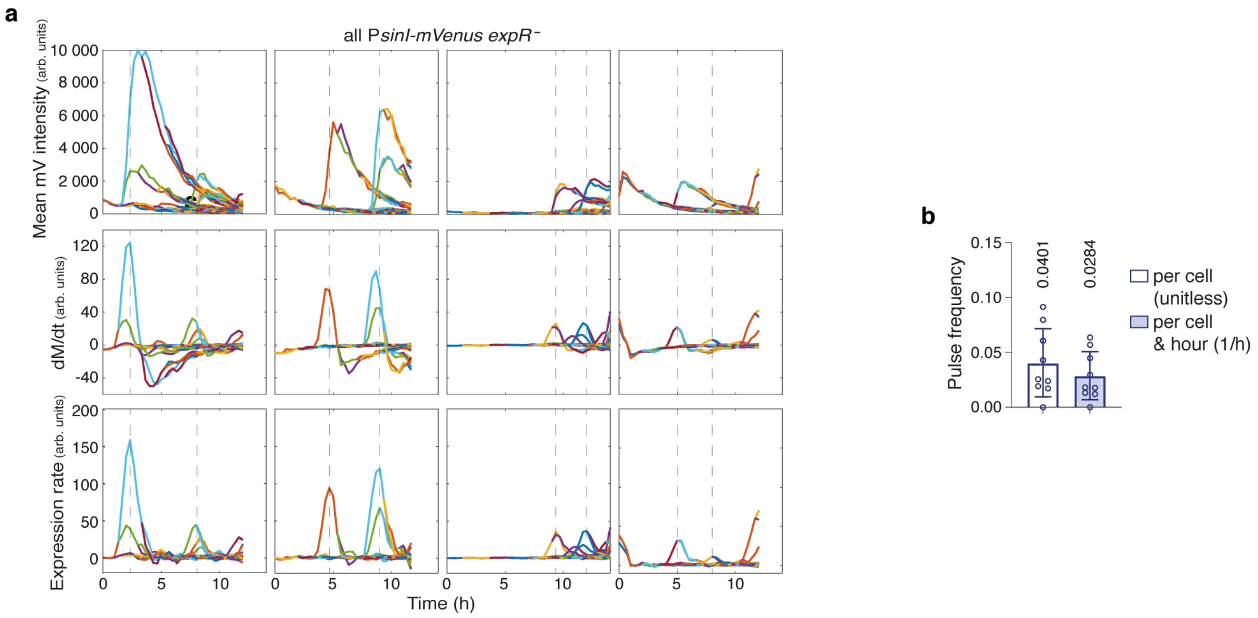

Supplementary Figure 3. *sinI* expression rate. a Plots of mean fluorescence intensities from the P*sinI-mVenus* fusion (top), the time derivatives of these mean fluorescence intensities (middle), and the corresponding *sinI* expression rates (bottom) of the *expR*⁻ microcolony from Fig. 1c over the whole experiment run time (first column) and three more *expR*⁻ microcolonies (columns two to four). Once a pulse has ended, no more fluorophores are produced, and mean fluorescence intensities slowly decline due to dilution by cell growth and/or degradation. Since the time derivatives represent the change in mean fluorescence intensities over time, they decrease as soon as fluorophore production has passed its maximum; they become negative once it completely petered out, as they then only represent the effects of fluorophore dilution and/or degradation. To deduce the actual expression rate, the time derivative is set off against a term for the dilution of fluorophores by cell growth, and a term for their – albeit small – degradation rate. In principle, the expression rate should be either 0 or above, but small negative values are possible due to inaccuracies, e.g., in single fluorescence measurements, determination of cell elongation rates (feeding into the term for fluorophore dilution), or fluorophore degradation rate. Both the time derivative and the expression rate represent fits to the data calculated over a sliding window of 11 time points; see Methods for details. In every plot, the maxima of two *sinI* expression pulses – i.e., the measurements ensuing the highest fluorophore production rate – are marked with broken lines. **b** Compilation of pulse frequencies per cell life time, and per cell life time and hour from 9 *expR*⁻ colonies imaged on 3 different days. Respective means are given and indicated by the bars, error bars indicate standard deviations, open circles indicate individual data. *N* = 2,517 cells.

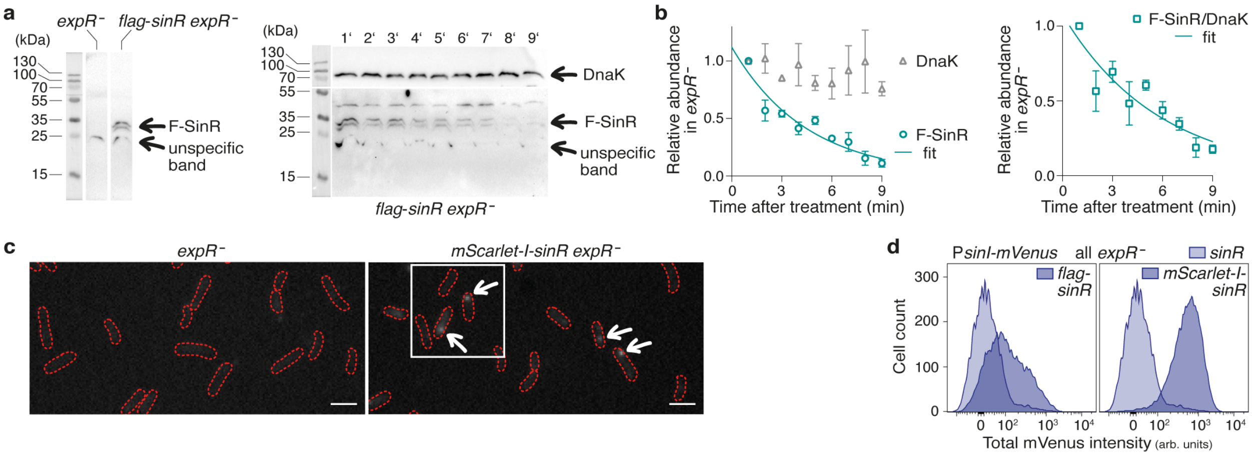

Supplementary Figure 4. SinR is very unstable and scarce. a Western blots of cell lysates identifying the Flag-tagged SinR (F-SinR) band (left) and indicating rapid F-SinR degradation after chloramphenicol treatment at 1-minute intervals (right). Numbers on the left indicate molecular weight standards. Protein stability assay representative of 3 biological replicates, see (b) for analysis. **b** Plots of relative abundance of F-SinR and DnaK (left) and F-SinR normalized to DnaK (right), each fitted with a model of one phase exponential decay. The fits were constrained with K > 0 (i.e., a degradation over time) and plateau = 0 and yield a half-life of 3.1 minutes for F-SinR [Y(0) = 1.12, R^2 = 0.87] and of 4.0 minutes for F-SinR/DnaK [Y(0) = 1.08, R^2 = 0.78]. Data for F-SinR alone is given in the main text and Fig. 2b since standard deviation in DnaK data – and thus F-SinR/DnaK data – is larger than for F-SinR data alone. Degradation of DnaK could not be fitted. Data, means ± standard deviations of 3 biological replicates. **c** Fluorescence microscopy images of cells of an *expR*⁻ control strain (left) and an *expR*⁻ strain carrying the *mScarlet-I-sinR* fusion (right). White square, area displayed in Fig. 2c (left). Scale bars, 2 µm. Images representative of 1,670 (*expR*⁻) and 2,293 (*mScarlet-I-sinR expR*⁻) cells, respectively, imaged on 3 different days (see Fig. 2c (right) for quantification). **d** Histograms of fluorescence intensities from the *sinI* promotor-*mVenus* fusion either with native *sinR* (light blue), or *flag-sinR* or *mScarlet-I-sinR* (dark blue), expressed from the native *sinR* promoter suggest that native, i.e., untagged SinR is even less stable and/or less abundant than suggested by western blot analysis and single molecule microscopy. Each histogram indicates fluorescence intensities of 15,000 cells measured in one experiment and is representative of 3 biological replicates. Total number of cells analysed: *N* = 45,000 per strain.

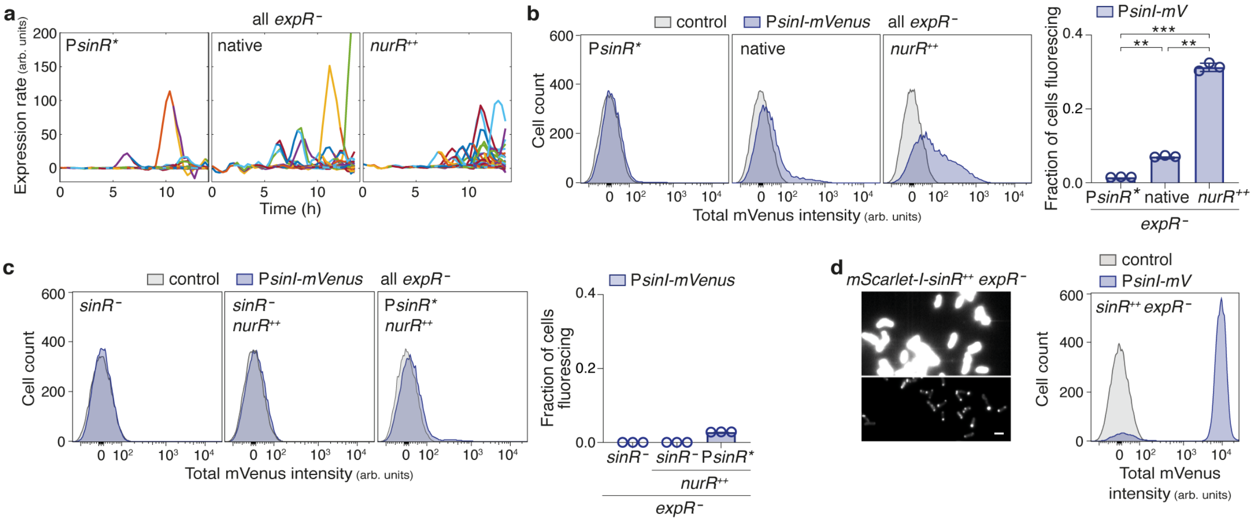

Supplementary Figure 5. SinR as a key factor. a Trajectories showing *sinI* expression pulses in colonies with different levels of *sinR* transcription, related to Fig. 2d. **b** Representative flow cytometry data (left) confirming relative differences in *sinI* expression pulse frequency: Histograms of fluorescence intensities from 15,000 cells carrying the *sinI* promoter-*mVenus* fusion (blue) with the same genetic modifications as in (**a**) and Fig. 2d, and 15,000 cells of the respective control strains without a fluorophore gene fusion (grey); the latter were used as donor strains for the experiment in Fig. 4b. Bar plots (right) indicate the fraction of cells in the samples displaying fluorescence intensities higher than those of the control strains in 3 biological replicates and their means and standard deviations. Statistical test, Welch's ANOVA test with post-hoc Dunnett's T3 multiple comparisons test. **, $P < 0.01$; ***, $P < 0.001$. Multiplicity-adjusted P values: P*sinR** vs. native 0.0012, P*sinR** vs. $nurR^{++}$ 0.0010, native vs $nurR^{++}$ 0.0015. Total number of cells analysed: $N = 45{,}000$ per strain. **c** Representative flow cytometry data (left) verifying that *nurR* exerts its effect on *sinI* expression via increasing SinR levels and not directly: *nurR* overexpression has no effect on fluorescence intensities from the *sinI* promoter-*mVenus* fusion in the $sinR^{-}$ strain, and hardly any on the strain carrying the *sinR* promoter mutation. Histograms of 15,000 cells carrying the *sinI* promoter-*mVenus* fusion (blue) and 15,000 cells of the respective control strains (grey) per genetic background. Bar plots (right) indicate the fraction of cells in the samples displaying fluorescence intensities higher than those of the control strains in 3 biological replicates and their means and standard deviations. Total number of cells analysed: $N = 45{,}000$ per strain. **d** Representative fluorescence microscopy image (left) and flow cytometry data (right) illustrating that direct overexpression of *(mScarlet-I-)sinR* disrupts the otherwise stochastic regulatory system: It not only abolishes heterogeneity in fluorescence, but also greatly augments fluorescence intensities. Fluorescence image acquired with identical settings as in Supplementary Fig. 4c, upper part reproduced with identical dynamic range as in Supplementary Fig. 4c, lower part with a 10-fold wider dynamic range. Scale bar, 2 µm. Flow cytometry data acquired with identical settings as in (**b**); blue, 15,000 cells carrying the *sinI* promoter-*mVenus* fusion; grey, 15,000 cells of the respective control strain. Total number of cells analysed: $N = 589$ for SMM data, 45,000 per strain for flow cytometry data, all in 3 biological replicates.

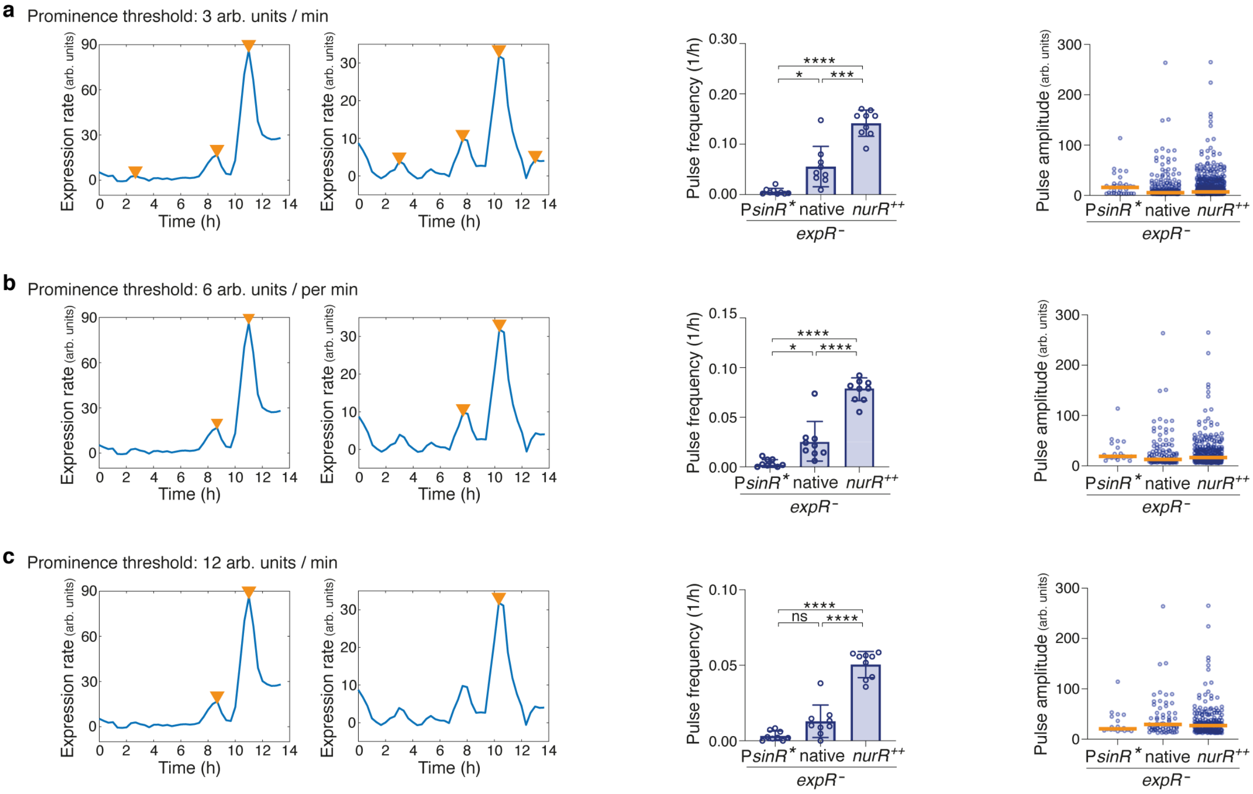

Supplementary Figure 6. Quantification of pulse data is robust to changes in peak prominence threshold. To test whether our results might have been falsified by our choice of threshold for what is considered a pulse, the data set with different *sinR* expression levels (Fig. 2d) was analysed with different threshold values, namely a minimal prominence backward in time of **a** 3 arbitrary units per minute, **b** 6 arbitrary units per minute as in the main analysis, and **c** 12 arbitrary units per minute. (Left) The histograms always show P*sinI-mVenus* expression rates of the same two representative cell lineages of the *nurR* overexpression strain (i.e., with elevated *sinR* expression levels); orange triangles mark what is considered a pulse with the respective threshold. Bar plots (middle) show mean P*sinI-mVenus* expression pulse frequencies for 9 colonies imaged on 3 different days determined with the respective threshold, and means and standard deviations of each data set; different thresholds yield different absolute pulse frequencies, but do not change relative differences between different genetic backgrounds. Statistical tests, Welch's ANOVA tests with post-hoc Dunnett's T3 multiple comparisons test. ns, not significant; *, $P < 0.05$; ***, $P < 0.001$; ****, $P < 0.0001$. Multiplicity-adjusted P values: P*sinR** vs. native 0.0181, P*sinR** vs. *nurR*$^{++}$ < 0.0001, native vs *nurR*$^{++}$ 0.0003 for prominence threshold 3 (**a**), P*sinR** vs. native 0.0290, P*sinR** vs. *nurR*$^{++}$ < 0.0001, native vs *nurR*$^{++}$ < 0.0001 for prominence threshold 6 (**b**), P*sinR** vs. native 0.0797, P*sinR** vs. *nurR*$^{++}$ < 0.0001, native vs *nurR*$^{++}$ < 0.0001 for prominence threshold 12 (**c**). (Right) Scatter dot plots showing amplitudes of individual pulses are identical above the highest threshold (**c**); median values indicated by orange bars are only slightly affected by the choice of threshold. Total number of cells analysed: N = 3,411 (P*sinR**), 2,900 (native), 2,440 (*nurR*$^{++}$).

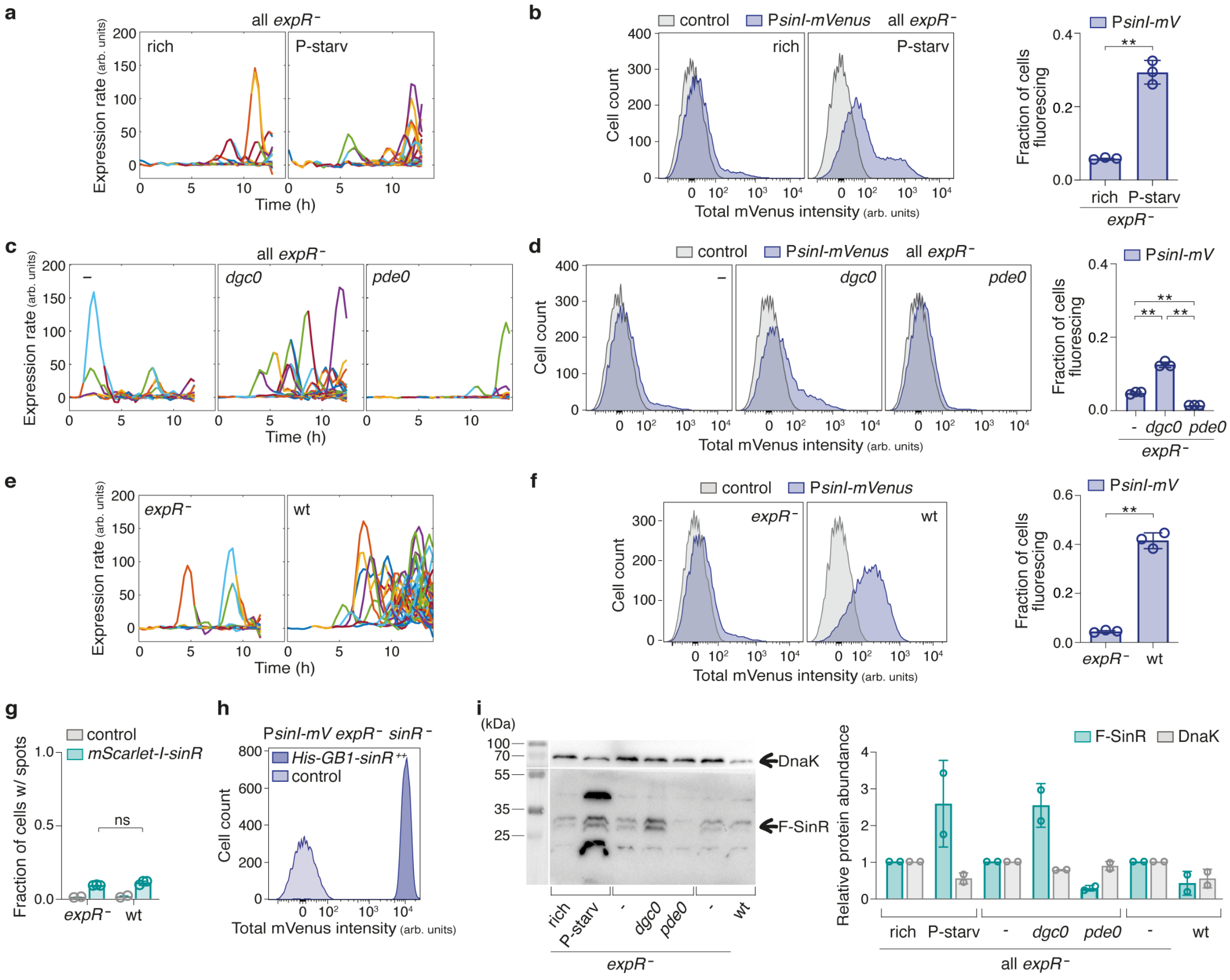

Supplementary Figure 7. Phosphate starvation, c-di-GMP and *expR* effects. a, **c**, **e** Representative trajectories of *sinI* expression (**a**) under rich growth conditions *vs.* phosphate starvation in *expR*$^-$ background, (**c**) in *expR*$^-$, *expR*$^-$ *dgc0* and *expR*$^-$ *pde0* strains, and (**e**) in *expR*$^-$ and wild-type strains, related to Fig. 3a-c. **b**, **d**, **f** Representative histograms (left) of 15,000 cells carrying the *sinI* promoter-*mVenus* fusion (blue) and 15,000 cells of the respective control strains (grey) per condition or genetic background, respectively, as in (**a**, **c**, **e**) determined by flow cytometry. Bar plots (right) indicate the fraction of cells in the samples displaying fluorescence intensities higher than those of the control strains in 3 biological replicates and their means and standard deviations. Statistical tests, two-tailed unpaired t-tests with Welch's correction for (**b**, **f**); Welch's ANOVA test with post-hoc Dunnett's T3 multiple comparisons test for (**d**). **, $P < 0.01$. P values (multiplicity-adjusted, if appropriate): rich vs. P-starv in *expR*$^-$ 0.0059 (**b**); *expR*$^-$ vs *expR*$^-$ *dgc0* 0.0012, *expR*$^-$ vs. *expR*$^-$ *pde0* 0.0079, *expR*$^-$ *dgc0* vs. *expR*$^-$ *pde0* 0.0032 (**d**); *expR*$^-$ vs. wt, 0.0021 (**f**). **g** Bar plots of single molecule microscopy data indicate that *expR* does not affect mScarlet-I-SinR levels. Open circles, data from 3 biological replicates; bars, means and standard deviations. Statistical test, two-tailed unpaired t-test with Welch's correction. ns, not significant; $P = 0.1408$. N = 1,300 (*expR*$^-$), 1,503 (wt). **h** Histograms of fluorescence intensities from the P*sinI-mVenus* fusion in a *sinR*$^-$ *expR*$^-$ strain (light blue) and the same strain overproducing His-GB1-SinR (dark blue) indicate *in vivo* activity of the fusion protein. Data representative of 2 biological replicates. **i** Western blot of Flag-tagged SinR representative of 2 biological replicates (left) and quantification of relative protein abundances in both replicates (right) confirm relative differences in SinR abundance under rich growth conditions vs. phosphate starvation in *expR*$^-$ background and *expR*$^-$, *expR*$^-$ *dgc0* and *expR*$^-$ *pde0* strains determined by single molecule microscopy; likewise, western blot data confirms that presence of *expR* in the wild-type background does not increase of F-SinR levels.

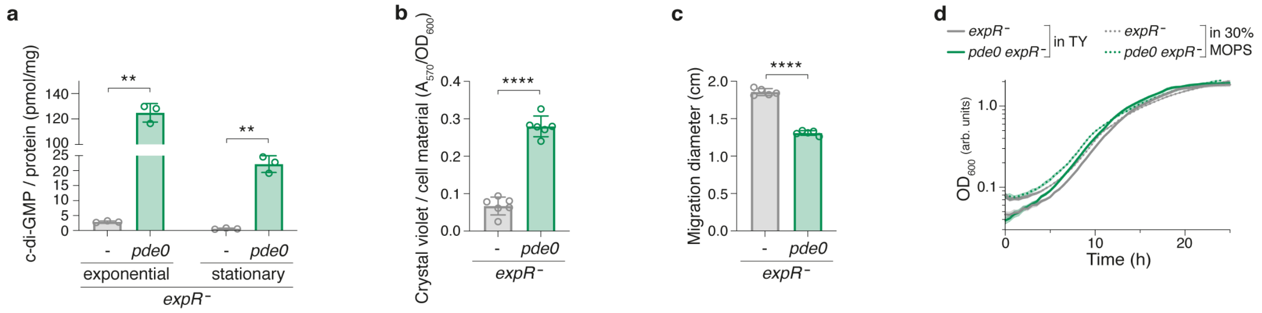

Supplementary Figure 8. The *expR*$^-$ *pde0* strain displays elevated c-di-GMP levels. a, b, c Bar graphs indicating elevated c-di-GMP levels (**a**), increased attachment detected by crystal violet staining (**b**), and decreased motility on soft agar plates (**c**) of the *expR*$^-$ *pde0* strain compared to the *expR*$^-$ parental strain. Bars represent means, error bars represent standard deviations, and open circles represent data from 3, 6 and 5 underlying biological replicates, respectively. Statistical tests, two-tailed unpaired t-tests with Welch's correction. **, $P < 0.01$; ****, $P < 0.0001$. **a** Whereas 2.9 ± 0.27 pmol c-di-GMP per mg protein were detected in exponential phase samples of the *expR*$^-$ parental strain, and 0.5 ± 0.21 pmol c-di-GMP per mg protein in *expR*$^-$ stationary phase samples, 124.8 ± 7.27 pmol c-di-GMP per mg protein were detected in exponential phase samples of the *expR*$^-$ *pde0* strain, and 22.1 ± 2.93 pmol c-di-GMP per mg protein in *expR*$^-$ *pde0* stationary phase samples. $OD_{600} \approx 0.45$, $P = 0.0012$ for exponential phase; $OD_{600} > 2$, $P = 0.0053$ for stationary phase. **b** Surface attachment in the *expR*$^-$ *pde0* strain is elevated approximately 4-fold compared to the parental strain ($P < 0.0001$), consistent with prior findings that c-di-GMP stimulates production of, e.g., arabinose-containing polysaccharide and other polysaccharides important for attachment in rhizobia[5–7]. Of note, these attachment-related polysaccharides are regulated differently than the exopolysaccharide galactoglucan which plays an important role in *S. meliloti* colony expansion and sliding motility[8–10] and is part of the organism's quorum sensing response[3,11]. **c** Motility of the *expR*$^-$ *pde0* strain is reduced by approximately 30% compared to the parental strain ($P < 0.0001$), consistent with prior findings that elevated c-di-GMP levels repress swimming motility in *S. meliloti*[5,6]. **d** Measurements of optical densities every 30 minutes for 25 hours indicate no difference in growth of the *expR*$^-$ *pde0* strain compared to the *expR*$^-$ parental strain. Data, means ± standard deviations of 3 biological replicates. For details on construction and characterization of the *expR*$^-$ *pde0* strain see Supplementary Methods 2.

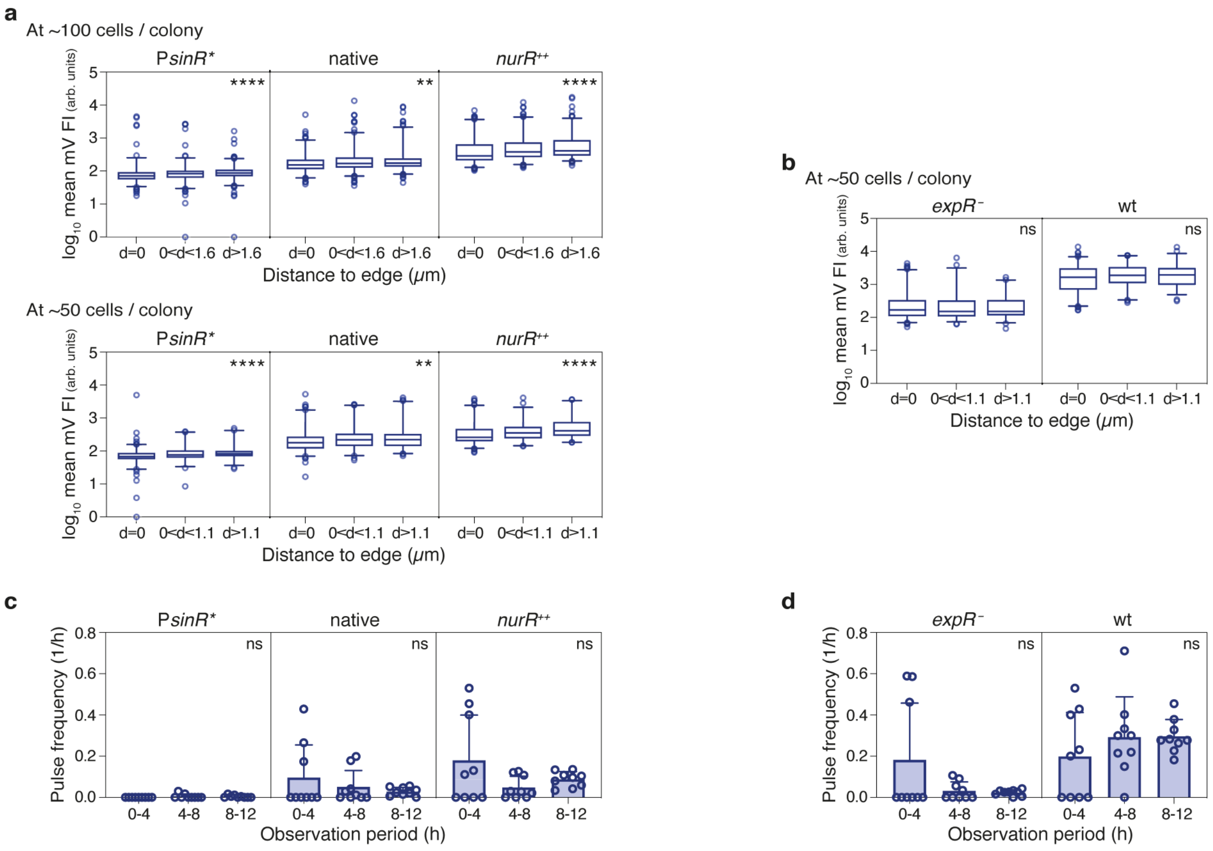

Supplementary Figure 9. Potential positional and temporal effects. a, b Analysis of two data sets, each consisting of 9 colonies per strain imaged on 3 different days, with respect to potential positional effects on *sinI* expression pulse frequency. Cells were grouped in three bins according to their distance from the colony edge: the colony boundary (d = 0 µm), followed by a ring of intermediate distance (e.g., 0 < d < 1.6 µm), and the colony centre (e.g., d > 1.6 µm); the boundary between the last two bins was chosen to yield equal-sized groups. The boxes in the plots indicate median, 25th and 75th percentile of mean mVenus fluorescence intensities of individual cells from the P*sinI-mVenus* fusion (log-transformed to account for high skew in distribution), the whiskers indicate 2.5^{th} and 97.5^{th} percentiles, individual points indicate outliers. Comparison of the three positional subgroups for each strain yielded small but significant effects for (**a**) the data set with different *sinR* expression levels (Fig. 2d) (medians 1.8523, 1.9175 and 1.9380 for P*sinR**, 2.1874, 2.2272 and 2.2391 for native, 2.4597, 2.5829 and 2.6148 for *nurR*$^{++}$ at ~100 cells; 1.8293, 1.8825 and 1.9156 for P*sinR**, 2.2525, 2.3378 and 2.3424 for native, 2.4133, 2.5478 and 2.6130 for *nurR*$^{++}$ at ~50 cells) and no significant differences for (**b**) the data set comparing receptor mutant (*expR*$^{-}$) and wild type (Fig. 3c) (medians 2.2287, 2.1817 and 2.1822 for *expR*$^{-}$, 3.2196, 3.2773, 3.2875 for wt). Mean fluorescence intensities were used as a proxy for pulses since pulse frequency must be determined over time, but cell position within the colony is bound to change over time. Statistical tests, Kruskal-Wallis tests; ns, not significant; **, $P < 0.01$; ****, $P < 0.0001$. Exact *P* values: < 0.0001 (P*sinR**), 0.0010 (native), < 0.0001 (*nurR*$^{++}$) at ~100 cells; < 0.0001 (P*sinR**), 0.0078 (native), < 0.0001 (*nurR*$^{++}$) at ~50 cells; 0.8698 (*expR*$^{-}$), 0.3275 (wt) at ~50 cells. **c, d** Similarly, developing microcolonies were analysed separately for the first, second and third four-hour period of the experiment. Plots show pulse frequencies for 9 colonies per strain and respective means and standard deviations for each observation period. Comparison of the temporal subgroups for each strain did not yield significant differences neither for (**c**) the data set with different *sinR* expression levels, nor for (**d**) the data set comparing receptor mutant (*expR*$^{-}$) and wild type. Statistical tests, Welch's ANOVA tests; ns, not significant.

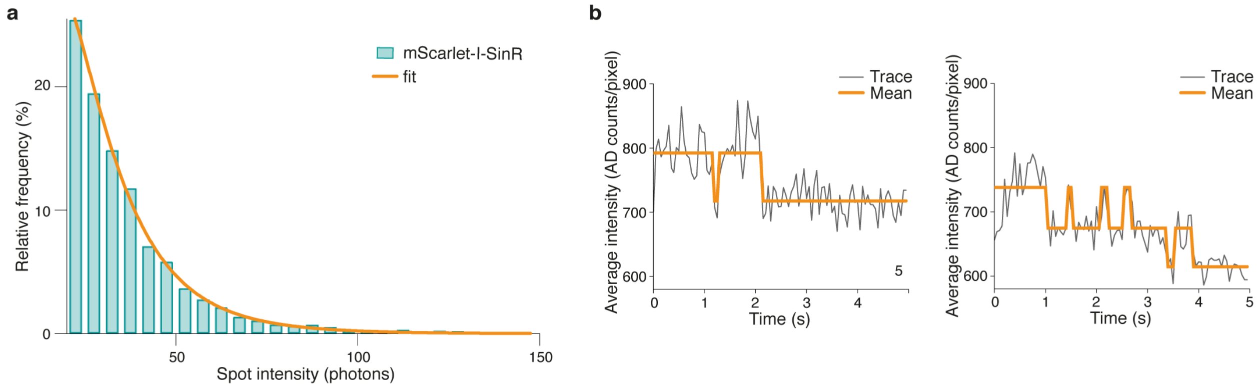

Supplementary Figure 10. mScarlet-I-SinR spots are homogeneous. Single molecule movies had been recorded with the aim to establish the frequency of mScarlet-I-SinR spots in *S. meliloti* populations; nevertheless, we can draw some conclusions on the nature of the observed spots with respect to the number of fluorophores they contain. **a** Histogram of fluorescence intensities of all spots detected in *mScarlet-I-sinR* strains in this work (Fig. 2c, 3a, b, Supplementary Fig. 7g) ($N = 3{,}277$) except for the *mScarlet-I-sinR* overexpression strain (Supplementary Fig. 5d). The frequency distribution of spot intensities can be fitted with a log-normal distribution (orange line), indicating a single, homogeneous population. Fitting models with multiple populations reduce to a single population and do not take the small increase between 70-100 photons into account that might be indicative of a second population. Taken together, this suggests that the mScarlet-I-SinR spots represent a largely homogeneous SinR population rather than a mixture of higher-order multimers[12]. Furthermore, the low intensities per spot, i.e., the very low number of emitted photons, indicates that the fluorescent spots are low copy number mScarlet-I-SinR spots, i.e. either SinR monomers or dimers. **b** Representative fluorescence intensity traces for selected spots, showing clear bleaching events of single fluorophores. The large majority of traces with bleaching events only have a single bleaching event until the background level is reached (left), but we also find some traces with two bleaching events (right). The step heights of bleaching events are the same for all traces (~ 70-90 AD counts). In addition, short fluorescence fluctuations are present in all traces, caused by brief ON- and OFF-blinking events of single mScarlet-I fluorophores. Taken together, this suggests a large population of mScarlet-I-SinR monomers and a small population of dimeric spots. Because our samples were chemically fixed, we cannot distinguish which of them are target-bound and which of them are part of a free, cytosolic population. Importantly, however, we find no evidence of higher-order multimers in both spot intensity and photobleaching trace analyses. Thus, this data adds strong evidence to the central finding of this work that heterogeneity in *sinI* expression is not caused by different expression levels of *sinR* but by the presence or absence of individual SinR molecules in each cell.

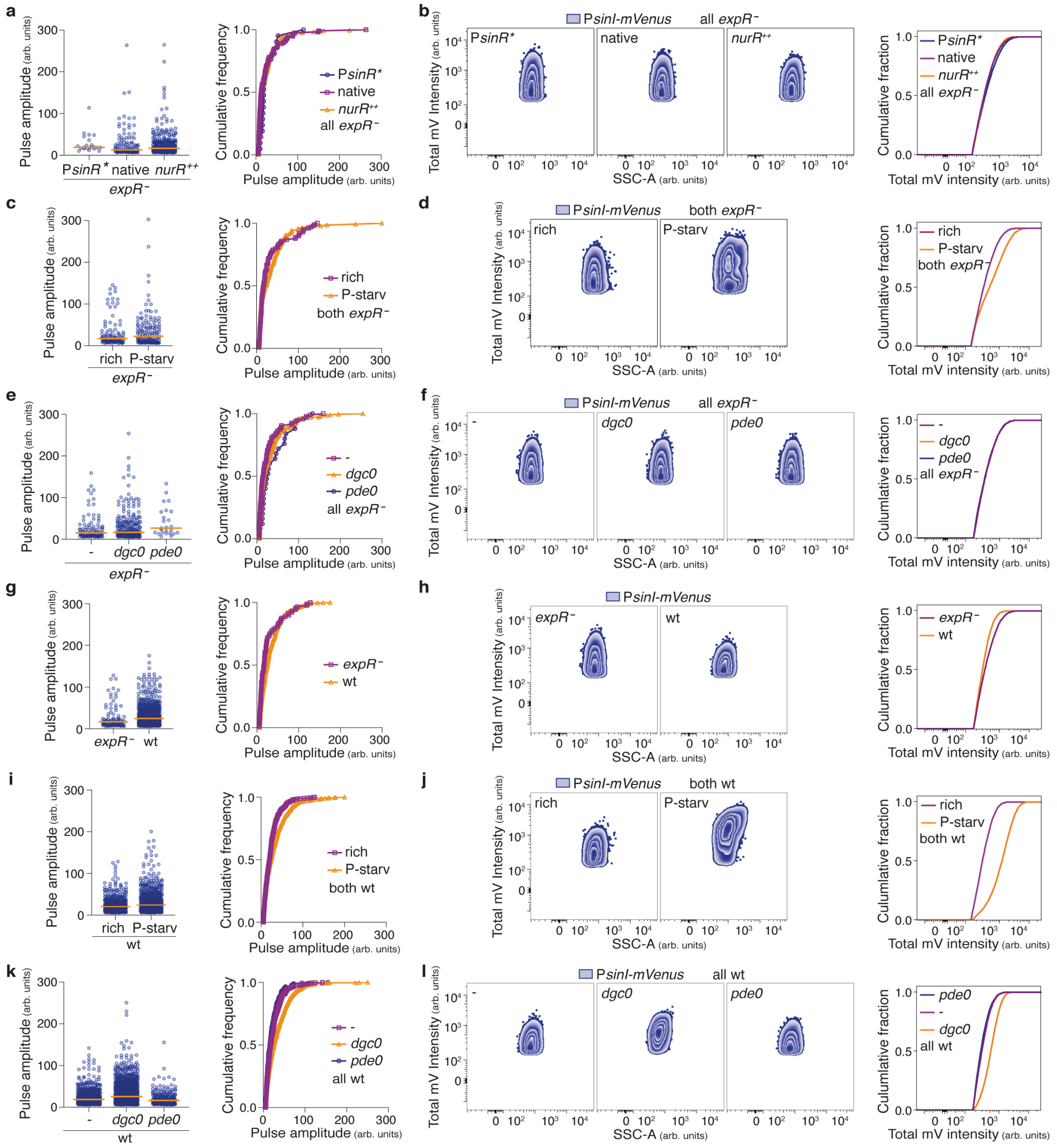

Supplementary Figure 11. Pulse amplitudes and fluorescence intensities. Comparison of *sinI* expression pulses (analysed with respect to pulse frequency/fraction fluorescing in Fig. 2 & 3 and Supplementary Fig. 5 & 7) with respect to (**a**, **c**, **e**, **g**, **i**, **k**) pulse amplitude in time-lapse microscopy and (**b**, **d**, **f**, **h**, **j**, **l**) fluorescence intensities of 'positive' cells in flow cytometry. Pulse amplitudes all vary 20-fold or more within strains or growth conditions. However, median pulse amplitudes do either not vary according to pulse frequencies at all (**a**, **e**), or only to a much smaller extent (**c**, **g**, **i**, **k**) (see Fig. 3f). Pulse amplitude data in (**a**, **c**, **e**, **g**, **i**, **k**) stems from strains/conditions with very different pulse frequencies, dot plots thus show varying numbers of data points and are also plotted as cumulative frequency distributions (right panels, respectively) for easier comparison. The increase in flow cytometry intensities correlating with very high pulse frequencies/fluorescing fractions are not reflected in pulse amplitude data and probably result from consecutive pulses that are still separated by time-lapse analysis, but add up in terms of total fluorescence intensities. Plots in (**b**, **d**, **f**, **h**, **j**, **l**) each show fluorescence data of 4,500 cells assessed as 'positive'.

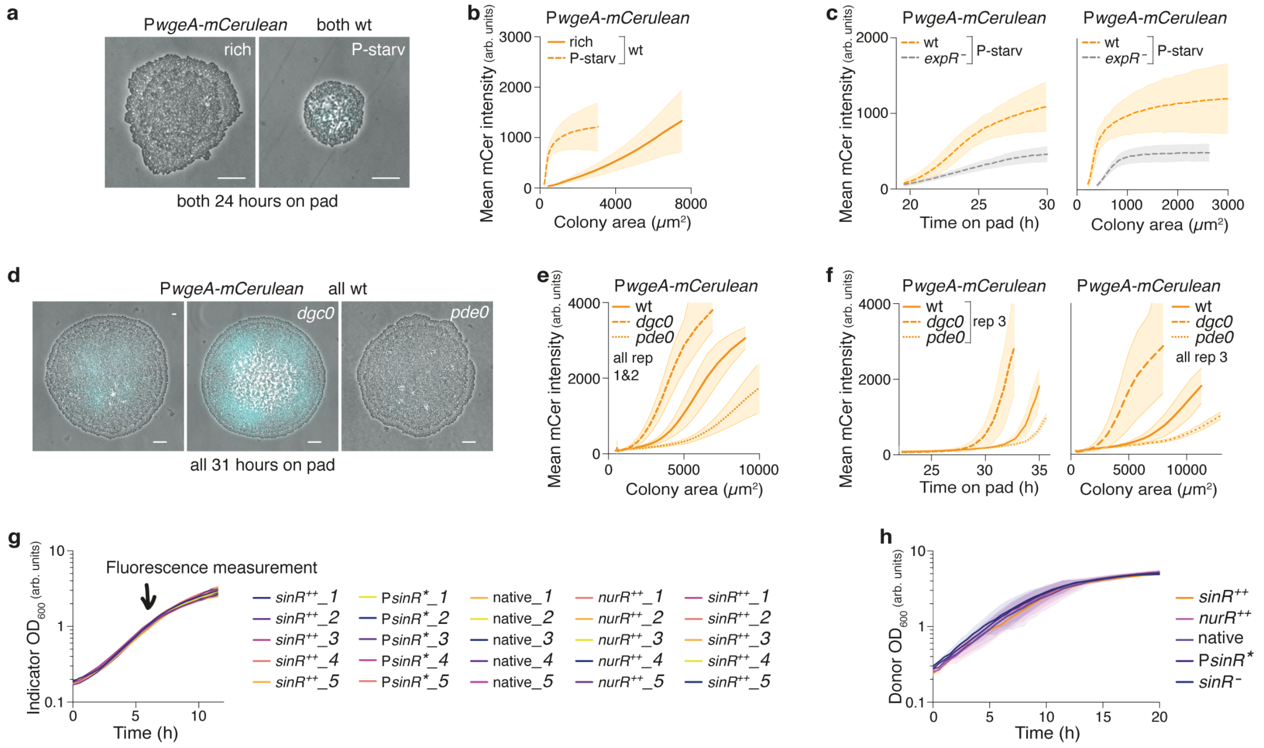

Supplementary Figure 12. Effects on the quorum sensing response. a Fluorescence microscopy snapshots of microcolonies of a wild-type strain carrying the *mCerulean* fluorophore gene fused to a promoter driving expression of genes involved in exopolysaccharide production (P*wgeA*), representing exemplary raw data underlying Fig. 4a (left). Microcolonies were grown for 24 hours under rich or phosphate starvation conditions, respectively. Scale bars, 10 µm. **b** Means and standard deviations of mean fluorescence intensities per colony from the P*wgeA-mCerulean* fusion of the same 9 wild-type colonies as shown in Fig. 4a, here plotted over colony area. **c** Means and standard deviations of mean P*wgeA-mCerulean* intensities per colony of 9 wild-type and *expR*$^-$ microcolonies, respectively, both grown under phosphate starvation conditions, plotted (left) over time and (right) colony area, respectively, illustrate that phosphate starvation induces expression of exopolysaccharide genes in *S. meliloti* even in absence of the AHL receptor, albeit with lower speed and intensity. This effect is due to additional direct regulation of the *wge* operon by phosphate starvation[13]. **d** Fluorescence microscopy snapshots of wt, *dgc0* and *pde0* microcolonies, all carrying the P*wgeA-mCerulean* fusion, representing exemplary raw data underlying Fig. 4a (right), and **e** means and standard deviations of mean P*wgeA-mCerulean* intensities per colony for 6 of the 9 colonies (rep. 1&2) shown in Fig. 4a plotted over colony area illustrate the onset of the quorum sensing response at different colony sizes depending on c-di-GMP levels. Scale bars, 10 µm. **f** Means and standard deviations of mean P*wgeA-mCerulean* intensities per colony for the third biological replicate for the c-di-GMP effect, plotted (left) over time and (right) colony area show similar relative, but different absolute behaviour. **g** Growth curves of the AHL indicator strain during incubation with supernatants from different strains and optical densities for Fig. 4b, controlling for possible effects of growth differences of the indicator strain on activation of the *sinI* promoter. The arrow indicates the time of the fluorescence measurement shown in Fig. 4b. **h** Growth curves of the supernatant donor strains for Fig. 4b indicate no effect on growth, i.e., no metabolic burden, by different *sinR* expression levels; donor strains did not carry the P*sinI-mVenus* fusion, i.e., expressed no fluorophore gene and are identical to the control strains for flow cytometry measurements shown in Supplementary Fig. 5b. Data represent means ± standard deviations of 2 biological replicates.

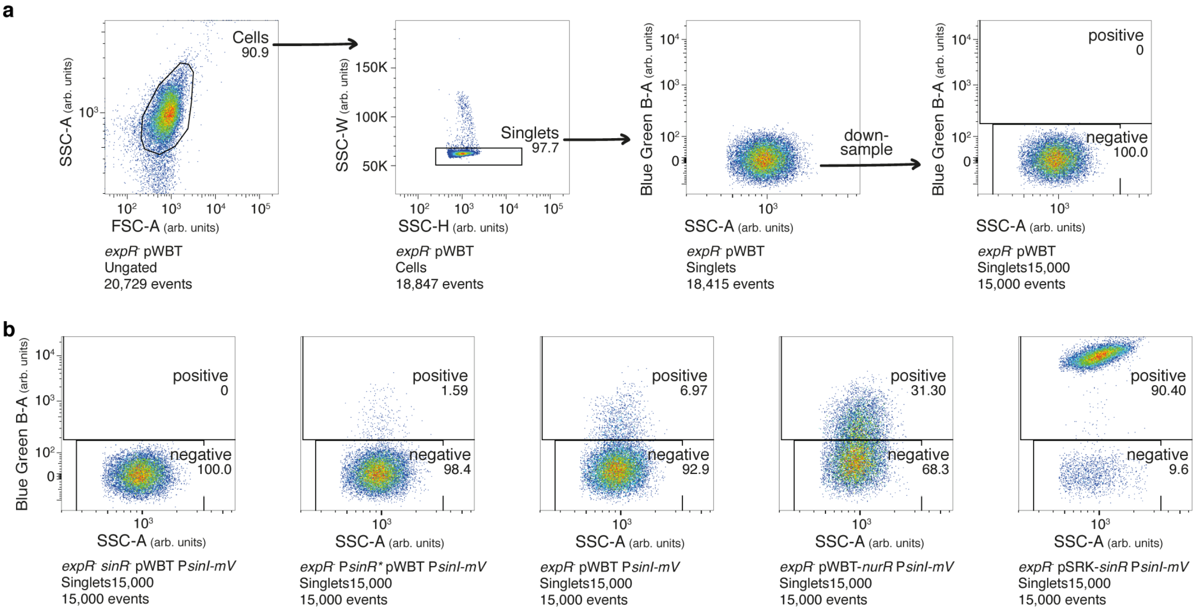

Supplementary Figure 13. Gating and quantification of flow cytometry data. a Gating was first performed on forward and side scatters (FSC and SSC, respectively) to remove dead cells and debris (SSC-A over FSC-A) and to exclude doublets (SSC-W over SSC-H). Subsequently, the number of samples was reduced to 15,000 events using the FlowJo Exchange DownSample plugin to ensure equal sample size. Strains lacking the *sinI* promoter-fluorophore gene fusion(s) with otherwise identical genetic backgrounds served as negative controls. **b** Cells in the read-out samples with higher fluorescence intensities than those of the respective control cells were assessed as ‘positive’. The fraction of cells per sample assessed as ‘positive’ and their corresponding median fluorescence values were determined with FlowJo. The data shown here were derived from strains with different *sinR* expression levels, from left to right: the *sinR* deletion, the *sinR* promoter mutant, the native *sinR* promoter, the native *sinR* promoter while overproducing its transcription activator NurR, and direct overproduction of SinR from a plasmid. The scatter plots shown in the second, third, and fourth panel thus correspond to the blue histograms in Supplementary Fig. 5b, the data in the first panel to the blue histogram in Supplementary Fig. 5c (left), and the last panel to the blue histogram in Supplementary Fig. 5d.

Supplementary Table 1. Data summary.

Experiment	Strain / condition	Pulse frequency (1/h)		Flow cytometry fraction		Pulse amplitude (au)		Median flow cytometry intensity (au)		Single molecule microscopy fraction*	
		Mean	SD	Mean	SD	Median	Q1/Q3	Mean	SD	mean	SD
Titrating *sinR* expression	PsinR*	0.0038	0.0039	0.0154	0.0006	18.87	16.15/40.46	384	10.02	0.1640	0.0301
	native	0.0254	0.0197	0.0715	0.0023	12.78	8.34/29.40	345	12.34	0.2377	0.0076
	nurR^{++}	0.0770	0.0114	0.3137	0.0110	16.51	9.67/31.86	355	5.51	0.4743	0.0413
Phosphate starvation in *expR*$^{-}$	*expR*$^{-}$ rich	0.0283	0.0129	0.0589	0.0025	17.04	10.41/34.19	339	6.66	0.1667	0.0153
	expR^{-} P-starv	0.0431	0.0165	0.2937	0.0320	22.44	9.11/47.24	481	21.50	0.4067	0.0451
Influence of c-di-GMP in *expR*$^{-}$	*expR*$^{-}$	0.0284	0.0219	0.0488	0.0036	15.36	8.34/31.17	313	4.16	0.1667	0.0153
	expR^{-} *dgc0*	0.0652	0.0259	0.1263	0.0075	16.55	8.57/40.50	318	1.00	0.2967	0.0153
	expR^{-} *pde0*	0.0080	0.0054	0.0149	0.0002	26.98	15.38/66.42	300	25.15	0.1300	0.0656
Influence of ExpR-AHL	*expR*$^{-}$	0.0291	0.0263	0.0456	0.0048	17.13	9.38/33.06	301	4.73	0.0926	0.0027
	wt	0.2771	0.0646	0.4173	0.0323	24.90	12.81/44.00	339	17.01	0.1189	0.0141
Phosphate starvation in wt	wt rich	0.1891	0.0478	0.6240	0.0667	20.28	10.98/31.49	354	52.17	ND	ND
	wt P-starv	0.3135	0.0586	0.9667	0.0092	24.44	12.34/44.17	1509	357.10	ND	ND
Influence of c-di-GMP in wt	wt	0.2288	0.0626	0.5843	0.1060	18.54	10.36/32.33	296	23.43	ND	ND
	dgc0	0.3462	0.0342	0.9510	0.0046	25.49	12.42/48.59	552	5.51	ND	ND
	pde0	0.0957	0.0466	0.2880	0.0061	16.16	9.28/27.17	238	0.58	ND	ND

Measures of central tendency and dispersion of P*sinI-mVenus* expression pulse frequencies, fractions of populations showing fluorescence from the P*sinI-mVenus* fusion in flow cytometry, pulse amplitudes and fluorescence intensities determined by flow cytometry, and of fractions of cells showing fluorescent spots in strains expressing *mScarlet-I-sinR* determined by single molecule microscopy. SD, standard deviation; Q1, first quartile; Q3, third quartile. Data from 3 biological replicates each. * no background subtraction; ND, not determined.

Supplementary Table 2. Strains.

Name	Relevant genotype	Figure(s)	Source or reference
E. coli			
DH5α	F⁻ *endA1 hsdR17*($r_K^-m_K^+$) *supE44 thi-1* λ⁻ *recA1 gyrA96 relA1 deoR* Δ(*lacZYA-argF*) U169 Φ80d*lacZ*ΔM15		[14]
S17-1	*E. coli* 294 Thi RP4-2-Tc::Mu-Km::Tn7 integrated into the chromosome		[15]
BL21(DE3)	F⁻ *ompT gal dcm lon hsdS*($r_B^-m_B^-$) λ(DE3 [*lacI lacUV5-T7p07 ind1 sam7 nin5*])		[16]
S. meliloti			
Sm2B3001	Wild type (*expR* restored), Nx^r, Sm^r	S2b; S7f, g; S11h, j, l	[17]
VBSm350	Sm2B3001 pK18*mob*3-2xP*sinI*	1b & S2a	This work
VBSm292	Sm2B3001 P*sinI-mScarlet-I*	precursor	This work
VBSm295	Sm2B3001 P*sinI-mScarlet-I* pKE-P*sinI-mNeonGreen*	S2b	This work
VBSm311	Sm2B3001 pKE-*mNeonGreen*	S2b	This work
VBSm1	Sm2B3001 P*sinI-mVenus*	S7f; S11h	[18]
VBSm314	Sm2B3001 P*sinI-mVenus* pK18*mob*3-P*wgeA-mCerulean*	3c; S11g; S12a-f	This work
VBSm43	Sm2B3001 *mScarlet-I-sinR*	S7g	This work
VBSm191	Sm2B3001 3x*flag-sinR*	S7i	This work
Rm2011	Wild type *expR*⁻ (*expR*::IS*Rm*2011-1), Nx^r, Sm^r	S2b; S4a, c, d; S7b, d, f; S11d, f, h	[19]
VBSm349	Rm2011 pK18*mob*3-2xP*sinI*	1b & S2a	This work
VBSm177	Rm2011 P*sinI-mScarlet-I*	precursor	This work
VBSm296	Rm2011 P*sinI-mScarlet-I* pKE-P*sinI-mNeonGreen*	S2b	This work
VBSm310	Rm2011 pKE-*mNeonGreen*	S2b	This work
VBSm3	Rm2011 P*sinI-mVenus*	S5d; S7b, d, f; S11d, f, h	This work
VBSm315	Rm2011 P*sinI-mVenus* pK18*mob*3-P*wgeA-mCerulean*	1c; 3a-c; S3; S5a, c, e; S11c, e, g; S12c	This work
VBSm237	Rm2011 P*sinI-mVenus* P*sinR**	precursor	This work
VBSm248	Rm2011 P*sinI-mVenus* P*sinR** pWBT	S5b; S11b	This work
VBSm20	Rm2011 P*sinI-mVenus* pWBT	S5b; S11b	This work
VBSm21	Rm2011 P*sinI-mVenus* pWBT-*nurR*	S5b; S11b	This work
VBSm316	Rm2011 P*sinI-Venus* P*sinR** pK18*mob*3-P*wgeA-mCerulean*	precursor	This work
VBSm323	Rm2011 P*sinI-mVenus* P*sinR** pK18*mob*3-P*wgeA-mCerulean* pWBT	2d; S5a; S11a	This work

VBSm324	Rm2011 P*sinI-mVenus* pK18*mob*3-P*wgeA-mCerulean* pWBT	2d; S5a; S11a	This work
VBSm325	Rm2011 P*sinI-mVenus* pK18*mob*3-P*wgeA-mCerulean* pWBT-*nurR*	2d; S5a; S11a	This work
VBSm235	Rm2011 P*sinR**	precursor	This work
VBSm246	Rm2011 P*sinR** pWBT	2d; S5b; S11b; 4b; S12g, h	This work
VBSm201	Rm2011 pWBT	2d; S5b; S11b; 4b; S12g, h	This work
VBSm22	Rm2011 pWBT-*nurR*	2d; S5b; S11b; 4b; S12g, h	This work
VBSm245	Rm2011 P*sinR** pWBT-*nurR*	S5c	This work
VBSm247	Rm2011 P*sinI-mVenus* P*sinR** pWBT-*nurR*	S5c	This work
VBSm24	Rm2011 P*sinI-mVenus* pSRKGm-*sinR*	S5d	This work
VBSm25	Rm2011 pSRKGm-*sinR*	S5d; 4b; S12g, h	This work
VBSm351	Rm2011 pK18*mob*3-P*sinR-mCherry*	2a	This work
VBSm353	Rm2011 pK18*mob*3-*sinRUTR-mCherry*	2a	This work
VBSm26	Rm2011 *mScarlet-I-sinR*	2c; S4c; 3a, b; S7g	This work
VBSm239	Rm2011 P*sinR*-mScarlet-I-sinR*	precursor	This work
VBSm250	Rm2011 P*sinR*-mScarlet-I-sinR* pWBT	2d	This work
VBSm30	Rm2011 *mScarlet-I-sinR* pWBT	2d	This work
VBSm32	Rm2011 *mScarlet-I-sinR* pWBT-*nurR*	2d	This work
VBSm242	Rm2011 *mScarlet-I-sinR* pSRKGm-*mScarlet-I-sinR*	S4D	This work
VBSm171	Rm2011 3x*flag-sinR*	2b; S4a, b; S7i	This work
VBSm27	Rm2011 P*sinI-mVenus mScarlet-I-sinR*	S4d	This work
VBSm302	Rm2011 P*sinI-mVenus* 3x*flag-sinR*	S4d	This work
VBSm53	Rm2011 Δ*sinR::synTer1*	precursor	This work
VBSm251	Rm2011 Δ*sinR::synTer1* pWBT	S5c; 4b; S12g, h	This work
VBSm217	Rm2011 Δ*sinR::synTer1* pWBT-*nurR*	S5c	This work
VBSm55	Rm2011 P*sinI-mVenus* Δ*sinR::synTer1*	S7h	This work
VBSm252	Rm2011 P*sinI-mVenus* Δ*sinR::synTer1* pWBT	S5c	This work
VBSm218	Rm2011 P*sinI-mVenus* Δ*sinR::synTer1* pWBT-*nurR*	S5c	This work
VBSm215	Rm2011 P*sinI-mVenus* Δ*sinR::synTer1* pSRKKm-GB1-*sinR*	S7h	This work
dgc0	Rm2011 Δ*pleD* Δ*SMc04015* Δ*SMb20523* Δ*SMc01464* Δ*SMa2301* Δ*SMb20389* Δ*SMb20447* Δ*SMb20900* Δ*SMc00038* Δ*SMa1548* Δ*SMc03178* Δ*SMa0137* Δ*SMc00992* Δ*SMc03942* Δ*SMc00887* Δ*SMc00033*	3b; S7d; S11f	5
VBSm267	*dgc0* P*sinI-mVenus*	S7d; S11f	This work

VBSm317	*dgc0* P*sinI-mVenus* pK18*mob*3-P*wgeA-mCerulean*	3b; S7c; S11e	This work
VBSm271	*dgc0* P*sinI-mVenus expR*$^+$	S11l	This work
VBSm319	*dgc0* P*sinI-mVenus expR*$^+$ pK18*mob*3-P*wgeA-mCerulean*	3d; 4a; S11k; S12d-f	This work
VBSm143	*dgc0 mScarlet-I-sinR*	3b	This work
VBSm169	*dgc0* 3x*flag-sinR*	S7i	This work
pde0	Rm2011 Δ*SMb21517* Δ*SMc00887* Δ*SMc03942* Δ*SMb20900* Δ*SMc03178* Δ*SMc00992* Δ*SMc00033* Δ*SMc00038* Δ*SMb20447* Δ*SMa0137* Δ*SMa1548* *SMc00074*(E746A) Δ*SMc03141*	3b; S7d; S11f	This work
VBSm269	*pde0* P*sinI-mVenus*	S7d; S11f	This work
VBSm318	*pde0* P*sinI-mVenus* pK18*mob*3-P*wgeA-mCerulean*	3b; S7c; S11e	This work
VBSm273	*pde0* P*sinI-mVenus expR*$^+$	S11l	This work
VBSm320	*pde0* P*sinI-mVenus expR*$^+$pK18*mob*3-P*wgeA-mCerulean*	3d; 4a; S11k; S12d-f	This work
VBSm145	*pde0 mScarlet-I-sinR*	3b	This work
VBSm173	*pde0* 3x*flag-sinR*	S7i	This work
Sm2B4001	Sm2B3001 *sinI*$^-$	Precursor	[20]
VBSm329	Sm2B4001 pK18*mob*2-P*pstS-mVenus*	P-starvation indicator	This work
VBSm168	Sm2B4001 pSRKGm-indicator	4b; S12g	This work

Strains shaded in grey represent precursor strains. 'S' in the Figure(s) column refers to Supplementary Figures.

Supplementary Table 3. Plasmids.

Plasmids	Relevant features	Source or reference
pK18*mobsacB*	Suicide vector; CAP binding site, *lac* promoter, *lac* operator, *lacZα*, *mob*, *sacB*, Km^r	[21]
pK18*mob*2	Suicide vector; CAP binding site, *lac* promoter, *lac* operator, *lacZα*, *mob*, Km^r	[22]
pK18*mob*3	Suicide vector; pK18*mob*2 derivative lacking CAP binding site, *lac* promoter, *lac* operator, *lacZα;* containing *mob*, Km^r	Courtesy of Marcel Wagner
pKEE	Suicide vector; pK18*mob*2 derivative containing 3' end of *exoP* and downstream sequence for integration on pSymB; *egfp*, Km^r	Courtesy of Elizaveta Krol
pSRKKm	Expression vector; *lacI^q*, CAP binding site, *lac* promoter, *lac* operator, *lacZα*, Km^r	[23]
pSRKGm	Expression vector; *lacI^q*, CAP binding site, *lac* promoter, *lac* operator, *lacZα*, Gm^r	[23]
pWBT	Expression vector; pSRKGm derivative containing tandem *lac* and T5 promoters, 3 *lac* operators	[24]
pEM-GB1	Expression vector; *lacI,* T7 promoter*, lac* operator*,* N-terminal 6xHis-tag, Factor Xa site, streptococcal protein G B1 domain (GB1), TEV site, Amp^r	[25,26]
pET28a	Expression vector; *lacI,* T7 promoter*, lac* operator, N-terminal 6xHis-tag, thrombin site, C-terminal 6xHis-tag, Km^r	Novagen
pK18*mobsacB*-P*sinI*-*mVenus*-*sinI*	pK18*mobsacB* derivative; containing *mVenus* w/ stop codon, flanked by P*sinI*-*sinI$_{1-27}$* and *sinI* UTR & gene	[18]
pK18*mobsacB*-P*sinI*-*mScarlet-I*-*sinI*	pK18*mobsacB* derivative; containing *mScarlet-I* (codon-optimized) w/ stop codon, flanked by P*sinI*-*sinI$_{1-27}$* and *sinI* UTR & gene	This work
pK18*mobsacB*-Δ*sinR::synTer1*	pK18*mobsacB* derivative; containing the synthetic multiple terminator site *synTer1* flanked by P*sinR* and the last 21 codons of *sinR,* downstream P*sinI* & 5' portion of *sinI*	This work
pK18*mobsacB*-P*sinR*-3x*flag*-*sinR*	pK18*mobsacB* derivative; containing 3x*flag*-tag coding sequence including a linker, flanked by P*sinR* and *sinR*	This work
pK18*mobsacB*-P*sinR*-*mScarlet*-*sinR*	pK18*mobsacB* derivative; containing *mScarlet-I* w/o stop codon followed by a linker, flanked by P*sinR*-*sinR$_{1-27}$* and *sinR*	This work
pK18*mobsacB*-P*sinR**-*sinR*	pK18*mobsacB* derivative; containing a P*sinR* w/ mutated NurR binding site, flanked by upstream sequence and *sinR*	This work
pK18*mobsacB*-P*sinR**-*mScarlet-I*	pK18*mobsacB* derivative; containing the P*sinR* w/ mutated NurR binding site, flanked by upstream sequence and *sinR$_{1-27}$*-*mScarlet-I*	This work

pK18*mobsacB*-*expR*$^{ATG-}$	pK18*mobsacB* derivative; containing *expR* from *S. meliloti* strain RM8530	[17]
pK18*mob*2-P*wgeA*-*mCer*/PT5-*mCh*/P*sinI*-*mV*	pK18*mob*2 derivative; containing P*wgeA*-*mCerulean,* PT5-*mCherry,* P*sinI*-*mVenus*	[24]
pK18*mob*2-P*pstS*-*mVenus*	pK18*mob*2 derivative; containing P*sinI*-*sinI*$_{1-27}$-*mCerulean,* P*sinR*-*mCherry,* P*pstS*-*pstS*$_{1-9}$-*mVenus*	This work
pK18*mob*3-2xP*sinI*	pK18*mob*3 derivative; containing P*sinI*-*sinI*$_{1-27}$-*mCerulean,* P*sinI*-*sinI*$_{1-27}$-*mCherry,* P*lac*-*mVenus*	This work
pK18*mob*3-P*sinR*-*mCherry*	pK18*mob*3 derivative; containing P*sinI*-*sinI*$_{1-27}$-*mCerulean,* P*sinR*-*mCherry,* P*lac*-*mVenus*	This work
pK18*mob*3-*sinRUTR*-*mCherry*	pK18*mob*3 derivative; containing P*sinI*-*sinI*$_{1-27}$-*mCerulean, sinRUTR*-*mCherry,* P*lac*-*mVenus*	This work
pK18*mob*3-P*wgeA*-*mCerulean*	pK18*mob*3 derivative; containing P*wgeA*-*wgeA*$_{1-15}$-*mCerulean,* P*trp*-*mCherry*	This work
pKE-P*sinI*-*mNeonGreen*	pKEE derivative; containing P*sinI*-*sinI*$_{1-27}$-*mNeonGreen* (codon-optimized)	This work
pKE-*mNeonGreen*	pKEE derivative; containing promoterless *mNeonGreen* (codon-optimized)	This work
pSRKKm-GB1-*sinR*	pSRKKm derivative; containing 6xHis-GB1-*sinR*	This work
pSRKGm-indicator	pSRKGm derivative lacking *lacI*^{q}, CAP binding site, *lac* promoter, *lac* operator & *lacZ*α; containing P*wgeA*-*mCerulean,* PT5-*mCherry,* P*sinI*-*mVenus*	This work
pSRKGm-*sinR*	pSRKGm derivative; containing *sinR*	This work
pSRKGm-*mScarlet-I-sinR*	pSRKGm derivative; containing *sinR*$_{1-27}$-*mScarlet-I-sinR*	This work
pWBT-*nurR*	pWBT derivative; containing *nurR*	[27]
pEM-GB1-*sinR*	pEMGB1 derivative; containing *sinR*	This work
pET28a-*expR*	pET28a derivative; containing *expR* fused to the N-terminal 6xHis-tag	This work

Supplementary Table 4. Oligonucleotides and synthetic genes.

Name	Sequence (5’-3’)	Construct(s)
sinR-X-B-fwd	catctagaggatcCATGGCTAATCAACAGGCTGTCCT	pK18*mobsacB*-P*sinI*-*mVenus*-*sinI*
PsinI-X-rev	cattctagaACCGTTTCCGTTCACTATCCT	
egfp-X-fwd	cattctagaATGGTGAGCAAGGGCGAGGAGCT	
egfp-K-rev	catggtaccTTACTTGTACAGCTCGTCCATGC	
sinI-K+TGA+RBS-fwd	ctaggtacctaaACGCATGGAGCGAAAAAATGATC	
sinI+66-E-rev	ctagaattCGTTAATTCGCAATGCCCGT	
sinR+209-PstI-f	catctgcagGGGGCTTTACAGGATCGAC	pK18*mobsacB*-P*sinI*-*mScarlet-I*-*sinI*
PsinI-r-mSc	gcccttgctgaccatACCGTTTCCGTTCACTATC	
PsinI-mSc	atagtgaacggaaacggtATGGTCAGCAAGGGC	
mSc-r-sinI	catgcgtgattacgcgatTCACTTATACAGCTCGTCCATG	
sinI-f-mSc	catggacgagctgtataagtgaATCGCGTAATCACGCATG	
sinI+66-X-r	cattctagaGTTAATTCGCAATGCCCG	
mScarlet-I, codon-optimized (Invitrogen)	ATGGTCAGCAAGGGCGAAGCCGTCATCAAAGAATTCA<br>TGCGCTTCAAGGTCCACATGGAAGGCTCGATGAACGG<br>CCACGAGTTCGAGATCGAAGGCGAAGGCGAAGGCCG<br>GCCGTATGAGGGCACCCAGACCGCCAAGCTCAAGGTC<br>ACCAAAGGCGGCCCGCTGCCGTTCTCGTGGGACATCC<br>TTTCGCCGCAGTTCATGTATGGCTCGCGCGCATTCATC<br>AAGCATCCGGCAGACATCCCGGACTATTATAAGCAGT<br>CGTTCCCGGAAGGCTTCAAGTGGGAGCGCGTCATGAA<br>CTTCGAGGATGGCGGCGCAGTCACCGTCACGCAGGAC<br>ACCTCGCTTGAGGACGGCACCCTCATCTATAAGGTCA<br>AGCTTCGCGGCACGAACTTCCCGCCGGATGGCCCGGT<br>CATGCAGAAAAAGACCATGGGCTGGGAAGCCTCGACC<br>GAGCGGCTCTATCCGGAAGATGGCGTCCTGAAGGGCG<br>ACATCAAGATGGCCCTCCGCCTCAAAGATGGCGGCCG<br>CTACCTCGCCGACTTCAAGACCACCTATAAGGCCAAG<br>AAACCGGTCCAGATGCCGGGCGCATATAACGTCGACC<br>GCAAGCTCGACATCACCTCGCACAACGAGGACTATAC	

	CGTCGTCGAGCAGTATGAGCGCTCGGAAGGCCGCCATTCGACCGGCGGCATGGACGAGCTGTATAAGTGA	
sinR-up-f	catgaattcGCTCAGCCTCCTGATCTCAC	pK18*mobsacB*-Δ*sinR::synTer1*
sinR-up-r	ACACCGAAACTTGCCCCTAT	
TT-f	TCGGTCAAGTGGGAAGAGTT	
TT-r	GCTTCCTCGCCGAGAGTT	
sinR-down-f	GTAGTGTCCAACACGCAAGC	
sinR-down-r	cattctagaGCCGTTTCAAGCGACATC	
sinR-up-TT bridg	CAAGTTTCGGTGTTCGGTCAAGTGGGA	
TT-sinR-down bridg	GGCGAGGAAGCGTAGTGTCCAAC	
PsinR-720-H-f	cataagcttGCGACCTTCTTCACCGATA	pK18*mobsacB*-P*sinR*-3x*flag*-*sinR*
PsinR-r-3xFl	tgtaatcgatatcgtgatccttgtaatcaccatcgtgatccttgtaatCCATGCCGTAACACCGAAACTTG	
sinR-f-3xFl	tcacgatatcgattacaaggatgacgatgacaagggatccggatccggatccATGGCTAATCAACAGGCTGT	
sinR-E-r	catgaattcTCAGATGGTGGGGATCAGAG	
PsinR-720-H-f	cataagcttGCGACCTTCTTCACCGATA	pK18*mobsacB*- P*sinR*-*mScarlet*-*sinR*
PsinR+27-Sal-r	catgtcgacATTGAGGACAGCCTGTTGATTAG	
mScarlet-I-Sal-f	catgtcgacATGGTTTCTAAAGGCGAAGCC	
mScarlet-I-TGA-B-r	catggatccagatccacctgcCTTGTACAATTCATCCATACCA	
sinR-X-B-f	cattctagaggatccATGGCTAATCAACAGGCTGTCCT	
sinR-E-r	catgaattcTCAGATGGTGGGGATCAGAG	
PsinR-720-Eh-f	gacatgattacgaattcGCGACCTTCTTCACCGATA	pK18*mobsacB*- P*sinR**-*sinR*
PsinR*-r	tgcaataaagcttggcaGGTGCAGTAATCCCGCTTA	
PsinR*-f	ccaagcttTATTGCACTAGACAAAACCGG	
sinR-Bh-r	tcgactctagaggatccTCAGATGGTGGGGATCAGAG	
PsinR-720- Eh-f	gacatgattacgaattcGCGACCTTCTTCACCGATA	pK18*mobsacB*- P*sinR**-*mScarlet-I*
PsinR*-r	tgcaataaagcttggcaGGTGCAGTAATCCCGCTTA	
PsinR*-f	ccaagcttTATTGCACTAGACAAAACCGG	
mSca-Bh-r	tcgactctagaggatccCTTGTACAATTCATCCATACCA	
PpstS-A-F	catgacgtcTTGCGATCGTCAAGCATATC	pK18*mob*2- P*pstS*-*mVenus*
PpstS-S-r	catgtcgacAGATTTCATGAATGTTCTCCC	

PsinI-M-f<br>PsinI-K-r	catacgcgtCAACGATTCTCGGCATATCC<br>catggtaccACCGTTTCCGTTCACTATCCT	P*sinI-mCerulean* on pK18*mob*3-2xP*sinI*
PsinI-B-f<br>PsinI-X-r	catggatccCAACGATTCTCGGCATATCC<br>cattctagaACCGTTTCCGTTCACTATCCT	P*sinI-mCherry* on pK18*mob*3-2xP*sinI*
Plac145-AatII-f<br>Plac-rbs-S-r	catgacgtcTTGGCCGATTCATTAATGCAG<br>catgtcgaccatttttcgctccatgCCTGTGTGAAATTGTTATCCGC	P*lac-mVenus* on pK18*mob*3-2xP*sinI*
PsinR-B-f<br>mCh+-S-r	gatggatccCGCATATTCTGTCGCCGT<br>ATTGCGGGAGCTCTTACTTG	pK18*mob*3-P*sinR-mCherry*
sinRUTR-X-f<br>mCh+-S-r	catggatccggtgttacggcatgtctaga<br>ATTGCGGGAGCTCTTACTTG	pK18*mob*3-*sinRUTR-mCherry*
PwgeA-K-rev<br>PwgeA-M/Xho-fwd	catggtaccTTCCAAAGTGGCCATCTGCTT<br>tagacgcgttagctcgagTTCGGGAGGACTGACCTGT	pK18mob3-P*wgeA-mCerulean*
Ptrp-fwd oligo1<br>Ptrp-fwd oligo2<br>Ptrp-rev oligo1<br>Ptrp-rev oligo2	gatccgcggccgcgaaatgagctgttgacaattaatcatcg<br>aactagttaactagtacgcaagttcacgtaaaaagggtcacacaggaaat<br>agttcgatgattaattgtcaacagctcatttcgcggccgcg<br>ctagatttcctgtgtgaccctttttacgtgaacttgcgtactagttaact	P*trp-mCherry*<br>on pK18mob3-P*wgeA-mCerulean*
Term-exch-Sac-fwd<br>Term-exch-Sac-rev	ataaaacgaaaggctcagtcgaaagactgggcctttcgttttaa<br>agctttaaaacgaaaggcccagtctttcgactgagcctttcgttttatagct	Removal of T1 terminators & *mVenus* on pK18mob3-P*wgeA-mCerulean*
PsinI-K-f<br>PsinI-r-mN<br>mNe-f-sinI<br>mNe-E-r<br>mNeonGreen, codon-optimized (Invitrogen)	catggtaccCAACGATTCTCGGCATATCC<br>cgcctttcgacaccatACCGTTTCCGTTCACTATC<br>gatagtgaacggaaacggtATGGTGTCGAAAGGCG<br>catgaattcTCACTTGTAGAGTTCATCCATG<br>ATGGTGTCGAAAGGCGAAGAGGACAACATGGCCTCGC<br>TTCCGGCAACGCACGAGCTGCACATCTTCGGCAGCAT<br>CAACGGCGTCGACTTCGACATGGTCGGCCAAGGCACG<br>GGCAACCCGAACGACGGCTATGAGGAACTCAACCTCA<br>AGTCGACCAAGGGCGACCTCCAGTTCTCGCCGTGGAT<br>CCTCGTGCCGCACATCGGCTATGGCTTCCACCAGTATC<br>TCCCGTATCCGGACGGCATGTCGCCGTTCCAGGCAGC<br>CATGGTCGACGGCTCGGGCTATCAGGTCCACCGCACC<br>ATGCAGTTCGAAGATGGCGCAAGCCTCACCGTCAACT<br>ATCGCTATACCTATGAGGGCTCGCACATCAAAGGCGA	pKE-P*sinI-mNeonGreen*

	GGCCCAGGTCAAAGGCACGGGCTTCCCGGCAGATGGC<br>CCCGTGATGACCAACTCGCTCACCGCAGCCGACTGGT<br>GCCGCTCGAAGAAAACCTATCCGAACGACAAGACCAT<br>CATCTCGACGTTCAAGTGGTCGTATACCACCGGCAAC<br>GGCAAGCGCTATCGCTCGACCGCACGCACCACGTATA<br>CCTTCGCCAAGCCGATGGCAGCCAACTATCTCAAGAA<br>CCAGCCGATGTATGTGTTCCGCAAGACCGAGCTGAAG<br>CACAGCAAGACGGAACTGAACTTCAAAGAGTGGCAG<br>AAGGCGTTCACCGACGTCATGGGCATGGATGAACTCT<br>ACAAGTGA	
mN-K-f<br>mNe-E-r	catggtaccATGGTGTCGAAAGGCG<br>catgaattcTCACTTGTAGAGTTCATCCATG	pKE-*mNeonGreen*
GB1-N-f<br>GB1-sinR-S-r	catcatatgCATCATCATCATCATCATAGCAG<br>caggagctctcAGATGGTGGGGA	pSRKKm-GB1-*sinR*
sinR-N-T5rbs-fwd<br>sinR-Sac-rev	tcccatatgaaagaggagaaattaactATGGCTAATCAACAGGCTGTC<br>catgagctcTCAGATGGTGGGGATCAGA	pSRKGm-*sinR*
sinR-Nh-f<br><br>mScarlet-I-TGA-B-r<br>sinR-X-B-f<br>sinR-Sh-r	aacaatttcacacaggaaacagcatcccatatgaaagaggagaaattaactATGGCT<br>AATCAACAGGCTGTCCT<br>catggatccagatccacctgcCTTGTACAATTCATCCATACCA<br>cattctagaggatccATGGCTAATCAACAGGCTGTCCT<br>agcggccgccaccgcggtggagctcTCAGATGGTGGGGATCAGAG	pSRKGm-*mScarlet-I-sinR*
SmSinR_F<br>SmSinR_R	ttaaccATGGCTAATCAACAGGCT<br>ttaaggatccttAGATGGTGGGGATCAG	pEM-GB1-*sinR*
expR-N-f<br>expR+127-H-r	catcatATGAATATTACGTTGCTCGTACAGTTTCTG<br>cataagcttACGGGCACAGGAATGAAG	pET28a-*expR*
[Cy3]264f<br>440r	[Cy3]ACGCTGTTCGACATGCTCT<br>CGTGATTACGCGATGTAGC	P*sinI* EMSA fragment

With the exception of synthetic genes, upper case letters indicate bases annealing to the template in PCR, lower case letters indicate tails containing, e.g., restriction sites or linkers. [Cy3], fluorescent dye label.

Supplementary Methods 1. Details on strain constructions.

For the *sinI* promoter-*mVenus* fusion analysed by time-lapse fluorescence microscopy and flow cytometry, a 678 bp fragment containing the *sinI* promoter, 5' untranslated region (UTR) and first nine codons of *sinI*, the *mVenus*[28] gene including stop codon, and a 729 bp fragment containing the *sinI* coding sequence preceded by its native ribosome binding site were cloned into pK18*mobsacB*[21] in a step-wise fashion using HindIII, XbaI, KpnI and EcoRI restriction enzymes (at one step taking advantage of a HindIII site within the fragment including the *sinI* promoter); the final construct pK18*mobsacB*-P*sinI*-*mVenus*-*sinI* thus carried the *mVenus* gene flanked upstream by the *sinI* promoter and downstream by the *sinI* coding sequence. After conjugation and double homologous recombination in *S. meliloti*, the chromosomal *sinI* locus comprises the native *sinI* promoter, UTR and first nine codons followed by an XbaI site, the *mVenus* gene including a KpnI site immediately preceding the stop codon, and the *sinI* coding sequence preceded by its native ribosome binding site.

The *sinI* promoter-*mScarlet-I* fusion analysed by flow cytometry was constructed in a similar fashion as the *sinI* promoter-*mVenus* fusion. However, here the *sinI* gene is preceded by the entire *sinI* UTR. Fusion of the flanking regions with codon-optimized *mScarlet-I*[29] was performed by ligase cycling reaction[30], and restriction digestion followed by ligation was only employed for cloning of the preassembled insert into pK18*mobsacB*, resulting in pK18*mobsacB*-P*sinI*-*mScarlet-I*-*sinI*. Thus, *S. meliloti* strains carrying this fusion at the native *sinI* locus have no restriction site introduced by cloning in between the *sinI* promoter, UTR and first nine *sinI* codons and the downstream fluorophore gene, and no restriction site in between the fluorophore gene and the downstream *sinI* UTR and coding sequence.

The *sinI* promoter-*mNeonGreen* fusion likewise analysed by flow cytometry comprises the 259 bp sequence upstream of the *sinI* transcription start site (TSS), again followed by the *sinI* UTR and first nine *sinI* codons, fused to codon-optimized *mNeonGreen*[31] via overlap extension PCR[32]. Cutting with KpnI and EcoRI restriction enzymes, the assembled insert was cloned into a suicide vector carrying a 964 bp fragment amplified from *S. meliloti* megaplasmid pSymB, i.e., the 3' 676 bp of the *exoP* gene and the subsequent 288 bp including a terminator sequence immediately downstream of *exoP*. Thus, in *S. meliloti* the final construct pKE-P*sinI*-*mNeonGreen* integrates into the essential megaplasmid pSymB[4] by homologous recombination, and readthrough of *exoP* transcription into the *sinI* promoter-*mNeonGreen* fusion is blocked by the included terminator sequence (as demonstrated for the analogous promoter probe vector pSRPP18[33]). The corresponding control plasmid pKE-*mNeonGreen* carrying a promoterless *mNeonGreen* was obtained by amplification of only the codon-optimized *mNeonGreen* gene with the same reverse primer and an *mNeonGreen* forward primer including a KpnI site.

The construct comprising two identical copies of the *sinI* promoter studied by fluorescence microscopy is based on a modified version of a triple reporter cassette[24] allowing for parallel monitoring of the activities of up to three different promoters fused to the *mCerulean*[34], *mCherry*[35] and *mVenus* genes, respectively; in contrast to the earlier version, the modified cassette includes the *rpoC/thrA* tandem terminator inserted between the *mCerulean* and *mCherry* genes, and only two copies instead of four of the T1 transcription terminator between the *mCherry* and *mVenus* genes[27]. Both *sinI* promoter-fluorophore gene fusions comprise the same 259 bp sequence upstream of the *sinI* TSS as the *sinI* promoter-*mNeonGreen* fusion, again followed by the *sinI* UTR and the first nine *sinI* codons; these fragments were cloned in front of *mCerulean* using MluI/KpnI restrictions sites and in front of *mCherry* using BamHI/XbaI restriction sites, respectively. The triple reporter cassette was then cut out of its original vector pK18*mob*2 by cutting with EcoRI/HindIII restriction enzymes (and additionally with MreI to allow for separation of fragments via gel electrophoresis) and cloned into the EcoRI/HindIII-digested suicide plasmid pK18*mob*3, derived from pK18*mob*2[21] by deletion of the *lac* promoter preceding the multiple cloning site. Furthermore, to allow for microscopy image segmentation, a 174 bp fragment

including the *lac* promoter amplified from pSRKGm[23] combined with the *sinI* ribosome binding site was cloned in front of the *mVenus* gene using AatII/SalI restriction enzymes. In both *S. meliloti* strains carrying the final construct pK18*mob*3-2xP*sinI* in this work, the vector had integrated into the chromosome via the *sinI* promoter-*mCherry* fusion, so that the two fusions are separated by approximately 4 kb of plasmid DNA; a third, dark copy of the *sinI* promoter regulates expression of *sinI* in these strains (Fig. 1b & Supplementary Figure 2a).

The *sinR* promoter-*mCherry* construct analysed by fluorescence microscopy in turn is a derivative of the above 2xP*sinI* construct. The entire *sinR* promoter-*mCherry* fusion (including a 294 bp fragment of *sinR* promoter, UTR and start codon) was amplified from an earlier triple reporter cassette located on pK18*mob*2[27] and used to replace the *sinI* promoter-*mCherry* fusion by cutting both the PCR product and the double *sinI* promoter-fluorophore vector with BamHI/SacI. Thus, this construct pK18*mob*3-P*sinR*-*mCherry* likewise carries a *lac* promoter-*mVenus* fusion for microscope image segmentation, and the *sinI* promoter-*mCerulean* fusion. The corresponding control plasmid pK18*mob*3-*sinRUTR-mCherry* lacks the *sinR* promoter, but includes the *sinR* ribosome binding site to enable translation of potential transcriptional readthrough. Both in the *S. meliloti* strain carrying the read-out construct and the *S. meliloti* strain carrying the control construct the plasmid integrated into the chromosome via the *sinI* promoter-*mCerulean* fusion.

The *wgeA* promoter-*mCerulean* construct assayed in long-term time-lapse fluorescence microscopy is a derivative of yet another triple reporter cassette, carrying a 303 bp fragment including the *wgeA* promoter, UTR and the first five *wgeA* codons in front of *mCerulean* inserted via KpnI/MluI restriction sites[27]. The *trp* promoter and UTR combined with the *lac* ribosome binding site was assembled from oligonucleotides and inserted in front of *mCherry* via BamHI/XbaI restriction sites to allow for microscope image segmentation. The *mVenus* gene and preceding restriction sites and T1 tandem terminators in turn were removed and exchanged for a short terminator sequence in order to allow use of this construct in combination with the chromosomal *sinI* promoter-*mVenus* fusion. The final cassette comprising the *wgeA* promoter-*mCerulean* fusion, the *rpoC/thrA* tandem terminator and the *trp* promoter-*mVenus* fusion was cloned into pK18*mob*3 via EcoRI and HindIII restriction sites; in *S. meliloti* strains carrying this construct pK18*mob*3-P*wgeA-mCerulean* the plasmid integrated into pSymB via the *wgeA* promoter-*mCerulean* fusion.

The *pstS* promoter-*mVenus* fusion employed to verify the onset of phosphate starvation in time-lapse fluorescence microscopy experiments likewise is part of a triple reporter construct, albeit on pK18*mob*2; a 298 bp fragment including the *pstS* promoter and the first three *pstS* codons was cloned in front of the *mVenus* gene using AatII and SalI restriction enzymes. The final plasmid (that also includes the above-described *sinR* promoter-*mCherry* fusion, and the above described *sinI* promoter-*mCerulean* fusion) was integrated into the *S. meliloti sinI* mutant Sm2B4001[20], so that the resulting scavenger/indicator cells do not contribute to AHL levels on the agarose pad during experiments.

The AHL indicator plasmid used in the plate reader experiment was built by cutting both the original pK18*mob*2-triple reporter cassette[24] and pSRKGm[23] with EcoRI/HindIII, thus removing *lacI,* the *lac* promoter and part of *lacZα* from the latter, and subsequent ligation of the pSRKGm backbone and the triple reporter cassette, yielding pSRKGm-indicator.

For the *mScarlet-sinR* translational fusion employed in single molecule microscopy, a 733 bp fragment containing the *sinR* promoter, UTR and first nine codons, the *mScarlet-I* gene without stop codon, but including a linker (AGGSGS) added via a primer tail, and the *sinR* coding sequence were cloned into pK18*mobsacB* in a step-wise fashion using SalI/HindIII, EcoRI/XbaI and BamHI/SalI restriction enzymes. After conjugation and double homologous recombination in *S. meliloti*, the fusion protein was expressed from the native *sinR* promoter.

For the N-terminally 3xFLAG-tagged SinR, a 709 bp fragment containing the *sinR* promoter and the *sinR* gene were amplified separately, with the coding sequence for the 3x-FLAG tag (DYKDHDGDYKDHDIDYKDDDDK) and the linker (GSGSGS) comprised as overlapping tails in the inner primers; the two fragments were then fused via overlap extension PCR and cloned into pK18*mobsacB* using PstI (cutting within the *sinR* promoter fragment) and EcoRI. The construct was subsequently integrated into the *S. meliloti* genome via double homologous recombination, replacing the native *sinR* gene, so that strains carrying this construct produced 3xFLAG-tagged SinR from the native *sinR* promoter.

For production and purification of His_6-GB1-SinR, the *sinR* gene was cloned via NcoI/BamHI restriction sites into pEM-GB1; this vector links a 6xHis-tag and the coding sequence of the immunoglobulin-binding domain of streptococcal protein G (GB1 domain) to the N-terminus of the protein of interest[25,26], thus enhancing solubility[36,37]. To examine *in vivo* activity of the His-GB1-SinR fusion protein, the respective coding sequence was amplified from pEM-GB1-*sinR* and cloned into the broad host range expression plasmid pSRKKm which was then conjugated into the *S. meliloti sinR*$^-$ mutant (Δ*sinR::synTer1*) described below.

For production and purification of N-terminally His_6-tagged ExpR, the *expR* gene was amplified via colony PCR from Sm2B3001 and cloned into the expression vector pET28a using NdeI and HindIII restriction sites.

To completely abolish *sinR* expression, a synthetic multiple terminator site (*synTer1*)[38] flanked by the *sinR* promoter (up to -7 bp from the *sinR* start codon) and the last 21 codons of *sinR* followed by the *sinI* promoter and most of the *sinI* coding sequence was cloned into pK18*mobsacB*; after integration of the resulting plasmid pK18*mobsacB*-Δ*sinR::synTer1* into the *S. meliloti* genome, the resulting *sinR*$^-$ strains thus lacked *sinR*, but carried the *sinR* promoter and most of its UTR followed by the terminator sequence blocking potential read-through, the 3' end of *sinR* and the native *sinI* promoter and coding sequence.

To specifically reduce *sinR* expression levels, a mutated version of the *sinR* promoter in which the 5' half of the NurR binding site had been exchanged (GTTTATGAAATATTGCACTA to TGCCAAGCTTTATTGCACTA) according to[27] was cloned into pK18*mobsacB* either in front of *sinR*$_{1-27}$-*mScarlet-I* (for the single molecule microscopy strain) or in front of *sinR* (for time-lapse microscopy and flow cytometry strains); due to presence of many restriction sites in the assembled fragment, only the vector was digested with EcoRI and BamHI, while the outer primers were designed with homology to the vector, and fusion of vector and insert was done by aqua cloning[39]. The mutated promoter was then stably integrated into the genomes of the respective strains by double homologous recombination.

For direct overexpression of *sinR*, the *sinR* coding sequence preceded by the T5 ribosome binding site (added upstream of *sinR* during amplification by means of a primer tail) was cloned into pSRKGm using NdeI/SacI restriction sites.

For overexpression of *mScarlet-I-sinR*, the two genes were amplified separately, *sinR*$_{1-27}$-*mScarlet-I* as described for overexpression of *sinR* with preceding T5 ribosome binding site, and as described for *mScarlet-I-sinR* without stop codon, but including a linker. Both PCR fragments were then digested with BamHI, subsequently ligated, and the product was inserted into the NdeI/SacI-digested pSRKGm via aqua cloning.

The plasmid pK18*mobsacB*-*expR*$^{ATG-}$ was used to restore the AHL receptor in strains derived from *S. meliloti* Rm2011 as these strains carry an insertion sequence in the *expR* locus SMc03899-SMc03896 (*expR*::IS*Rm*2011-1)[17].

Supplementary Methods 2. Construction and characterization of the *expR⁻ pde0* strain.

The *S. meliloti* Rm2011 genome encodes 22 c-di-GMP-related proteins, 13 of which contain predicted phosphodiesterase (PDE) domains[5]. For the *expR*⁻ *pde0* strain, these 13 genes were deleted or, in case of *SMc00074*, replaced with a variant encoding an active site mutation via double homologous recombination using the suicide vector pK18*mobsacB*[21].

Gene	Plasmid	Primers / Source
SMb21517	pK18*mobsacB*-*SMb21517*	ataaaagcttAAGATGTACAGCGCGCGG (fwd)<br>ataatctagaATCACGATGCTCTTAGGT (rev)<br>ataatctagaCCGGCGACGCGCCTCGTT (fwd)<br>ataaggatccGGTCTTATTGCGCTTGCG (rev)
SMc00887	pK18*mobsacB*-*SMc00887*	5
SMc03942	pK18*mobsacB*-*SMc03942*	5
SMb20900	pK18*mobsacB*-*SMb20900*	5
SMc03178	pK18*mobsacB*-*SMc03178*	5
SMc00992	pK18*mobsacB*-*SMc00992*	5
SMc00033	pK18*mobsacB*-*SMc00033*	5
SMc00038	pK18*mobsacB*-*SMc00038*	5
SMb20447	pK18*mobsacB*-*SMb20447*	5
SMa0137	pK18*mobsacB*-*SMa0137*	5
SMa1548	pK18*mobsacB*-*SMa1548*	5
SMc00074 (*rgsP*)	pK18*mobsacB*-*SMc00074*-AAL	atattctagaATGCCCCTGACCCGTAAG (fwd)<br>atataagcttTCAAGCCCGCTTCATCAG (rev)<br>Amplification from pABC-$rgsP_{\mathrm{AAL}}$[40]
SMc03141	pK18*mobsacB*-*SMc03141*	atataagcttTGCCGCCATCGAGACATTGTT (fwd)<br>atattctagaGATGAATTCCTCGCCGCCATA (rev)<br>atattctagaATCACGATGTTTTAGGGTCG (fwd)<br>atatggatccTTCGGCGGCGTCTGCGGTCCA (rev)

The table lists the *S. meliloti* Rm2011 genes containing PDE domains, the plasmids used for construction of the respective deletion strains, and either the primers used for plasmid construction or the plasmid source. pK18*mobsacB*-*SMc00074*-AAL contains a mutated gene encoding a variant of SMc00074 in which the 'EAL' motif essential for c-di-GMP degradation by PDE domains[41] is replaced for 'AAL' (E746A). All other plasmids contain 600-800 bp-long upstream and downstream flanking regions of the respective gene. Upstream flanking regions include the start codon plus ensuing 27 bp, downstream flanking regions include the last 27 bp plus ensuing stop codon. The primers given were used for amplification of the respective flanking regions from Rm2011 or, in case of *SMc00074*, for amplification of the gene variant from pABC-$rgsP_{\mathrm{AAL}}$[40]. Upper case letters in primer sequences indicate bases annealing to the PCR template, lower case

letters indicate tails containing restriction sites. Deletions and the *SMc00074*-AAL mutation were introduced into Rm2011 (*expR*$^-$) sequentially from top to bottom.

Quantification of c-di-GMP levels. Quantification of intracellular c-di-GMP levels was carried out as described by Burhenne and Kaever[42]. Briefly, nucleotides were extracted from cell pellets of 5ml TY cultures using 40% (v/v) acetonitrile, 40% (v/v) methanol, and 20% (v/v) water. Samples were then dried and subjected to liquid chromatography-tandem mass spectrometry (LC-MS/MS). Amounts of c-di-GMP detected were normalized to protein mass in the respective sample determined by Bradford assays.

Phenotype assays. Surface attachment was quantified with crystal violet staining as previously described[5]. In short, strains were grown to stationary phase in 30% MOPS-buffered medium (MOPS-buffered medium with nitrogen, carbon and phosphate sources reduced to 30%), diluted 1:10 in the same medium and subsequently grown in 96-well microtiter plates at 30 °C without shaking. After 2 days, cell densities were determined, the medium and unattached cells were removed, and the wells were washed with 200 µl water. Remaining (i.e., attached) cells were stained with 200 µl of aqueous 0.1% (m/v) crystal violet solution for 20 min at room temperature while gently shaking. The staining solution was then discarded, the wells washed twice with water, and the stain dissolved in 200 µl of 20% (v/v) acetone and 80% (v/v) ethanol for 20 minutes at room temperature. Staining of the solution was determined by measuring absorbance at 570 nm (A_{570}) with an Infinite M Plex microplate reader (Tecan) and normalized to OD_{600}.

Motility was quantified by spotting 2 µl of stationary phase cultures on a 1:5 diluted TY agar plate (final agar concentration 0.3% (m/v)). Plates were then incubated at 30 °C and imaged after 2 days. Spot diameters were measured with the Fiji/ImageJ image processing software.

Growth curves were determined by growing 3 biological replicates of each strain in the respective medium in a 96-well microtiter plate shaking at 200 rpm in an Infinite M Plex microplate reader (Tecan) set to 30 °C. Starter cultures had been grown in the same medium to stationary phase and diluted 1:100 for the experiment.

References

1. Zhang, R. *et al.* Structure of a bacterial quorum-sensing transcription factor complexed with pheromone and DNA. *Nature* **417**, 971–974 (2002).
2. Thompson, J. D., Higgins, D. G. & Gibson, T. J. CLUSTAL W: improving the sensitivity of progressive multiple sequence alignment through sequence weighting, position-specific gap penalties and weight matrix choice. *Nucleic Acids Res.* **22**, 4673–4680 (1994).
3. Charoenpanich, P., Meyer, S., Becker, A. & McIntosh, M. Temporal expression program of quorum sensing-based transcription regulation in *Sinorhizobium meliloti*. *J. Bacteriol.* **195**, 3224–3236 (2013).
4. diCenzo, G. C., MacLean, A. M., Milunovic, B., Golding, G. B. & Finan, T. M. Examination of prokaryotic multipartite genome evolution through experimental genome reduction. *PLoS Genet.* **10**, e1004742 (2014).
5. Schäper, S. *et al.* Cyclic di-GMP regulates multiple cellular functions in the symbiotic alphaproteobacterium *Sinorhizobium meliloti*. *J. Bacteriol.* **198**, 521–535 (2016).
6. Krol, E., Schäper, S. & Becker, A. Cyclic di-GMP signaling controlling the free-living lifestyle of alpha-proteobacterial rhizobia. *Biol. Chem.* **401**, 1335–1348 (2020).
7. Schäper, S. *et al.* A bifunctional UDP-sugar 4-epimerase supports biosynthesis of multiple cell surface polysaccharides in *Sinorhizobium meliloti*. *J. Bacteriol.* **201**, 1–15 (2019).
8. Dilanji, G. E., Teplitski, M. & Hagen, S. J. Entropy-driven motility of *Sinorhizobium*

meliloti on a semi-solid surface. *Proc. R. Soc. B Biol. Sci.* **281**, 20132575 (2014).

9. Gao, M., Coggin, A., Yagnik, K. & Teplitski, M. Role of specific quorum-sensing signals in the regulation of exopolysaccharide II production within *Sinorhizobium meliloti* spreading colonies. *PLoS One* **7**, e42611-13 (2012).

10. Nogales, J., Bernabéu-Roda, L., Cuéllar, V. & Soto, M. J. ExpR is not required for swarming but promotes sliding in *Sinorhizobium meliloti*. *J. Bacteriol.* **194**, 2027–2035 (2012).

11. Pellock, B. J., Teplitski, M., Boinay, R. P., Bauer, W. D. & Walker, G. C. A LuxR homolog controls production of symbiotically active extracellular polysaccharide II by *Sinorhizobium meliloti*. *J. Bacteriol.* **184**, 5067–5076 (2002).

12. Zanacchi, F. C. *et al.* A DNA origami platform for quantifying protein copy number in super-resolution. *Nat. Methods* **14**, 789–792 (2017).

13. Janczarek, M. Environmental signals and regulatory pathways that influence exopolysaccharide production in Rhizobia. *Int. J. Mol. Sci.* **12**, 7898–7933 (2011).

14. Grant, S. G. N., Jessee, J., Bloom, F. R. & Hanahan, D. Differential plasmid rescue from transgenic mouse DNAs into *Escherichia coli* methylation-restriction mutants. *Proc. Natl. Acad. Sci.* **87**, 4645–4649 (1990).

15. Simon, R., Priefer, U. & Pühler, A. A broad host range mobilization system for *in vivo* genetic engineering: Transposon mutagenesis in Gram-negative bacteria. *Bio/Technology* **1**, 784–791 (1983).

16. Studier, F. W., Rosenberg, A. H., Dunn, J. J. & Dubendorff, J. W. Use of T7 RNA polymerase to direct expression of cloned genes. in 60–89 (1990).

17. Bahlawane, C., McIntosh, M., Krol, E. & Becker, A. *Sinorhizobium meliloti* regulator MucR couples exopolysaccharide synthesis and motility. *Mol. Plant-Microbe Interact.* **21**, 1498–1509 (2008).

18. Bettenworth, V., McIntosh, M., Becker, A. & Eckhardt, B. Front-propagation in bacterial inter-colony communication. *Chaos An Interdiscip. J. Nonlinear Sci.* **28**, 106316 (2018).

19. Casse, F., Boucher, C., Julliot, J. S., Michel, M. & Dénarié, J. Identification and characterization of large plasmids in *Rhizobium meliloti* using agarose gel electrophoresis. *J. Gen. Microbiol.* **113**, 229–242 (1979).

20. McIntosh, M., Meyer, S. & Becker, A. Novel *Sinorhizobium meliloti* quorum sensing positive and negative regulatory feedback mechanisms respond to phosphate availability. *Mol. Microbiol.* **74**, 1238–1256 (2009).

21. Schäfer, A. *et al.* Small mobilizable multi-purpose cloning vectors derived from the *Escherichia coli* plasmids pK18 and pK19: selection of defined deletions in the chromosome of *Corynebacterium glutamicum*. *Gene* **145**, 69–73 (1994).

22. Tauch, A., Zheng, Z., Pühler, A. & Kalinowski, J. *Corynebacterium striatum* chloramphenicol resistance transposon Tn*5564*: genetic organization and transposition in *Corynebacterium glutamicum*. *Plasmid* **40**, 126–139 (1998).

23. Khan, S. R., Gaines, J., Roop, R. M. & Farrand, S. K. Broad-host-range expression vectors with tightly regulated promoters and their use to examine the influence of TraR and TraM expression on Ti plasmid quorum sensing. *Appl. Environ. Microbiol.* **74**, 5053–5062 (2008).

24. Schlüter, J.-P. *et al.* Classification of phenotypic subpopulations in isogenic bacterial

cultures by triple promoter probing at single cell level. *J. Biotechnol.* **198**, 3–14 (2015).

25. Karniel, A. *et al.* Co-translational folding intermediate dictates membrane targeting of the signal recognition particle receptor. *J. Mol. Biol.* **430**, 1607–1620 (2018).

26. Michel, E. & Wüthrich, K. High-yield *Escherichia coli*-based cell-free expression of human proteins. *J. Biomol. NMR* **53**, 43–51 (2012).

27. McIntosh, M., Serrania, J. & Lacanna, E. A novel LuxR-type solo of *Sinorhizobium meliloti*, NurR, is regulated by the chromosome replication coordinator, DnaA and activates quorum sensing. *Mol. Microbiol.* **112**, 678–698 (2019).

28. Kremers, G.-J., Goedhart, J., van Munster, E. B. & Gadella, T. W. J. Cyan and yellow super fluorescent proteins with improved brightness, protein folding, and FRET Förster radius. *Biochemistry* **45**, 6570–6580 (2006).

29. Bindels, D. S. *et al.* mScarlet: a bright monomeric red fluorescent protein for cellular imaging. *Nat. Methods* **14**, 53–56 (2017).

30. Kok, S. De *et al.* Rapid and reliable DNA assembly *via* ligase cycling reaction. *ACS Synth. Biol.* **3**, 97–106 (2014).

31. Shaner, N. C. *et al.* A bright monomeric green fluorescent protein derived from *Branchiostoma lanceolatum*. *Nat. Methods* **10**, 407–409 (2013).

32. Ho, S. N., Hunt, H. D., Horton, R. M., Pullen, J. K. & Pease, L. R. Site-directed mutagenesis by overlap extension using the polymerase chain reaction. *Gene* **77**, 51–59 (1989).

33. Bahlawane, C., Baumgarth, B., Serrania, J., Ruberg, S. & Becker, A. Fine-tuning of galactoglucan biosynthesis in *Sinorhizobium meliloti* by differential WggR (ExpG)-, PhoB-, and MucR-dependent regulation of two promoters. *J. Bacteriol.* **190**, 3456–3466 (2008).

34. Rizzo, M. A. & Piston, D. W. High-contrast imaging of fluorescent protein FRET by fluorescence polarization microscopy. *Biophys. J.* **88**, L14–L16 (2005).

35. Shaner, N. C. *et al.* Improved monomeric red, orange and yellow fluorescent proteins derived from *Discosoma* sp. red fluorescent protein. *Nat. Biotechnol.* **22**, 1567–1572 (2004).

36. Huth, J. R. *et al.* Design of an expression system for detecting folded protein domains and mapping macromolecular interactions by NMR. *Protein Sci.* **6**, 2359–2364 (1997).

37. Zhou, P. & Wagner, G. Overcoming the solubility limit with solubility-enhancement tags: successful applications in biomolecular NMR studies. *J. Biomol. NMR* **46**, 23–31 (2010).

38. Döhlemann, J. *et al.* A family of single copy *repABC*-type shuttle vectors stably maintained in the alpha-proteobacterium *Sinorhizobium meliloti*. *ACS Synth. Biol.* **6**, 968–984 (2017).

39. Beyer, H. M. *et al.* AQUA cloning: A versatile and simple enzyme-free cloning approach. *PLoS One* **10**, e0137652-20 (2015).

40. Schäper, S. *et al.* Seven-transmembrane receptor protein RgsP and cell wall-binding protein RgsM promote unipolar growth in Rhizobiales. *PLOS Genet.* **14**, e1007594 (2018).

41. Schmidt, A. J., Ryjenkov, D. A. & Gomelsky, M. The ubiquitous protein domain EAL is a cyclic diguanylate-specific phosphodiesterase: enzymatically active and inactive EAL domains. *J. Bacteriol.* **187**, 4774–4781 (2005).

42. Burhenne, H. & Kaever, V. Quantification of cyclic dinucleotides by reversed-phase LC-MS/MS. in *Cyclic Nucleotide Signaling in Plants: Methods and Protocols* (ed. Gehring, C.) 27–37 (Humana Press, 2013). doi:10.1007/978-1-62703-441-8_3.
