# Supplementary material for: Frequency modulation of a bacterial quorum sensing response
Source: Nat Commun. 2022 May 19;13:2772. doi: 10.1038/s41467-022-30307-6 (PMC9120067; doi:10.1038/s41467-022-30307-6)
Supplement: Supplementary file 2 — Description of Additional Supplementary Files [file 41467_2022_30307_MOESM2_ESM.pdf]

Description of Additional Supplementary Files

File Name: Supplementary Movie 1
Description: Pulsatile activation of the *sinI* promoter in an *expR*$^-$ microcolony. Full time-lapse microscopy movie of the *expR*$^-$ microcolony used to demonstrate pulsatile expression from the *sinI* promoter in Fig. 1c. Expression rate over the whole experiment runtime is shown in Supplementary Fig. 3a (first column). The cells in the colony carry the P*sinI-mVenus* fusion at the chromosomal locus and the integrated suicide plasmid pK18*mob*3-P*wgeA-mCerulean* that also includes a P*trp-mCherry* fusion for image segmentation. (Left) mVenus channel false-coloured according to the NIS-elements 'red fire' lookup table displayed in Fig. 1c, (right) phase contrast channel of the same colony.

File Name: Supplementary Movie 2
Description: *sinI* expression pulse frequency is elevated in the wild type. Full time-lapse microscopy movie of a wild-type microcolony likewise carrying the chromosomal P*sinImVenus* fusion and the integrated pK18*mob*3-P*wgeA-mCerulean* with the P*trp-mCherry* fusion. Expression rate over the whole experiment runtime is shown in Supplementary Fig. 7e (right). Pulse frequency is clearly elevated in the wild type compared to the *expR*$^-$ microcolony shown in Movie 1, while pulse amplitudes greatly vary from cell to cell both in the wild-type and in the *expR*$^-$ background. (Left) mVenus channel false-coloured according to the NIS-elements 'red fire' lookup table displayed in Fig. 1c, (right) phase contrast channel of the same colony.

File Name: Supplementary Data_Custom code
Description: This folder contains a ReadMe Word file, 5 Matlab scripts with the custom code, a subfolder 'data' with subfolders containing data of all colonies analysed with respect to pulsing in the manuscript, and a Word file with tables linking the respective colonies/data to different genotypes/growth conditions.
