# Supplementary material for: Frequency modulation of a bacterial quorum sensing response
Source: Nat Commun. 2022 May 19;13:2772. doi: 10.1038/s41467-022-30307-6 (PMC9120067; doi:10.1038/s41467-022-30307-6)
Supplement: Supplementary file 5 — Supplementary Data_Custom code [file 41467_2022_30307_MOESM5_ESM.zip › Supplementary Data_Custom code/Supplementary Data_Custom code/1_PeakFinder_ReadMe.docx]

**peakFinder_Bettenworth et al**

Besides this ReadMe, the folder **‘Supplementary data_Custom code’** contains the **custom code**, the subfolder **‘data’ with subfolders containing data of all colonies analyzed with respect to pulsing in the manuscript**, and a Word file with tables linking the respective colonies/data to different genotypes/growth conditions. E.g., the subfolder **‘200812-18’** contains the data of the representative *expR^-^* colony (Fig. 1c, Supplementary Fig. 3a (first column), Supplementary Movie 1); the folder **‘200819-16’** contains the data of the representative wild-type colony (Supplementary Fig. 7e (right), Supplementary Movie 2).

**System requirements** for the custom code: Matlab with the ‘Signal Processing’ and ‘Statistics and Machine Learning’ Toolboxes; the custom code was written and run on Matlab version R2019a/b and not tested on any other Matlab version. No non-standard hardware is required.

The main script of the code is ‘get_peak_properties’; the additional scripts contain helper functions that require no direct user interactions. No installation is required.

The code extracts pulse properties from data files that result from pre-processing of time-lapse microscopy movies with Ilastik^1^, Schnitzcells^2^, and previously published custom-written Matlab scripts^3^; the output of these programs was stored as a .mat Matlab file (e.g., tree_200812-18.mat). It can be run in single colony and in batch mode, and in both cases a pulseData.xlsx table is stored in the experiment folder with detailed properties of individual peaks. In batch mode, an additional averagePulseData.xlsx is stored in the main folder with average properties for each colony.

To run the code, open ‘get_peak_properties’ from the Matlab ‘Home’ tab and make sure that the Matlab ‘Current directory’ is set to the ‘Supplementary data_Custom code’ folder. To process the data, simply run the ‘get_peak_properties’ script. To process other data, the path to the datafiles has to be changed in the code: for single colony mode, set ‘mainPath’ (line 16 of the script) to the folder of the experiment; for batch mode, set ‘mainPath’ to the folder that contains all experiment folders that are to be included in the analysis. In the ‘Editor’ tab, press run. Expected run time for demo colonies approx. 1min without plotting or in overview mode (see below). For comparison, the output data tables generated by the authors are included.

As described in Methods, we used the expression rate calculated over a sliding window of 11 time points (200 min) for our analysis, which in our tree data tables and, correspondingly, in ‘get_peak_properties’, is termed ‘PAElowitzDT5YmeanCenter’. In principle, several constraints like the minimal distance between peaks in hours, or a minimal height of the peak can be set (lines 22-28). However, the only constraint we used in our analysis was the minimal prominence of the peak to the left, i.e., a minimal increase in gene expression rate relative to the lowest value obtained since the last pulse, or since the beginning of the movie, whichever comes first; this threshold was set to 6.

In addition to the tables generated, results can be plotted in different ways (lines 30-35); if plotting single peaks, clicking into the figure will generate the subsequent plot. See below for plotMode ‘overview’ of the two demo colonies.

The mean pulse frequency plots we show in our main Figures each give ‘pulse frequency per (cell life time and) hour’ (column C in averagePulseData table) for nine colonies per strain/growth condition, with the bars indicating the mean. Plots of expression rates in Supplementary Figures correspond to the plots above, but without triangles marking the peaks, and without bold black line indicating the mean expression rate. Plots in Supplementary Figure 11a, c, e, g, i, and k show pulse amplitudes corresponding to ‘prominence’ (column D in individual pulseData.xlsx sheets).

References:

1. Berg, S. *et al.* ilastik: interactive machine learning for (bio)image analysis. *Nat. Methods* **16**, 1226–1232 (2019).

2. Young, J. W. *et al.* Measuring single-cell gene expression dynamics in bacteria using fluorescence time-lapse microscopy. *Nat. Protoc.* **7**, 80–88 (2011).

3. van Vliet, S. *et al.* Spatially correlated gene expression in bacterial groups: The role of lineage history, spatial gradients, and cell-cell interactions. *Cell Syst.* **6**, 496-507.e6 (2018).
