# Supplementary material for: Frequency modulation of a bacterial quorum sensing response
Source: Nat Commun. 2022 May 19;13:2772. doi: 10.1038/s41467-022-30307-6 (PMC9120067; doi:10.1038/s41467-022-30307-6)
Supplement: Supplementary file 5 — Supplementary Data_Custom code [file 41467_2022_30307_MOESM5_ESM.zip › Supplementary Data_Custom code/Supplementary Data_Custom code/2_Colonies and their respective genotypes or growth conditions.docx]

**Colonies and their respective genotypes/growth conditions**

- **For Fig. 2d**

| *expR*^-^ P*sinR** | *expR*^-^ native | *expR*^-^ *nurR*^++^ |
| --- | --- | --- |
| 200717-01 | 200717-22 | 200717-48 |
| 200717-02 | 200717-23 | 200717-50 |
| 200717-05 | 200717-24 | 200717-52 |
| 200724-04 | 200724-20 | 200724-45 |
| 200724-06 | 200724-27 | 200724-47 |
| 200724-09 | 200724-28 | 200724-68 |
| 200806-01 | 200806-19 | 200806-47 |
| 200806-03 | 200806-21 | 200806-56 |
| 200806-10 | 200806-25 | 200806-58 |

- **For Fig. 3a**

| rich | P-starv |
| --- | --- |
| 200923-68 | 200923-13 |
| 200923-71 | 200923-14 |
| 200923-72 | 200923-18 |
| 200928-66 | 200928-30 |
| 200928-78 | 200928-46 |
| 200928-79 | 200928-54 |
| 201002-38 | 201002-53 |
| 201002-40 | 201002-56 |
| 201002-45 | 201002-80 |

- **For Fig. 3b**

| *expR*^-^ | *expR*^-^ *dgc0* | *expR*^-^ *pde0* |
| --- | --- | --- |
| 200812-18 | 200812-03 | 200812-38 |
| 200812-24 | 200812-08 | 200812-43 |
| 200812-32 | 200812-14 | 200812-44 |
| 200819-24 | 200819-49 | 200819-70 |
| 200819-26 | 200819-54 | 200819-73 |
| 200819-27 | 200819-55 | 200819-74 |
| 200826-23 | 200826-40 | 200826-59 |
| 200626-31 | 200826-51 | 200826-63 |
| 200826-32 | 200826-52 | 200826-67 |

- **For Fig. 3c**

| *expR*^-^ | wt |
| --- | --- |
| 200819-24 | 200819-04 |
| 200819-26 | 200819-12 |
| 200819-27 | 200819-16 |
| 200821-62 | 200821-23 |
| 200821-65 | 200821-27 |
| 200821-71 | 200821-31 |
| 200826-23 | 200826-07 |
| 200626-31 | 200826-15 |
| 200826-32 | 200826-16 |

- **For Fig. 3d**

| rich | P-starv |
| --- | --- |
| 200923-45 | 200923-25 |
| 200923-46 | 200923-26 |
| 200923-58 | 200923-33 |
| 200928-88 | 200928-02 |
| 200928-89 | 200928-03 |
| 200928-92 | 200928-19 |
| 201002-19 | 201002-86 |
| 201002-20 | 201002-87 |
| 201002-26 | 201002-93 |

| wt | *dgc0* | *pde0* |
| --- | --- | --- |
| 200821-23 | 200821-01 | 200821-42 |
| 200821-27 | 200821-05 | 200821-44 |
| 200821-31 | 200821-11 | 200821-55 |
| 200828-35 | 200828-01 | 200828-47 |
| 200828-63 | 200828-11 | 200828-49 |
| 200828-66 | 200828-14 | 200828-58 |
| 200909-29 | 200909-01 | 200909-44 |
| 200909-31 | 200909-06 | 200909-46 |
| 200909-36 | 200909-21 | 200909-47 |
