# Supplementary material for: Frequency modulation of a bacterial quorum sensing response
Source: Nat Commun. 2022 May 19;13:2772. doi: 10.1038/s41467-022-30307-6 (PMC9120067; doi:10.1038/s41467-022-30307-6)
Supplement: Supplementary file 6 — Reporting Summary [file 41467_2022_30307_MOESM6_ESM.pdf]

nature portfolio

Corresponding author(s): Vera Bettenworth
Prof. Dr. Anke Becker

Last updated by author(s): Mar 8, 2022

Reporting Summary

Nature Portfolio wishes to improve the reproducibility of the work that we publish. This form provides structure for consistency and transparency in reporting. For further information on Nature Portfolio policies, see our Editorial Policies and the Editorial Policy Checklist.

Statistics

For all statistical analyses, confirm that the following items are present in the figure legend, table legend, main text, or Methods section.

n/a	Confirmed	
☐	☒	The exact sample size (n) for each experimental group/condition, given as a discrete number and unit of measurement
☐	☒	A statement on whether measurements were taken from distinct samples or whether the same sample was measured repeatedly
☐	☒	The statistical test(s) used AND whether they are one- or two-sided<br>*Only common tests should be described solely by name; describe more complex techniques in the Methods section.*
☒	☐	A description of all covariates tested
☐	☒	A description of any assumptions or corrections, such as tests of normality and adjustment for multiple comparisons
☐	☒	A full description of the statistical parameters including central tendency (e.g. means) or other basic estimates (e.g. regression coefficient) AND variation (e.g. standard deviation) or associated estimates of uncertainty (e.g. confidence intervals)
☐	☒	For null hypothesis testing, the test statistic (e.g. F, t, r) with confidence intervals, effect sizes, degrees of freedom and P value noted<br>*Give P values as exact values whenever suitable.*
☒	☐	For Bayesian analysis, information on the choice of priors and Markov chain Monte Carlo settings
☒	☐	For hierarchical and complex designs, identification of the appropriate level for tests and full reporting of outcomes
☐	☒	Estimates of effect sizes (e.g. Cohen's d, Pearson's r), indicating how they were calculated

Our web collection on statistics for biologists contains articles on many of the points above.

Software and code

Policy information about availability of computer code

Data collection	(Time-lapse) Microscopy 2D & 3D: NIS Elements Advanced Research version 4.13 (Nikon)<br>Flow Cytometry: BD FACSDiva 8.0.1 (BD)<br>Single Molecule Microscopy: Micro-manager 1.4 (https://micro-manager.org/Citing_Micro-Manager)<br>EMSA: Typhoon Scanner Control v5.0 (GE Healthcare)<br>Plate reader: i-control 1.8 SP1 (Lifescience Tecan)<br>Western blot imaging: ChemiDoc MP Imaging System (Biorad)

Data analysis	(Time-lapse) Microscopy 2D: NIS Elements Advanced Research version 4.13 (Nikon) for image stack cropping, a combination of Schnitzcells version 1.1, Ilastik version 1.3.3post3, and previously published custom-written Matlab scripts (van Vliet et al, 2018) for preprocessing, segmentation, tracking (optional) and data extraction, and a custom-written Matlab (R2019a/b) program for peak analysis based on the Matlab function 'peakfinder'.<br>Time-lapse Microscopy 3D: NIS Elements Advanced Research version 4.5 General Analysis module (Nikon)<br>Flow Cytometry: FlowJo 10.6.0 (BD)<br>Single Molecule Microscopy: ThunderSTORM plugin ImageJ version 2.0<br>Plate reader: Microsoft Excel Version 16.16.27<br>Western blot quantification: Gel Analysis plugin ImageJ version 2.0.0<br>Amino acid sequence alignment: Alignment with Clustal X 2.1 (https://www.ebi.ac.uk/Tools/msa/clustalo/) on June 23 2021, and further processed with Jalview 2.8.2 for visualization<br>Model of the AHL binding site of SinR: Model was generated with SWISSMODEL on June 23 2021, with SWISS-MODEL Template Library (SMTL) 2021-06-16 based on PDB release 2021-06-11. Structures were analyzed and images created with PyMOL (v1.5.0.5 Enhanced for Mac OS X, Schrödinger LLC).<br>Statistical analysis, correlations & regressions: Prism Version 9.1.1 (GraphPad)

For manuscripts utilizing custom algorithms or software that are central to the research but not yet described in published literature, software must be made available to editors and reviewers. We strongly encourage code deposition in a community repository (e.g. GitHub). See the Nature Portfolio guidelines for submitting code & software for further information.

Data

Policy information about availability of data

All manuscripts must include a data availability statement. This statement should provide the following information, where applicable:

- Accession codes, unique identifiers, or web links for publicly available datasets
- A description of any restrictions on data availability
- For clinical datasets or third party data, please ensure that the statement adheres to our policy

Amino acid sequence alignment in Supplementary Fig. 1: sequences downloaded from Uniprot on June 23 2021
SmeSinR, Sinorhizobium meliloti SinR (Uniprot accession number Q92PD1); SmeExpR, S. meliloti ExpR (Q2HY11); AtuTraR, Agrobacterium tumefaciens TraR (P33905); SfrTraR, Sinorhizobium fredii NGR234 TraR (P55407); CviCviR, Chromobacterium violaceum CviR (D3W065); AfiLuxR, A. fischeri LuxR (P12746); PaeLasR, Pseudomonas aeruginosa LasR (P25084); PaeRhlR, P. aeruginosa RhlR (P54292); PaeQscR, P. aeruginosa QscR (Q9RMS5); EcoSdiA, Escherichia coli SdiA (P07026).
Model of the AHL binding site of SinR in Supplementary Fig. 1: TraR structure downloaded from the protein data bank (PDB) under ID 1L1L on June 23 2021

The data generated in this study are provided in the ‘Source data’ file; pulse data are available in combination with the code in a Supplementary Data folder.

Field-specific reporting

Please select the one below that is the best fit for your research. If you are not sure, read the appropriate sections before making your selection.

☒ Life sciences ☐ Behavioural & social sciences ☐ Ecological, evolutionary & environmental sciences

For a reference copy of the document with all sections, see nature.com/documents/nr-reporting-summary-flat.pdf

Life sciences study design

All studies must disclose on these points even when the disclosure is negative.

Sample size	No statistical methods were used to predetermine sample size.<br>For (time-lapse) microscopy, 3 colonies were analyzed per experiment/biological replicate, and 3 biological replicates per strain/condition, making a total of >1000 cells per strain/condition over the whole data set. For flow cytometry, 15,000 events per experiment/biological replicate were analyzed, i.e., 45,000 per data set. For single molecule microscopy, >1000 cells per strain/condition were analyzed over the whole data set. These sample sizes were chosen in order to comply with standards in the literature, e.g., Locke et al, Science 2011 and Silander et al, Plos Genetics 2012.
Data exclusions	No data was excluded from the analysis.
Replication	If not stated otherwise, all data was gathered in 3 independent biological replicates/experiments. Microscopy on the PsinR-mCherry fusion (Fig. 2a), plate reader measurements on the effects of different culture supernatants (Fig. 4b) and western blots (Supplementary Fig. 7i) were performed in 2 biological replicates, since these already represented second methods (western blots confirming single molecule microscopy data which had been gathered in 3 biological replicates). All attempts at replication were successful.
Randomization	Not relevant to this study, since no experimental groups were generated.
Blinding	Not relevant to this study, since no experimental groups were generated.

Reporting for specific materials, systems and methods

We require information from authors about some types of materials, experimental systems and methods used in many studies. Here, indicate whether each material, system or method listed is relevant to your study. If you are not sure if a list item applies to your research, read the appropriate section before selecting a response.

Materials & experimental systems

n/a	Involved in the study	
☐	☒	Antibodies
☒	☐	Eukaryotic cell lines
☒	☐	Palaeontology and archaeology
☒	☐	Animals and other organisms
☒	☐	Human research participants
☒	☐	Clinical data
☒	☐	Dual use research of concern

Methods

n/a	Involved in the study	
☒	☐	ChIP-seq
☐	☒	Flow cytometry
☒	☐	MRI-based neuroimaging

Antibodies

Antibodies used	For detection of SinR: anti-FLAG M2-Peroxidase (horseradish peroxidase, HRP) antibody produced in mouse (1:1,000 dilution, Sigma Aldrich, A8592, monoclonal (clone M2), LOT# SLBB9238)<br>For detection of DnaK: anti-DnaK antibody raised in rabbit (1:20,000 dilution, Biorbyt Ldt, Cambridge, BYT-ORB53459, polyclonal, LOT# CB24490) as primary antibody; mouse anti-rabbit IgG-HRP (1:10,000 dilution, Santa Cruz Biotechnology, sc-2357, monoclonal, LOT# E2318) in a 1:10,000 dilution as secondary antibody.
Validation	Recognition of Flag-SinR by the anti-Flag M2-Peroxidase antibody was validated by comparing cell lysates of strains carrying the native sinR gene and the Flag-tagged sinR gene, respectively, on western blots; a corresponding image is shown in Supplementary Fig. 4a (left).<br>The anti-FLAG M2-Peroxidase (horseradish peroxidase, HRP) antibody and the anti-DnaK antibody are commercial antibodies and species-independent. The anti-FLAG M2-Peroxidase has been tested by the manufacturer, and exhibited an ELISA titer of > 20,000 and a limit of detection of 32 ng in dot blot when using 1:1000 dilution of the antibody conjugate. The anti-DnaK antibody has been tested by the manufacturers for application in western blots and ELISAs.

Flow Cytometry

Plots

Confirm that:

☒ The axis labels state the marker and fluorochrome used (e.g. CD4-FITC).

☒ The axis scales are clearly visible. Include numbers along axes only for bottom left plot of group (a 'group' is an analysis of identical markers).

☐ All plots are contour plots with outliers or pseudocolor plots.

☐ A numerical value for number of cells or percentage (with statistics) is provided.

Methodology

Sample preparation	Cultures were grown in 3 ml modified morpholinopropane sulfonate (MOPS)-buffered medium. 1 ml of cultures were harvested by centrifugation, resuspended in an equal volume of ice-cold phosphate-buffered saline, diluted to a final OD600 of 0.0125 in ice-cold PBS and kept on ice until analyzed.
Instrument	BD LSRFortessa SORP flow cytometer (BD Biosciences, Germany).
Software	BD FACSDiva 8.0.1(BD) for data collection, FlowJo 10.6.0 (BD) for analysis.
Cell population abundance	The 'singlets' gate resulting from exclusion of dead cells, debris and doublets (see below) usually contained >85% of events. Abundance of cells gated as 'positive' depended on strains/growth conditions.
Gating strategy	Gating (Supplementary Information Fig. 1) was first performed on forward and side scatters (FSC and SSC, respectively) to remove dead cells and debris (SSC-A over FSC-A) and to exclude doublets (SSC-W over SSC-H). Subsequently, using the FlowJo Exchange DownSample plugin, the number of samples was reduced to 15,000 events to ensure equal sample size. Strains lacking the sinI promoter-fluorophore gene fusion(s) with otherwise identical genetic backgrounds served as negative controls. Cells in the read-out samples with higher fluorescence intensities than those of the respective control cells were assessed as 'positive'. The fraction of cells per sample assessed as 'positive' and their corresponding median fluorescence values were determined. The gating strategy is exemplified in Supplementary Fig. 13.

☒ Tick this box to confirm that a figure exemplifying the gating strategy is provided in the Supplementary Information.
